# Disproportionation and Ligand Lability in Low Oxidation State Boryl‐Tin Chemistry**

**DOI:** 10.1002/chem.202203395

**Published:** 2023-01-04

**Authors:** Xiongfei Zheng, Agamemnon E. Crumpton, Andrey V. Protchenko, Andreas Heilmann, Mathias A. Ellwanger, Simon Aldridge

**Affiliations:** ^1^ Inorganic Chemistry Laboratory Department of Chemistry University of Oxford South Parks Road Oxford OX1 3QR UK

**Keywords:** boryl ligand, cluster, germanium, sub-valent compounds, tin

## Abstract

Boryltin compounds featuring the metal in the+1 or 0 oxidation states can be synthesized from the carbene‐stabilized tin(II) bromide (boryl)Sn(NHC)Br (boryl={B(NDippCH)_2_}; NHC=C{(N^*i*^PrCMe)_2_}) by the use of strong reducing agents. The formation of the mono‐carbene stabilized distannyne and donor‐free distannide systems (boryl)SnSn(IPrMe)(boryl) (**2**) and K_2_[Sn_2_(boryl)_2_] (**3**), using Mg(I) and K reducing agents mirrors related germanium chemistry. In contrast to their lighter congeners, however, systems of the type [Sn(boryl)]_*n*_ are unstable with respect to disproportionation. Carbene abstraction from **2** using BPh_3_, and two‐electron oxidation of **3** both result in the formation of a 2 : 1 mixture of the Sn(II) compound Sn(boryl)_2_, and the hexatin cluster, Sn_6_(boryl)_4_ (**4**). A viable mechanism for this rearrangement is shown by quantum chemical studies to involve a vinylidene intermediate (analogous to the isolable germanium compound, (boryl)_2_Ge=Ge), which undergoes facile atom transfer to generate Sn(boryl)_2_ and trinuclear [Sn_3_(boryl)_2_]. The latter then dimerizes to give the observed hexametallic product **4**, with independent studies showing that similar trigermanium species aggregate in analogous fashion.

## Graphical abstract

**The carbene‐stabilized distannyne** and donor‐free distannide systems (boryl)SnSn(IPrMe)(boryl) and K_2_[Sn_2_(boryl)_2_], can be synthesized from (boryl)Sn(NHC)Br using Mg(I) or K reducing agents. In contrast to their germanium congeners, systems of the type [Sn(boryl)]_*n*_ are unstable with respect to disproportionation: NHC abstraction from (boryl)SnSn(IPrMe)(boryl) and 2‐electron oxidation of K_2_[Sn_2_(boryl)_2_] both yield a 2 : 1 mixture of Sn(boryl)_2_ and the hexatin cluster, Sn_6_(boryl)_4_. A viable mechanism for this rearrangement is shown by quantum chemical studies to involve the vinylidene‐type intermediate (boryl)_2_Sn=Sn.

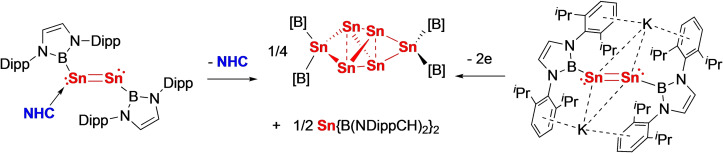

## Introduction

The organometallic chemistry of the group 14 elements in their lower oxidation states (≤+2) has been revolutionized by the development of a range of sterically encumbered ancillary ligands, which allows access to discrete molecular species that retain coordinative and electronic unsaturation. Among these, the heavier group 14 analogues of alkynes (dimetallynes, REER, E = Si − Pb) have become a topical area within the field of molecular main group chemistry.[^1^, ^2^, ^3^] In part, this is because these compounds (and related systems) posed a challenge to long‐held ideas about the feasibility of multiple bonding between heavier main group elements.^[4]^ In addition, due to their non‐linear (*trans*‐bent) structures and the resulting morphologies/energies of their frontier orbitals, dimetallynes can interact with a number of small molecule substrates in a manner comparable to transition metal complexes.[^3^, ^5^] This chemistry includes seminal examples of the activation of dihydrogen,^[6]^ and of organic substrates such as alkynes^[7]^ and alkenes[^6g^, ^8^] (in some cases reversibly).^[9]^

**Figure 1:**
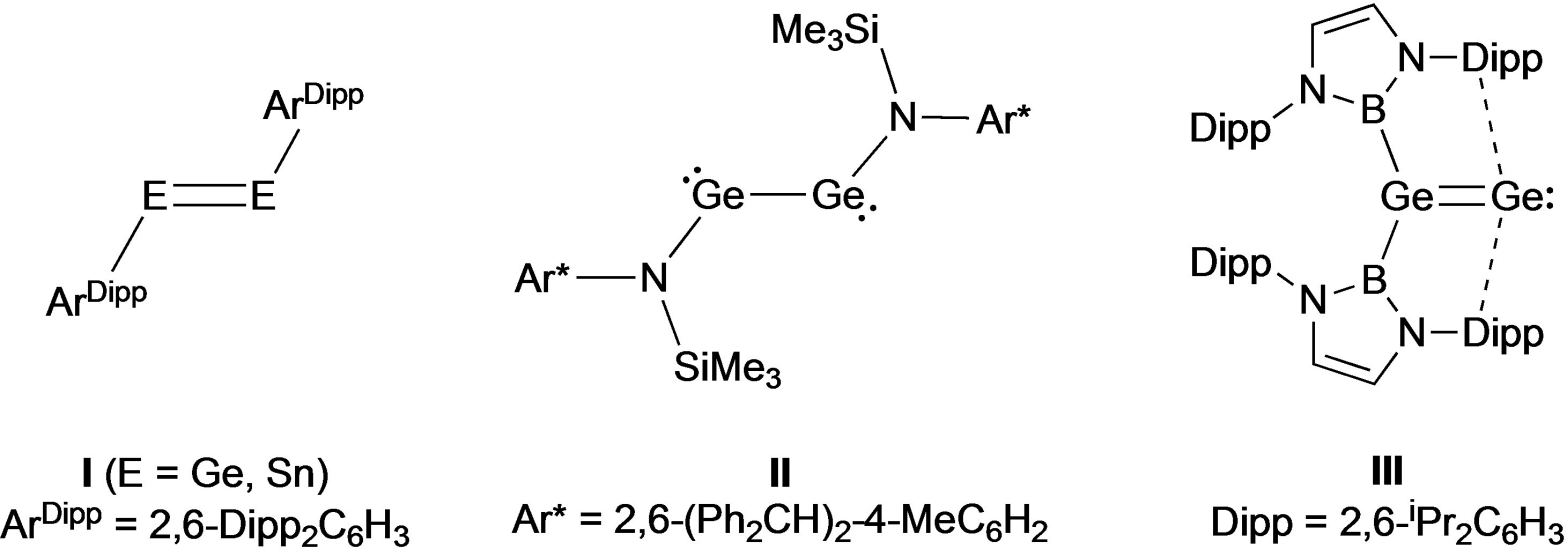
Systems of the stoichiometry (RE)_*n*_ (E=group 14 element) relevant to the current study.

Among the families of bulky monodentate ligands that have been employed to kinetically stabilize dimetallynes with respect to aggregation are strong σ‐donors such as aryl, silyl or amido substituents (e. g., **I** and **II**; Figure 1).[^1^, ^2^, ^6c^, ^6d^, ^6e^, ^6g^, ^7b^, ^10^, ^11^] As an alternative, we have recently been interested in the use of boryl ligands, −{B(NDippCH)_2_} to access main group systems featuring unusual electronic or geometric structure, and unprecedented modes of reactivity.^[12]^ With respect to compounds of the stoichiometry (RE)_*n*_ (E=group 14 element), it is noteworthy that the use of boryl substituents leads to the isolation of a compound featuring the alternative vinylidene structure, (boryl)_2_GeGe, in contrast to the digermyne isomers XGeGeX observed for X=aryl, amido etc. (**III**; Figure 1).[^6^, ^12h^] With this in mind, and given the varied structural and reaction chemistry reported for distannynes, we were interested in examining the consequences of the use of boryl ancillary ligands in the related chemistry of tin.

## Results and Discussion

**Figure 2:**
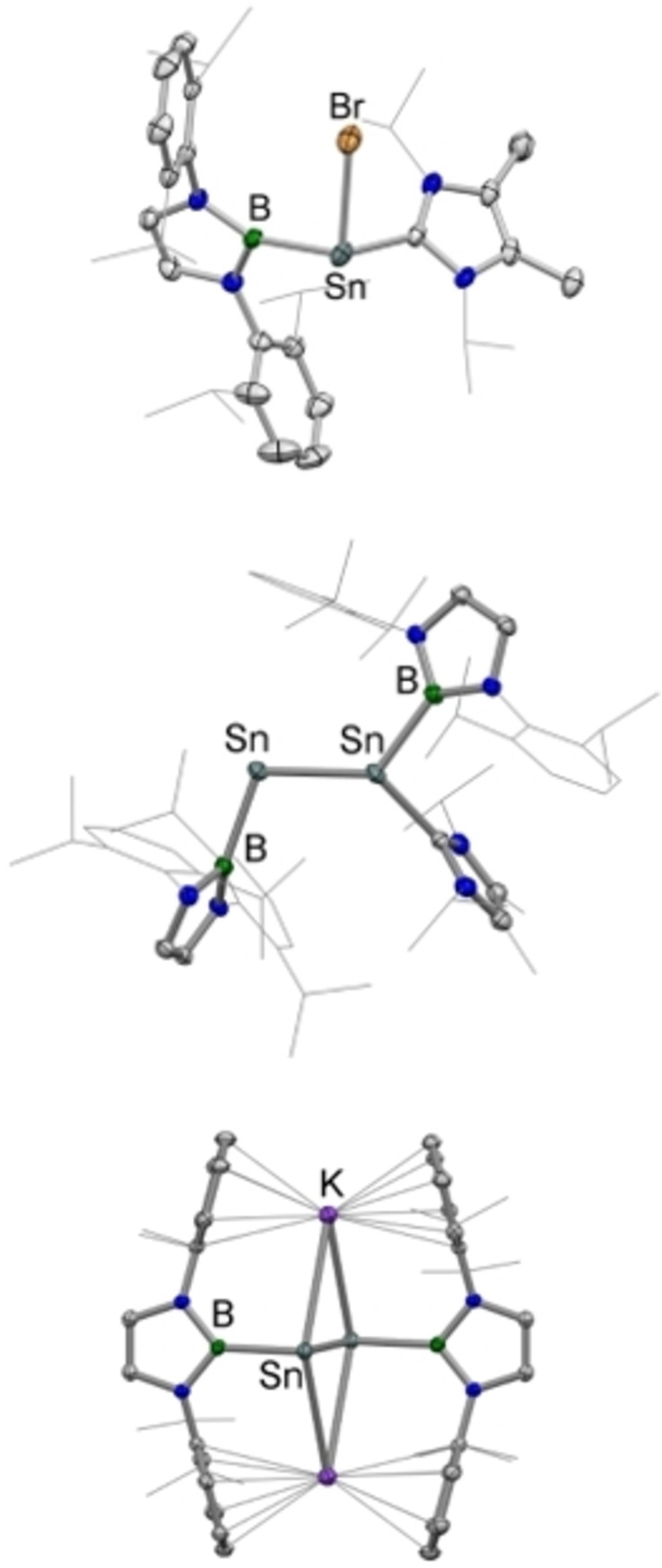
Molecular structures of {(HCDippN)_2_B}Sn(IPrMe)Br (**1**, upper), {(HCDippN)_2_B}SnSn(IPrMe){B(NDippCH)_2_} (**2**, centre) and K_2_[Sn_2_{B(NDipp CH)_2_}_2_] (**3**, lower) in the solid state as determined by X‐ray crystallography. Hydrogen atoms and solvate molecules omitted, and selected groups shown in wireframe format for clarity; thermal ellipsoids set at the 40 % probability level. Key bond lengths (Å) and angles (°): (for **1**) Sn−B 2.295(5), Sn−C 2.307(5), 2.6325(8), B−Sn‐Br 98.1(1), B−Sn‐C 97.4(2), C−Sn‐Br 94.6(1); (for **2**) Sn−Sn 2.581(1); Sn−B 2.243(7), 2.189(5); Sn−C 2.183(5); Sn‐Sn−B 109.1(2), 120.4(1); (for **3**) Sn−Sn 2.749(1); Sn−B 2.288(3); Sn K 3.599(1), 3.575(1); Sn‐Sn−B 96.5(1).

The N‐heterocyclic carbene stabilized (boryl)tin(II) bromide, {(HCDippN)_2_B}Sn(IPrMe)Br, **1** (Dipp=2,6‐^*i*^Pr_2_C_6_H_3_; IPrMe = C{(N^*i*^PrCMe)_2_}), is readily synthesized from (IPrMe)SnBr_2_ and {(HCDippN)_2_B}Li(thf)_2_ ^[13]^ in diethyl ether solution and can be isolated in ca. 60 % yield after recrystallization from toluene. **1** has been characterized by standard spectroscopic and analytical methods and its structure in the solid state determined by X‐ray crystallography. **1** is thus confirmed to be monomeric, featuring a three‐coordinate tin centre; the angles defined at the metal centre are close to 90°, as expected for a heavier *p*‐block element, with the slightly wider B−Sn‐Br and B−Sn−C angles (98.1(1) and 97.4(2)° vs. 94.6(1) for ∠C−Sn‐Br) presumably reflecting the high steric demands of the boryl ligand (Figure 2).

**Scheme 1:**
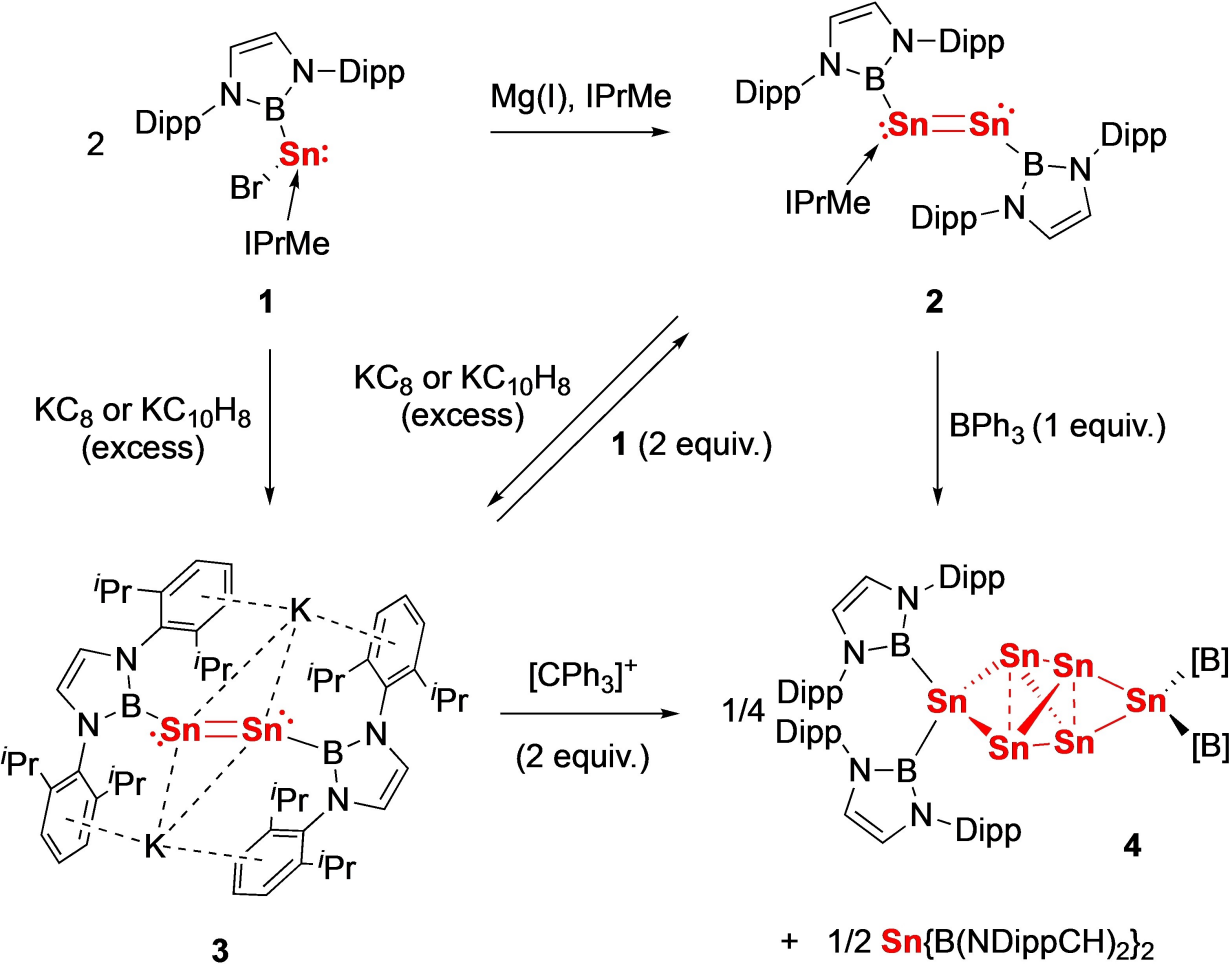
Reduction of {(HCDippN)_2_B}Sn(IPrMe)Br (**1**) by potassium or magnesium reducing agents to give {(HCDippN)_2_B}SnSn(IPrMe){B(NDippCH)_2_} (**2**) or K_2_[Sn_2_{B(NDippCH)_2_}_2_] (**3**); oxidation of **3** or carbene abstraction from **2** to yield a mixture of the tin cluster Sn_6_{B(NDippCH)_2_}_4_ (**4**) and diborylstannylene Sn{B(NDippCH)_2_}_2_ via a formal disproportionation process. [B]={B(NDippCH)_2_}.

**1** serves as a convenient precursor for tin‐centred reduction chemistry. As such, reaction with Jones’ Mg(I) reagent, [{HC(MeCMesN)_2_}Mg]_2_,^[14]^ yields the unsymmetrical mono NHC‐stabilized distannyne {(HCDippN)_2_B}SnSn(IPrMe){B(NDippCH)_2_} **2**, while more forcing conditions (excess KC_8_ or potassium naphthalenide) yield the more reduced (formally Sn(0)) compound K_2_[Sn_2_{B(NDippCH)_2_}_2_], **3** (Scheme 1). This reactivity finds precedent in the chemistry of the related borylgermanium compounds {(HCDippN)_2_B}GeGe(IPrMe){B(NDippCH)_2_} and K_2_[Ge_2_{B(NDippCH)_2_}_2_], which can be generated from {(HCDippN)_2_B}Ge(IPrMe)Cl under similar reduction conditions.^[12h]^ Solution‐phase multinuclear NMR data for **2** and **3** are consistent with a higher degree of molecular symmetry for the latter, *viz* one Dipp CH signal in the ^1^H NMR spectrum, and a single ^11^B NMR resonance at δ_B_=55.2 ppm (see two CH signals and two ^11^B resonances at δ_B_=43.5, 54.2 ppm for **2**). Integration of the ^1^H NMR signals associated with the IPrMe ligand in **2** is consistent with the presence of one carbene fragment per dimeric Sn_2_(boryl)_2_ unit. Studies of both compounds by X‐ray crystallography confirm the structures implied spectroscopically, and yield Sn−Sn distances of 2.581(1) and 2.749(1) Å, for **2** and **3**, respectively (Figure 2). The former distance is somewhat shorter that reported for the formal Sn=Sn double bond in Ar^Dipp^SnSnAr^Dipp^ (2.6675(4) Å), while the latter is in line with the slightly longer distances reported for doubly reduced terphenyl‐substituted distannynes (e. g., K_2_[Sn_2_Ar^Dipp^_2_]: 2.7754(3) Å).^[10a]^ In common with these systems, **3** also adopts a *trans* configuration about the Sn_2_ unit (∠Sn‐Sn−B=96.5(1)°), with the potassium counter‐ions being π‐bound between the flanking Dipp groups.

**Figure 3:**
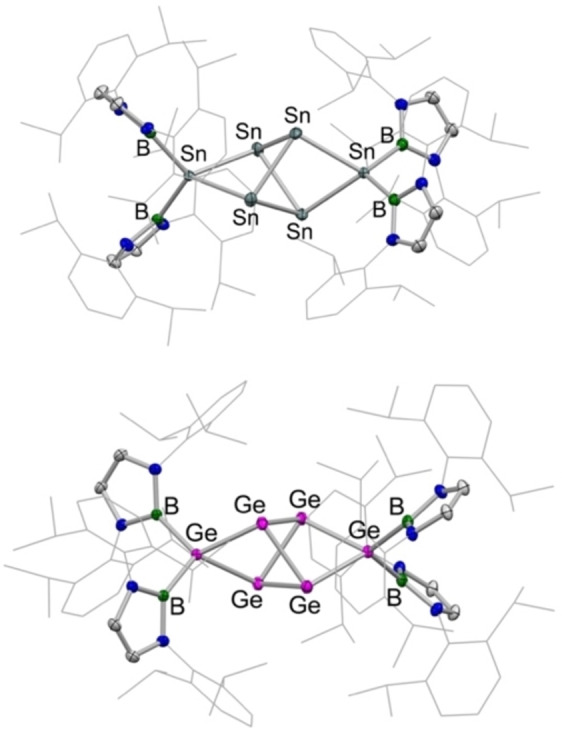
Molecular structures of Sn_6_{B(NDippCH)_2_}_4_ (**4**, upper) and Ge_6_{B(NDippCH)_2_}_4_ (**6**, lower) in the solid state as determined by X‐ray crystallography. Hydrogen atoms and solvate molecules omitted and selected groups shown in wireframe format for clarity; thermal ellipsoids set at the 40 % probability level. Key bond lengths (Å) and angles (°): (for **4**) Sn−B 2.259(6), 2.269(6); Sn(B)‐Sn 2.841(1), 2.834(1); Sn−Sn 2.848(1), 2.831(1); (for **6**) Ge−B 2.095(3), 2.105(3); Ge(B)‐Ge 2.500(1), 2.506(1); Ge−Ge 2.490(1), 2.468(1).

In contrast to the corresponding germanium system, which is inert to carbene abstraction under mild conditions,^[12h]^ removal of the remaining NHC ligand from **2** by the use of BPh_3_ proves to be synthetically viable (presumably due to the weaker nature of the Sn−C bond). Moreover, the products of this reaction are found to be identical to those obtained from **3** by the action of two equivalents of a trityl oxidant (Scheme 1). In each case, the two species formed (in a ratio of 2 : 1) are the known diborylstannylene Sn{B(NDippCH)_2_}_2_ ^[12f]^ and a new compound Sn_6_{B(NDippCH)_2_}_4_ (**4**), the structure of which was determined unambiguously by X‐ray crystallography (Figure 3). The molecular structure of **4** features four tin atoms arranged in distorted tetrahedral fashion, with opposite edges of the tetrahedron being elongated and bridged symmetrically by a [Sn{B(NDippCH)_2_}_2_] unit.[^15^, ^16^] The molecule sits on a *C*_2_ axis, with the Sn−Sn bonded distances within the tetrahedron (2.848(1), 2.831(1) Å) and the corresponding contacts involving the [Sn{B(NDippCH)_2_}_2_] units (2.841(1), 2.834(1) Å) all falling in the range expected for Sn−Sn single bonds. The Sn−B distances (2.259(6), 2.269(6) Å) are similar to those measured for Sn{B(NDippCH)_2_}_2_ itself.^[12f]^

**Figure 4:**
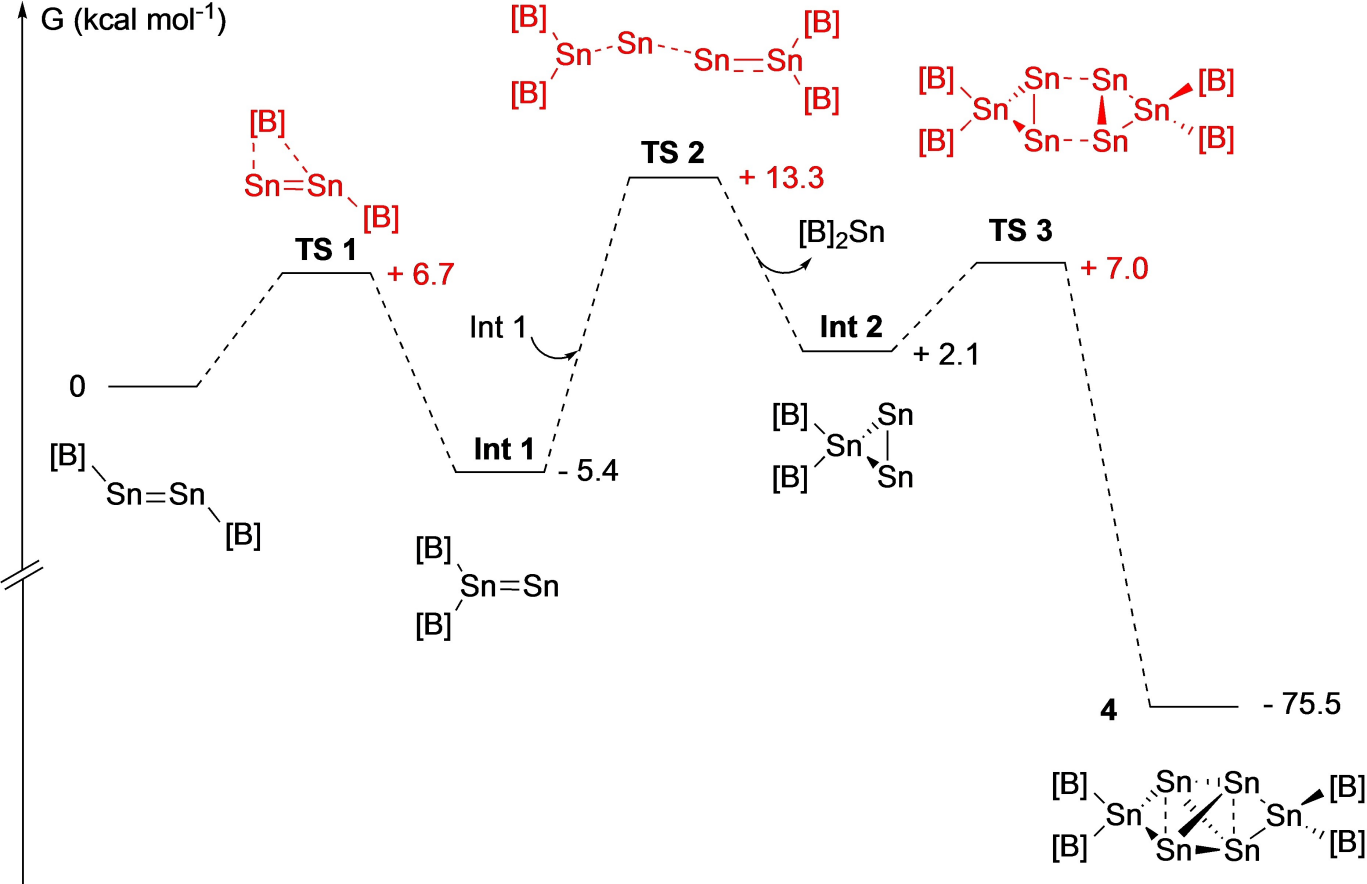
DFT calculated mechanism for the disproportionation of diboryldistannyne (formed by oxidation of **3** or NHC abstraction from **2**) into diborylstannylene Sn{B(NDippCH)_2_}_2_ and tin cluster Sn_6_{B(NDippCH)_2_}_4_. [B]={B(NDippCH)_2_}.

The formation of **4** in this fashion by the two‐electron oxidation of K_2_[Sn_2_{B(NDippCH)_2_}_2_], **3**, contrasts with the analogous reaction of K_2_[Ge_2_{B(NDippCH)_2_}_2_], which yields the digermavinylidene {(HCDippN)_2_B}_2_GeGe, via transient formation of the isomeric diboryldigermyne {(HCDippN)_2_B}GeGe{B(NDippCH)_2_} (which is calculated to be ca. 2–3 kcal mol^−1^ higher in energy).^[12h]^ With this in mind, the conversion of **3** to **4** was investigated by quantum chemical methods (Figure 4; see Supporting Information for computational details). A mechanism is proposed involving initial formation of the corresponding diboryldistannyne by oxidation of **3** (or removal of the carbene donor from **2**) followed by 1,2‐migration of a boryl group to form a distannavinylidene (**Int 1**). As with its germanium counterpart, this system lies lower in energy than the distannyne isomer (ΔG=−5.4 kcal mol^−1^) and is accessed via a low activation barrier (**TS1**, ΔG^≠^=6.7 kcal mol^−1^ compared to 9.5 kcal mol^−1^ in the germanium case).^[12h]^ Unlike its germanium counterpart, however, **Int1** cannot be isolated – an observation we ultimately attribute to the weaker nature of the Sn=Sn interaction. Tin atom transfer between two distannylvinylidene units can then occur to produce one of the experimentally observed products, Sn{B(NDippCH)_2_}_2_, and a second intermediate, **Int 2**, which features a triangular Sn_3_ unit. The conversion of **Int1** to **Int2** is endergonic with a moderate barrier (ΔG=7.5 kcal mol^−1^, ΔG^≠^=18.3 kcal mol^−1^) and is likely rate limiting. However, subsequent dimerization of **Int 2** to yield the other experimentally observed product (**4**) is a massively exergonic process and constitutes the thermodynamic driving force of the overall process (ΔG=−75.5 kcal mol^−1^).

**Scheme 2:**
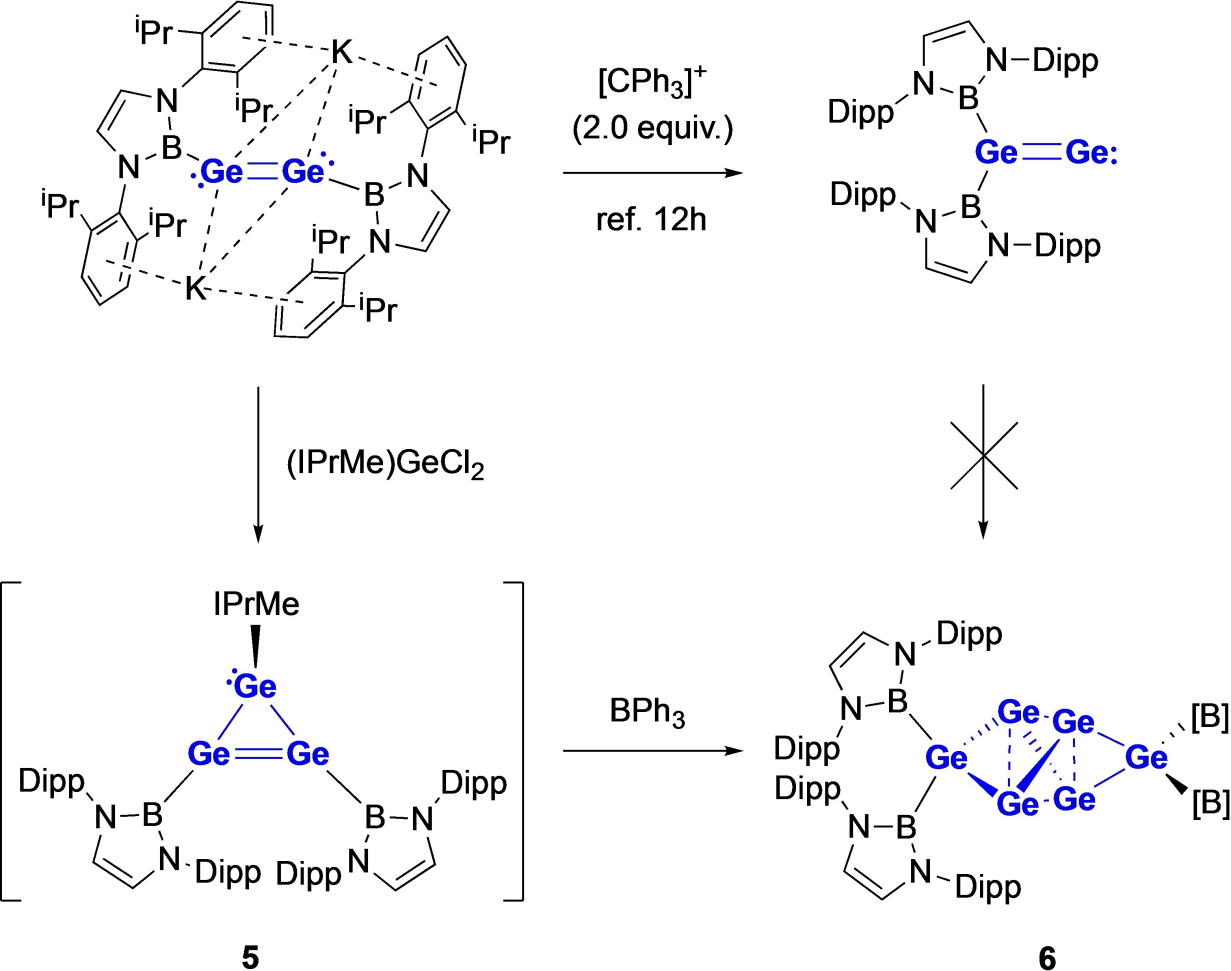
Synthesis of Ge_6_{B(NDippCH)_2_}_4_ (**6**), the germanium analogue of Sn_6_{B(NDippCH)_2_}_4_ (**4**). [B]={B(NDippCH)_2_}.

The hypothesis that Sn_6_{B(NDippCH)_2_}_4_ (**4**) is formed by the dimerization of a tri‐tin system also gains some experimental credence from aspects of the corresponding germanium chemistry. While digermvinylidene {(HCDippN)_2_B}_2_GeGe does not undergo spontaneous disproportionation in the manner of its putative tin counterpart (presumably due to the stronger Ge=Ge bond, compared to Sn=Sn), the reaction of K_2_[Ge_2_{B(NDippCH)_2_}_2_] with (IPrMe)GeCl_2_ yields an oily green compound, which is proposed to be the trigermanium system Ge_3_{B(NDippCH)_2_}_2_(IprMe) (**5**; Scheme 2) primarily on the basis of multinuclear NMR measurements and subsequent reactivity (see below). A combination of ^1^H and ^11^B NMR measurements suggests that the structure of **5** possesses two (equivalent) boryl groups and a single IPrMe carbene ligand. While **5** proved too labile to be isolated as a pure bulk substance for definitive structural characterization, it is susceptible to carbene abstraction using BPh_3_ to give a deep green product, which *can* be obtained as single crystals. Most revealingly (in the context of the tin chemistry outlined above) a combination of multinuclear NMR, micro‐analytical and crystallographic studies shows that this compound is Ge_6_{B(NDippCH)_2_}_4_ (**6**), i. e. the germanium analogue of Sn_6_{B(NDippCH)_2_}_4_ (**4**) (Figure 3).^[17]^ Structurally, **4** and **6** are very similar, differing primarily in the E−E and E−B bond lengths, at a level expected based on the differing covalent radii of germanium and tin (Δ*r*=1.39–1.20=0.19 Å).^[18]^ The formation of **6** in this way, suggests more broadly that systems of the stoichiometry E_3_{B(NDippCH)_2_}_2_ (E = Ge, Sn) are indeed prone to dimerization with accompanying boryl ligand migration.

## Conclusions

In conclusion we have shown that boryltin compounds featuring the metal in the formal oxidation states +1 and 0 can be accessed from a carbene‐stabilized tin(II) bromide precursor by the use of strong reducing agents. The formation of the mono‐carbene stabilized distannyne and donor‐free distannide systems (boryl)SnSn(IPrMe)(boryl) (**2**) and K_2_[Sn_2_(boryl)_2_], (**3**) mirrors related germanium chemistry occurring under similar conditions. In contrast to their lighter congeners, however, systems of the type [Sn(boryl)]_*n*_ are unstable with respect to disproportionation. Carbene abstraction from **2** using triphenylborane and two‐electron oxidation of **3** both result in the formation of a 2 : 1 mixture of the diborylstannylene, Sn(boryl)_2_, and the hexatin cluster, Sn_6_(boryl)_4_ (**4**). A viable mechanism for this rearrangement is shown by quantum chemical studies to proceed via a vinylidene intermediate (analogous to the isolable germanium compound, (boryl)_2_Ge=Ge), which undergoes facile atom transfer in the case of tin to generate Sn(boryl)_2_ and trinuclear [Sn_3_(boryl)_2_]. The latter then dimerizes to give the observed hexametallic product **4**. Independent studies also show that similar trigermanium species aggregate in analogous fashion.

## Experimental Section

### Syntheses of new compounds^[19]^

**{(HCDippN)_2_B}Sn(IPrMe)Br, 1**: To a suspension of (IPrMe)SnBr_2_ (0.70 g, 1.5 mmol) in Et_2_O (5 mL) at −35 °C was slowly added a pre‐cooled (−35 °C) solution of {(HCDippN)_2_B}Li ⋅ 2THF (0.82 g, 1.5 mmol) also in Et_2_O (5 mL). After stirring at this temperature for 30 min, the reaction mixture was slowly warmed to room temperature and stirred for another 1 h. Volatiles were removed under vacuum and the residue was extracted with toluene (5 mL). The filtrate was concentrated and triturated with pentane. After storing at −30 °C overnight, the pale yellow crystalline solid was isolated and dried under vacuum. Single crystals suitable for X‐ray crystallography were obtained from benzene. Yield: 0.72 g, 61.6 %. Anal. Calc. for C_37_H_56_BBrN_4_Sn: C 57.99 %, H 7.37 %, N 7.31 %; Meas.: C 57.68 %, H 7.48 %, N 7.00 %. ^1^H NMR (400 MHz, C_6_D_6_, 298 K): δ_H_ 1.00 (d, *J*_HH_=7.0 Hz, 6H, CH(C*H_3_*)_*2*_ of carbene), 1.07 (d, *J*_HH_=7.0 Hz, 6H, CH(C*H_3_*)_*2*_ of carbene), 1.10 (d, *J*_HH_=6.9 Hz, 6H, CH(C*H_3_*)_*2*_ of Dipp), 1.21 (d, *J*_HH_=6.9 Hz, 6H, CH(C*H_3_*)_*2*_ of Dipp), 1.30 (d, *J*_HH_=6.9 Hz, 6H, CH(C*H_3_*)_*2*_ of Dipp), 1.56 (s, 6H, CC*H_3_* of carbene), 1.59 (d, *J*_HH_=6.9 Hz, 6H, CH(C*H_3_*)_*2*_ of Dipp), 3.42 (sept, *J*_HH_=6.9 Hz, 2H, C*H*(CH_3_)_2_ of Dipp), 3.59 (sept, *J*_HH_=6.9 Hz, 2H, C*H*(CH_3_)_2_ of Dipp), 5.40 (sept, *J*_HH_=7.0 Hz, 2H, C*H*(CH_3_)_2_ of carbene), 6.37 (s, 2H, C*H* of boryl), 7.12 (t, *J*_HH_=4.6 Hz, 2H, *p*‐Ar*H* of Dipp), 7.24 (d, *J*_HH_=4.6 Hz, 4H, *m*‐Ar*H* of Dipp). ^11^B{^1^H} NMR (128 MHz, C_6_D_6_, 298 K): δ_B_ 38.8. ^13^C{^1^H} (151 MHz, C_6_D_6_, 298 K): δ_C_–10.0 (C *C* H_3_ of carbene), 20.9 and 22.3, (CH(*C* H_3_)_2_ of carbene), 23.5, 24.3, 26.2 and 26.6 (CH(*C* H_3_)_2_ of Dipp), 28.7 and 28.9 (*C* H(CH_3_)_2_ of Dipp), 54.4 (*C* H(CH_3_)_2_ of carbene), 122.7 (*C* H of boryl), 123.6 (*p*‐Ar of Dipp), 123.7 (*m‐*Ar of Dipp), 126.4 (*C* CH_3_ of carbene), 127.5 (*m‐*Ar of Dipp), 141.4 (*o‐*Ar of Dipp), 146.3 and 146.6 (*i‐*Ar of Dipp), 171.0 (R_2_ *C* of carbene). ^119^Sn{^1^H} NMR (112 MHz, C_6_D_6_, 298 K): δ_B_ 78.

**{(HCDippN)_2_B}SnSn(IPrMe){B(NDippCH)_2_}, 2**: A mixture of **1** (0.20 g, 0.26 mmol), IPrMe (0.047 g, 0.26 mmol) and [{HC(MeCMesN)_2_}Mg]_2_ (0.093 g, 0.13 mmol) was dissolved in toluene (5 mL) at room temperature. The colour of the reaction mixture immediately started to change to dark brown. After stirring for 30 min, volatiles were removed in vacuo, and the residue and was extracted with pre‐cooled pentane (−30 °C). After concentration, the filtrate was stored at −30 °C for ca. 2 d. The resulting black crystalline compound was isolated, washed with small amount of cold pentane and dried in vacuo. Yield: 0.120 g, 77.1 %. Crystals for X‐ray crystal structure determination were obtained from a saturated solution in hexane at 4 °C. Anal. Calc. for C_63_H_94_B_2_N_6_Sn_2_+0.5 C_5_H_12_: C 63.93 %, H 8.19 %, N 6.83 %; Meas.: C 64.50 %, H 7.76 %, N 6.48 %. ^1^H NMR (400 MHz, C_6_D_6_, 298 K): δ_H_ 0.83 (d, *J*_HH_=7.0 Hz, 12H, CH(C*H_3_*)_*2*_ of carbene), 1.17 (d, *J*_HH_=6.8 Hz, 24H, CH(C*H_3_*)_*2*_ of Dipp), 1.20 (br, overlapping, 24H, CH(C*H_3_*)_*2*_ of Dipp), 1.59 (s, 6H, CC*H_3_* of carbene), 3.32 (br, 4H, C*H*(CH_3_)_2_ of Dipp), 3.44 (br, 4H, C*H*(CH_3_)_2_ of Dipp), 5.45 (sept, *J*_HH_=7.3 Hz, 2H, C*H*(CH_3_)_2_ of carbene), 6.30 (br, 2H, C*H* of boryl), 6.44 (br, 2H, C*H* of boryl), 7.10 (m, 8H, *m*‐Ar*H* of Dipp), 7.19 (m, 4H, *p*‐Ar*H* of Dipp). ^11^B{^1^H} NMR (128 MHz, C_6_D_6_, 25 °C): δ_B_ 43.5, 54.2. ^13^C{^1^H} (151 MHz, C_6_D_6_, 298 K): δ_C_ 10.1 (C *C* H_3_ of carbene), 20.4 and 21.3 (CH(*C* H_3_)_2_ of carbene), 24.3, 25.7 and 26.1 (CH(*C* H_3_)_2_ of Dipp), 28.5 and 28.6 (*C* H(CH_3_)_2_ of Dipp), 54.7 (*C* H(CH_3_)_2_ of carbene), 122.3 (*C* H of boryl), 123.5 and 123.8 (*m‐*Ar of Dipp), 126.8 (*C* CH_3_ of carbene), 126.7 and 127.4 (*p*‐Ar of Dipp), 141.6 and 142.5 (*o*‐Ar of Dipp), 146.2 and 146.4 (*i‐*Ar of Dipp), 182.1 (R_2_ *C*: of carbene).

**K_2_[Sn_2_{B(NDippCH)_2_}_2_], 3, and Li_2_[Sn_2_{B(NDippCH)_2_}_2_]**: A mixture of **1** (0.20 g, 0.26 mmol) and KC_8_ (0.21 g, 1.6 mmol) was dissolved/suspended in toluene (5 mL) at room temperature. The reaction mixture was sonicated for 2 h with occasional manual stirring, resulting in a dark red solution. The reaction mixture can be filtered at this point and the filtrate concentrated and stored at −30 °C to give deep red crystals of **3** suitable for crystallography. **3** is not stable at room temperature and metathesis to give the more stable dilithium derivative allows for more convenient spectroscopic characterization: the dark red reaction mixture can alternatively be filtered into a Schlenk flask containing LiI (0.035 g, 0.29 mmol), and the resulting mixture stirred for 20 min. After filtration, the filtrate was concentrated (to ca. 1 mL) and layered with hexane (3 mL). Storing at 4 °C overnight gave dark purple crystals, which were isolated, washed with small amount of cold hexane and dried under vacuum. Yield of Li_2_[Sn_2_{B(NDippCH)_2_}_2_]: 0.035 g (0.05 mmol, 19.3 %). ^1^H NMR (400 MHz, C_6_D_6_, 298 K): δ_H_ 1.21 (m, 6H, CH(C*H_3_*)_*2*_ of carbene overlapping with pentane), 1.28 (d, *J*_HH_=6.9 Hz, 12H, CH(C*H_3_*)_*2*_ of Dipp), 1.35 (d, *J*_HH_=6.9 Hz, 12H, CH(C*H_3_*)_*2*_ of Dipp), 1.69 (s, 6H, CC*H_3_* of carbene), 3.94 (sept, *J*_HH_=6.9 Hz, 6H, C*H*(CH_3_)_2_ of Dipp overlapping with C*H*(CH_3_)_2_ of carbene), 6.58 (s, 2H, C*H* of boryl), 7.19 (m, 2H, *p*‐Ar*H* of Dipp), 7.21 (m, 4H, *m*‐Ar*H* of Dipp). ^11^B{^1^H} NMR (128 MHz, C_6_D_6_, 25 °C): δ_B_ 55.2 (br). ^13^C{^1^H} (151 MHz, C_6_D_6_, 298 K): δ_C_ 9.0 (C *C* H_3_ of carbene), 24.6 (CH(*C* H_3_)_2_ of carbene), 24.8 (CH(*C* H_3_)_2_ of Dipp), 25.3 (CH(*C* H_3_)_2_ of Dipp), 28.6 (*C* H(CH_3_)_2_ of Dipp), 50.0 (*C* H(CH_3_)_2_ of carbene), 121.3 (*C* H of boryl), 122.9 (*m‐*Ar of Dipp), 125.4 (*p*‐Ar of Dipp), 145.9 (*o*‐Ar of Dipp), 148.4 (*i‐*Ar of Dipp).

**Sn_6_{B(NDippCH)_2_}_4_, 4**: A mixture of **2** (0.10 g, 0.08 mmol) and BPh_3_ (20.3 mg, 0.08 mmol) was dissolved in toluene (5 mL) at room temperature. After stirring for 2 h, the reaction mixture was filtered into a layering Schlenk and concentrated (to ca. 1 mL). Pentane (15 mL) was then added on top of the filtrate and the mixture left for crystallization for several days. The supernatant was then decanted and the resulting deep red crystals washed with pentane and dried under vacuum. Yield: 20.0 mg, 21.1 %. Anal. Calc. for C_52_H_72_B_2_N_4_Sn_3_+0.4 C_5_H_12_: C 55.93 %, H 6.68 %, N 4.83 %; Meas.: C 56.56 %, H 6.31 %, N 4.78 %. ^1^H NMR (400 MHz, C_6_D_6_, 298 K): δ_H_ 0.77 (d, *J*_HH_=6.8 Hz, 6H, CH(C*H_3_*)_2_ of Dipp), 0.98 (d, *J*_HH_=6.8 Hz, 6H, CH(C*H_3_*)_2_ of Dipp), 1.16 (m, 12H, CH(C*H_3_*)_2_ of Dipp overlapping), 1.23 (d, *J*_HH_=6.8 Hz, 6H, CH(C*H_3_*)_2_ of Dipp), 1.26 (d, *J*_HH_=6.8 Hz, 6H, CH(C*H_3_*)_2_ of Dipp), 1.49 (d, *J*_HH_=6.8 Hz, 6H, CH(C*H_3_*)_2_ of Dipp), 1.57 (d, *J*_HH_=6.8 Hz, 6H, CH(C*H_3_*)_2_ of Dipp), 1.61 (d, *J*_HH_=6.8 Hz, 6H, CH(C*H_3_*)_2_ of Dipp), 1.57 (d, *J*_HH_=6.8 Hz, 6H, CH(C*H_3_*)_2_ of Dipp), 2.48 (sept, *J*_HH_=6.8 Hz, 2H, C*H*(CH_3_)_2_ of Dipp), 2.58 and 2.63 (m, 4H, C*H*(CH_3_)_2_ of Dipp overlapping), 3.59 (sept, *J*_HH_=6.8 Hz, 2H, C*H*(CH_3_)_2_ of Dipp), 6.05 and 6.21 (d, *J*_HH_=2.2 Hz, 4H, C*H* of boryl), 7.19 (m, 4H, *p*‐Ar*H* of Dipp), 7.30 (m, 8H, *m*‐Ar*H* of Dipp). ^11^B{^1^H} NMR (128 MHz, C_6_D_6_, 25 °C): δ_B_ 46.6. ^13^C{^1^H} (151 MHz, C_6_D_6_, 298 K): δ_C_ 21.5, 23.8, 27.0, 27.1, 27.2, 27.6 and 27.7 (CH(*C* H_3_)_2_ of Dipp), 27.9, 28.0, 28.2, 28.6 and 29.0 (*C* H(CH_3_)_2_ of Dipp), 123.8 (*C* H of boryl), 124.2 and 124.6 (*p*‐Ar of Dipp), 124.9 (*m*‐Ar of Dipp), 139.9, 141.3, 144.9 and 145.8 (*o*‐Ar of Dipp), 147.1 and 148.5 (*i‐*Ar of Dipp).

**Ge_3_{B(NDippCH)_2_}_2_(IPrMe), 5**: To a solution of K_2_[Ge_2_{B(NDippCH)_2_}_2_] (0.02 g, 0.02 mmol) in toluene at −35 °C was slowly added a solution of (IPrMe)GeCl_2_ (0.006 g, 0.02 mmol) also in toluene. After stirring for 10 min, the reaction mixture was slowly warmed to room temperature and stirred for another 20 min. Volatiles were removed under vacuum and the residue was extracted with pentane. The filtrate was then dried under vacuum and used for spectroscopic characterization. Yield: 0.015 g, 63.8 %. ^1^H NMR (400 MHz, C_6_D_6_, 298 K) δ 0.74 (s, 6H, CH(C*H_3_*)_*2*_ of carbene), 1.16, 1.22 and 1.24 (overlapping, 24H, CH(C*H_3_*)_2_ of Dipp), 1.44 (CC*H_3_* of carbene), 3.58 (br, 4H, C*H*(CH_3_)_2_ of Dipp), 6.06 (br, 1H, C*H*(CH_3_)_2_ of carbene), 6.33 (s, 2H, C*H* of boryl), 7.01 (m, 4H, *m*‐Ar*H* of Dipp), 7.11 (m, 2H, *p*‐Ar*H* of Dipp). ^11^B{^1^H} NMR (128 MHz, C_6_D_6_, 298 K): δ_B_ 36.5.

**Ge_6_{B(NDippCH)_2_}_4_, 6**: A mixture of **5** (15 mg, 0.013 mmol) and BPh_3_ (3.2 mg, 0.013 mmol) was dissolved in C_6_D_6_ (0.3 mL) at room temperature. After standing for 2 min, the reaction mixture was filtered into a NMR tube and concentrated (to ca. 0.1 mL). 0.5 mL of pentane was then added on top of the filtrate and the mixture left for crystallization overnight. The solution was then decanted and the resulting deep green crystals were washed with pentane and dried under vacuum. Yield: 7.1 mg, 55.0 %. Anal. Calc. for C_52_H_72_B_2_N_4_Ge_3_: C 62.92 %, H 7.31 %, N 5.64 %; Meas.: C 62.34 %, H 7.14 %, N 5.42 %. ^1^H NMR (400 MHz, C_6_D_6_, 298 K): δ_H_ 0.81 (d, *J*_HH_=6.8 Hz, 6H, CH(C*H_3_*)_2_ of Dipp), 0.93 (d, *J*_HH_=6.8 Hz, 6H, CH(C*H_3_*)_2_ of Dipp), 1.06 (d, *J*_HH_=6.8 Hz, 6H, CH(C*H_3_*)_2_ of Dipp), 1.20 (m, 18H, CH(C*H_3_*)_2_ of Dipp overlapping), 1.33 (d, *J*_HH_=6.8 Hz, 6H, CH(C*H_3_*)_2_ of Dipp), 1.57 (d, *J*_HH_=6.8 Hz, 6H, CH(C*H_3_*)_2_ of Dipp), 2.41 (sept, *J*_HH_=6.8 Hz, 2H, C*H*(CH_3_)_2_ of Dipp), 2.53, 2.57 (m, 4H, C*H*(CH_3_)_2_ of Dipp overlapping), 3.57 (sept, *J*_HH_=6.8 Hz, 2H, C*H*(CH_3_)_2_ of Dipp), 5.83, 5.91 (d, *J*_HH_=2.3 Hz, 4H, C*H* of boryl), 7.11 (m, 2H, Ar*H* of Dipp), 7.17 (m, 4H, Ar*H* of Dipp overlapping with C_6_D_6_), 7.30 (m, 6H, Ar*H* of Dipp). ^11^B{^1^H} NMR (128 MHz, C_6_D_6_, 25 °C): δ_B_ 33.3. ^13^C{^1^H} (151 MHz, C_6_D_6_, 298 K): δ_C_ 22.6, 23.8, 27.1, 27.3 and 27.5 (CH(*C* H_3_)_2_ of Dipp), 27.9, 28.1, 28.8 and 29.2 (*C* H(CH_3_)_2_ of Dipp), 123.5, 123.8 (*C* H of boryl), 124.0, 124.9 (*p*‐Ar of Dipp), 125.0, 125.1 (*m*‐Ar of Dipp), 139.9, 141.7, 145.0, 145.5 (*o*‐Ar of Dipp), 147.2, 148.7 (*i*‐Ar of Dipp).

**Quantum chemical calculations**: All computational work reported here was carried out at the density functional theory (DFT) level, using ORCA (Revision 5.0.1).[^20a^, ^20b^, ^20c^] The exchange correlation functional B3LYP[^20d^, ^20e^, ^20f^, ^20g^] was employed in conjunction with the LanL2DZ basis set with the D4 dispersion correction.[^20h^, ^20i^, ^20j^, ^20k^] All calculations were performed on model systems to reduce the computational cost, with ^i^Pr groups being replaced with Me groups. The nature of the stationary points, minima and transition states, was confirmed by full frequency calculations, and are characterized by zero or one imaginary frequency respectively along with full IRC (intrinsic reaction coordinate) calculations for transition states.

### Supporting Information

Full synthetic and characterizing data, representative NMR spectra, details of quantum chemical calculations and xyz files for optimized structures.

Deposition Number(s) 2215109 (**1**), 2215110 (**2**), 2215111 (**3**), 2215112 (**4**), 2215113 (**6**) contain(s) the supplementary crystallographic data for this paper. These data are provided free of charge by the joint Cambridge Crystallographic Data Centre and Fachinformationszentrum Karlsruhe Access Structures service

## Conflict of interest

The authors declare no conflict of interest.

1

## Supporting information

As a service to our authors and readers, this journal provides supporting information supplied by the authors. Such materials are peer reviewed and may be re‐organized for online delivery, but are not copy‐edited or typeset. Technical support issues arising from supporting information (other than missing files) should be addressed to the authors.

Supporting Information

## Data Availability

The data that support the findings of this study are available in the supplementary material of this article.
