# Supplementary material for: Disproportionation and Ligand Lability in Low Oxidation State Boryl‐Tin Chemistry
Source: Chemistry. 2023 Jan 4;29(10):e202203395. doi: 10.1002/chem.202203395 (PMC10947314; doi:10.1002/chem.202203395)
Supplement: Supplementary file 1 — Supporting Information [file CHEM-29-0-s001.pdf]

// Chemistry–A European Journal

Supporting Information

Disproportionation and Ligand Lability in Low Oxidation State Boryl-Tin Chemistry

Xiongfei Zheng, Agamemnon E. Crumpton, Andrey V. Protchenko, Andreas Heilmann, Mathias A. Ellwanger, and Simon Aldridge*

1. Synthetic/characterizing data and NMR spectra of novel compounds s2

2. Details of DFT calculations and *xyz* files s15

3. References for supporting information s22

1. Synthetic/characterizing data and NMR spectra of novel compounds

$\{(HCDippN)_2B\}Sn(IPrMe)Br$, 1. To a suspension of (IPrMe)$SnBr_2$ (0.70 g, 1.5 mmol) in Et_2O (amount) at -35 ^oC was slowly added a pre-cooled (-35 ^oC) solution of $\{(HCDippN)_2B\}Li\cdot 2THF$ (0.82 g, 1.5 mmol) also in Et_2O (amount). After stirring at this temperature for 30 min, the reaction mixture was slowly warmed to room temperature and stirred for another 1 h. Volatiles were removed under vacuum and the residue was extracted with toluene (amount). The filtrate was concentrated and triturated with pentane. After storing at -30 ^oC overnight, the pale yellow crystalline solid was isolated and dried under vacuum. Single crystals suitable for X-ray crystallography were obtained from xxx. Yield: 0.72 g, 61.6 %.

Anal. Calc. for $C_{37}H_{56}BBrN_4Sn$: C 57.99 %, H 7.37 %, N 7.31 %; Meas.: C 57.68 %, H 7.48 %, N 7.00 %.

^1H NMR (400 MHz, C_6D_6, 298 K): δ_H 1.00 (d, J_{HH} = 7.0 Hz, 6H, $CH(C\textit{H}_3)_2$ of carbene), 1.07 (d, J_{HH} = 7.0 Hz, 6H, $CH(C\textit{H}_3)_2$ of carbene), 1.10 (d, J_{HH} = 6.9 Hz, 6H, $CH(C\textit{H}_3)_2$ of Dipp), 1.21 (d, J_{HH} = 6.9 Hz, 6H, $CH(C\textit{H}_3)_2$ of Dipp), 1.30 (d, J_{HH} = 6.9 Hz, 6H, $CH(C\textit{H}_3)_2$ of Dipp), 1.56 (s, 6H, $CC\textit{H}_3$ of carbene), 1.59 (d, J_{HH} = 6.9 Hz, 6H, $CH(C\textit{H}_3)_2$ of Dipp), 3.42 (sept, J_{HH} = 6.9 Hz, 2H, $C\textit{H}(CH_3)_2$ of Dipp), 3.59 (sept, J_{HH} = 6.9 Hz, 2H, $C\textit{H}(CH_3)_2$ of Dipp), 5.40 (sept, J_{HH} = 7.0 Hz, 2H, $C\textit{H}(CH_3)_2$ of carbene), 6.37 (s, 2H, C*H* of boryl), 7.12 (t, J_{HH} = 4.6 Hz, 2H, *p*-Ar*H* of Dipp), 7.24 (d, J_{HH} = 4.6 Hz, 4H, *m*-Ar*H* of Dipp).

$^{11}B\{^1H\}$ NMR (128 MHz, C_6D_6, 298 K): δ_B 38.8.

$^{13}C\{^1H\}$ (151 MHz, C_6D_6, 298 K): δ_C 10.0 ($C\underline{C}H_3$ of carbene), 20.9 and 22.3, ($CH(\underline{C}H_3)_2$ of carbene), 23.5, 24.3, 26.2 and 26.6 ($CH(\underline{C}H_3)_2$ of Dipp), 28.7 and 28.9 ($\underline{C}H(CH_3)_2$ of Dipp), 54.4 ($\underline{C}H(CH_3)_2$ of carbene), 122.7 ($\underline{C}H$ of boryl), 123.6 (*p*-Ar of Dipp), 123.7 (*m*-Ar of Dipp), 126.4 ($\underline{C}CH_3$ of carbene), 127.5 (*m*-Ar of Dipp), 141.4 (*o*-Ar of Dipp), 146.3 and 146.6 (*i*-Ar of Dipp), 171.0 ($R_2\underline{C}$ of carbene).

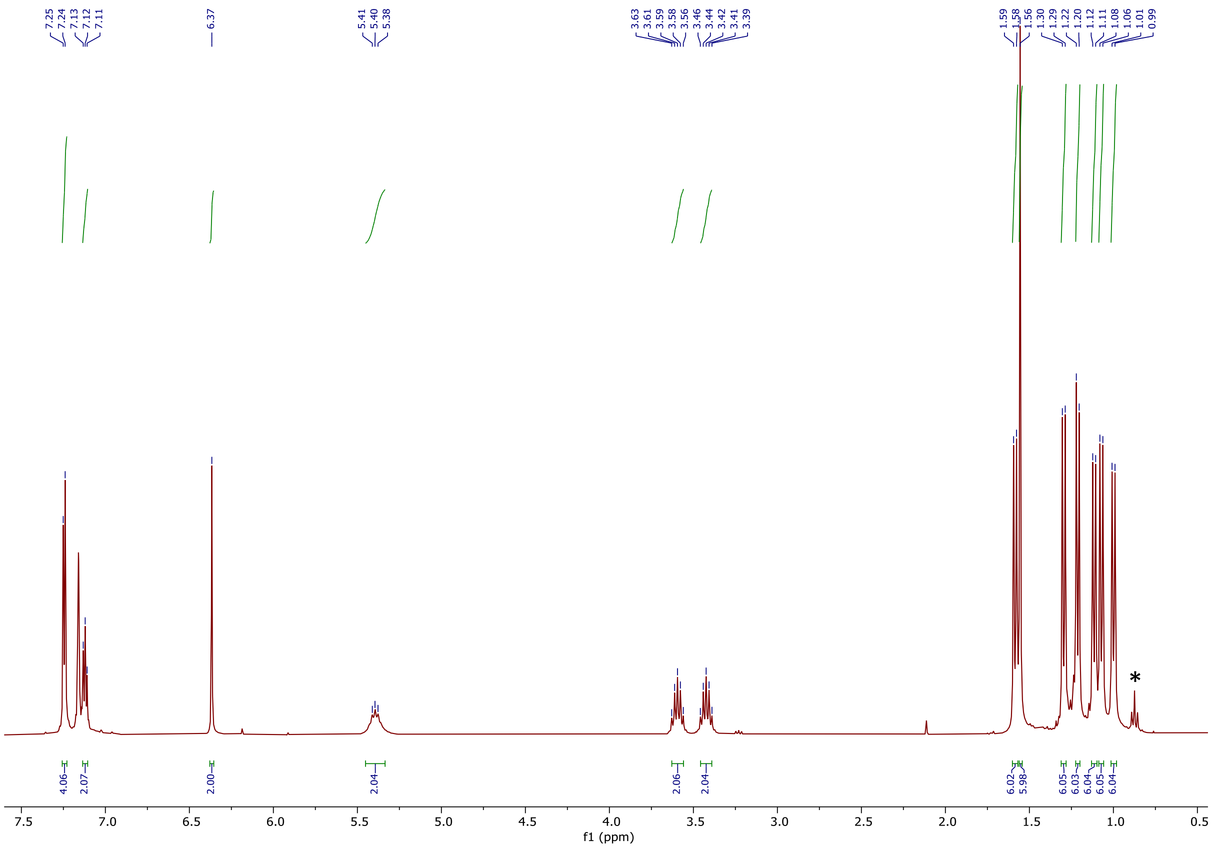
7.25
7.24
7.13
7.12
7.11
6.37
5.41
5.40
5.38
3.63
3.61
3.59
3.58
3.56
3.46
3.44
3.42
3.41
3.39
1.59
1.58
1.56
1.30
1.29
1.22
1.20
1.12
1.11
1.08
1.06
1.01
0.99
*
4.06
2.07
2.00
2.04
2.06
2.04
6.02
5.98
6.05
6.03
6.04
6.05
6.04
f1 (ppm)

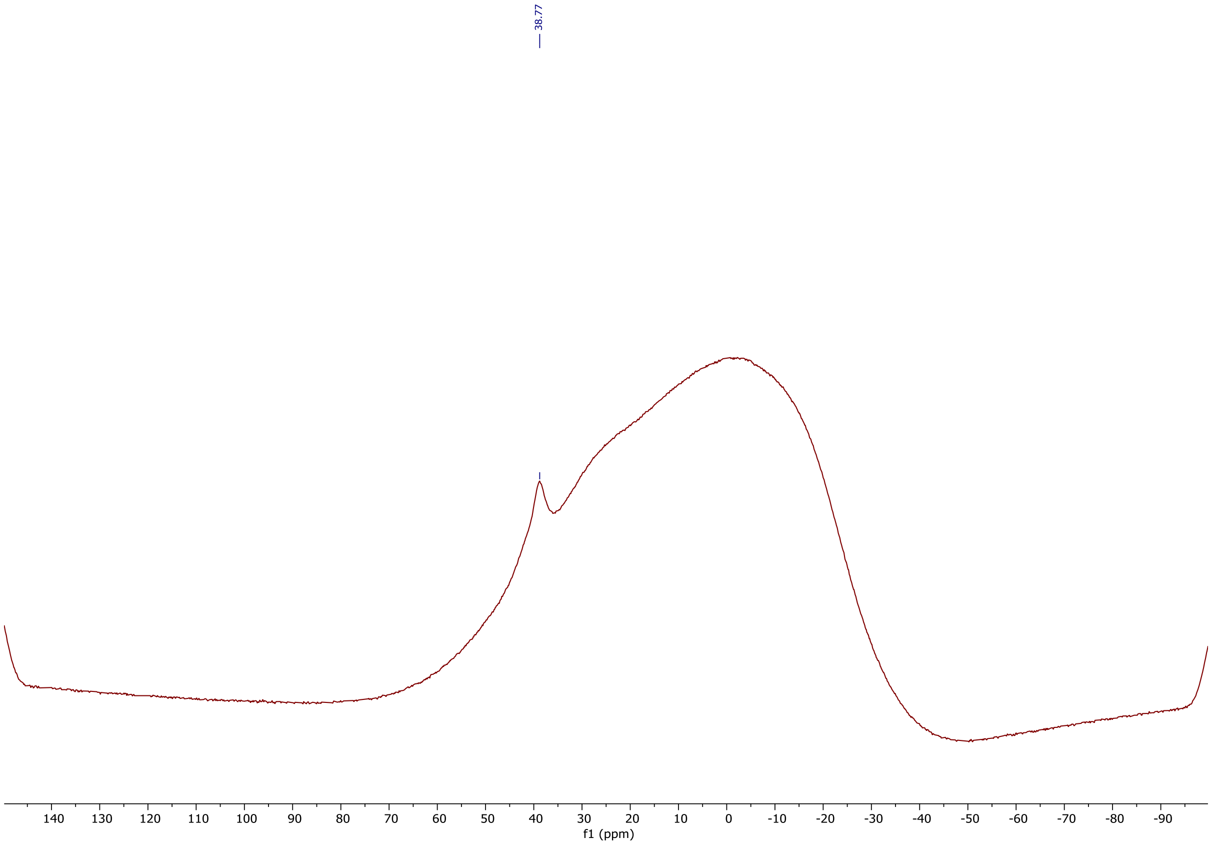
38.77
f1 (ppm)

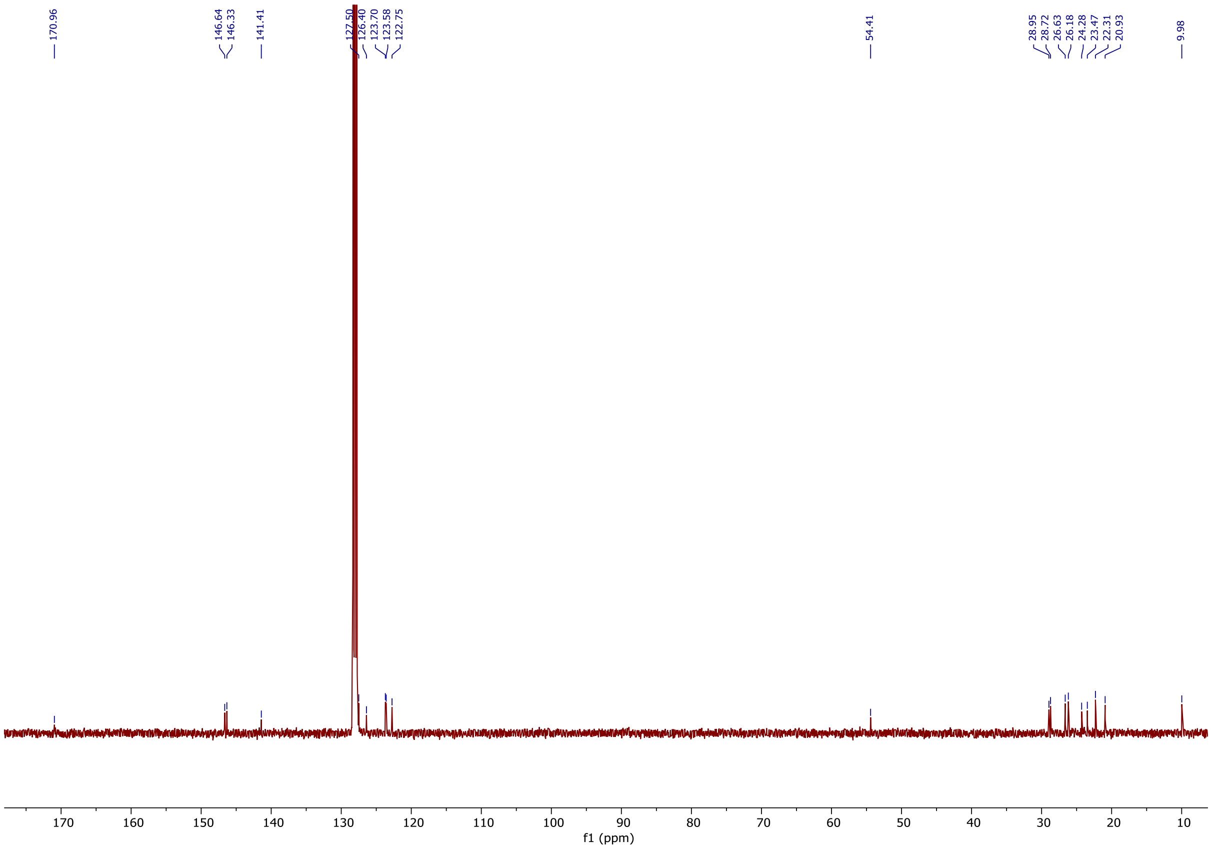

Figure s1: ^{1}H, $^{11}B\{^{1}H\}$ and $^{13}C\{^{1}H\}$ NMR spectra of **1** in C_6D_6. [* denotes minor pentane impurity]

{(HCDippN)$_2$B}SnSn(IPrMe){B(NDippCH)$_2$}, 2. A mixture of **1** (0.20 g, 0.26 mmol), IPrMe (0.047 g, 0.26 mmol) and [{HC(MeCMesN)$_2$}Mg]$_2$ (0.093 g, 0.13 mmol) was dissolved in toluene (5 mL) at room temperature. The colour of the reaction mixture immediately started to change to dark brown. After stirring for 30 min, volatiles were removed in vacuo, and the residue and was extracted with pre-cooled pentane (-30 °C). After concentration, the filtrate was stored at -30 °C for ca. 2 d. The resulting black crystalline compound was isolated, washed with small amount of cold pentane and dried in vacuo. Yield: 0.120 g, 77.1 %. Crystals for X-ray crystal structure determination were obtained from a saturated solution in hexane at 4 °C.

Anal. Calc. for $C_{63}H_{94}B_2N_6Sn_2$ + 0.5 C_5H_{12}: C 63.93 %, H 8.19 %, N 6.83 %; Meas.: C 64.50 %, H 7.76 %, N 6.48 %.

^{1}H NMR (400 MHz, C_6D_6, 298K): δ_H 0.83 (d, J_{HH} = 7.0 Hz, 12H, CH(C*H$_3$*)$_2$ of carbene), 1.17 (d, J_{HH} = 6.8 Hz, 24H, CH(C*H$_3$*)$_2$ of Dipp), 1.20 (br, overlapping, 24H, CH(C*H$_3$*)$_2$ of Dipp), 1.59 (s, 6H, CC*H$_3$* of carbene), 3.32 (br, 4H, C*H*(CH$_3$)$_2$ of Dipp), 3.44 (br, 4H, C*H*(CH$_3$)$_2$ of Dipp), 5.45 (sept, J_{HH} = 7.3 Hz, 2H, C*H*(CH$_3$)$_2$ of carbene), 6.30 (br, 2H, C*H* of boryl), 6.44 (br, 2H, C*H* of boryl), 7.10 (m, 8H, *m*-Ar*H* of Dipp), 7.19 (m, 4H, *p*-Ar*H* of Dipp).

^{11}B{^{1}H} NMR (128 MHz, C_6D_6, 25 °C): δ_B 43.5, 54.2.

^{13}C{^{1}H} (151 MHz, C_6D_6, 298K): δ_C 10.1 (C<u>C</u>H$_3$ of carbene), 20.4 and 21.3 (CH(<u>C</u>H$_3$)$_2$ of carbene), 24.3, 25.7 and 26.1 (CH(<u>C</u>H$_3$)$_2$ of Dipp), 28.5 and 28.6 (<u>C</u>H(CH$_3$)$_2$ of Dipp), 54.7 (<u>C</u>H(CH$_3$)$_2$ of carbene), 122.3 (<u>C</u>H of boryl), 123.5 and 123.8 (*m*-Ar of Dipp), 126.8 (<u>C</u>CH$_3$ of carbene), 126.7 and 127.4 (*p*-Ar of Dipp), 141.6 and 142.5 (*o*-Ar of Dipp), 146.2 and 146.4 (*i*-Ar of Dipp), 182.1 (R$_2$<u>C</u>: of carbene).

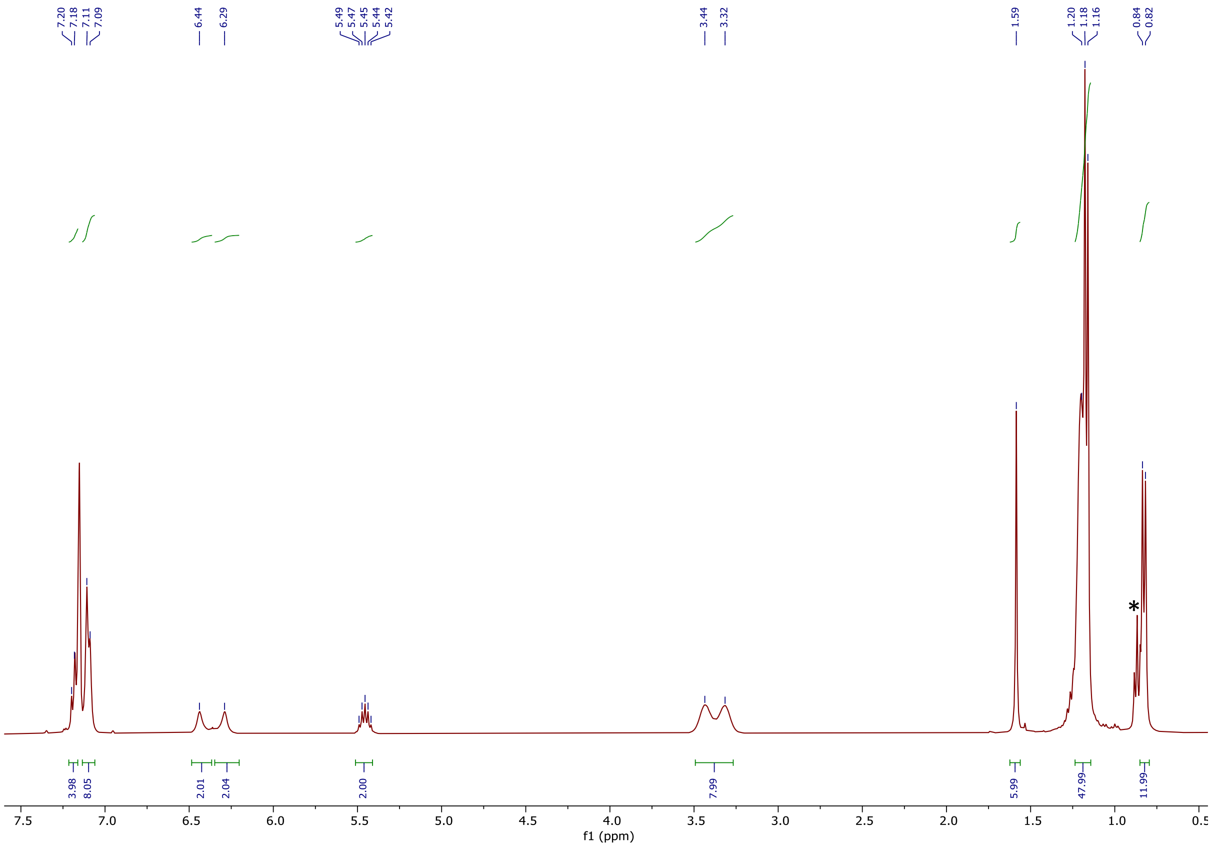

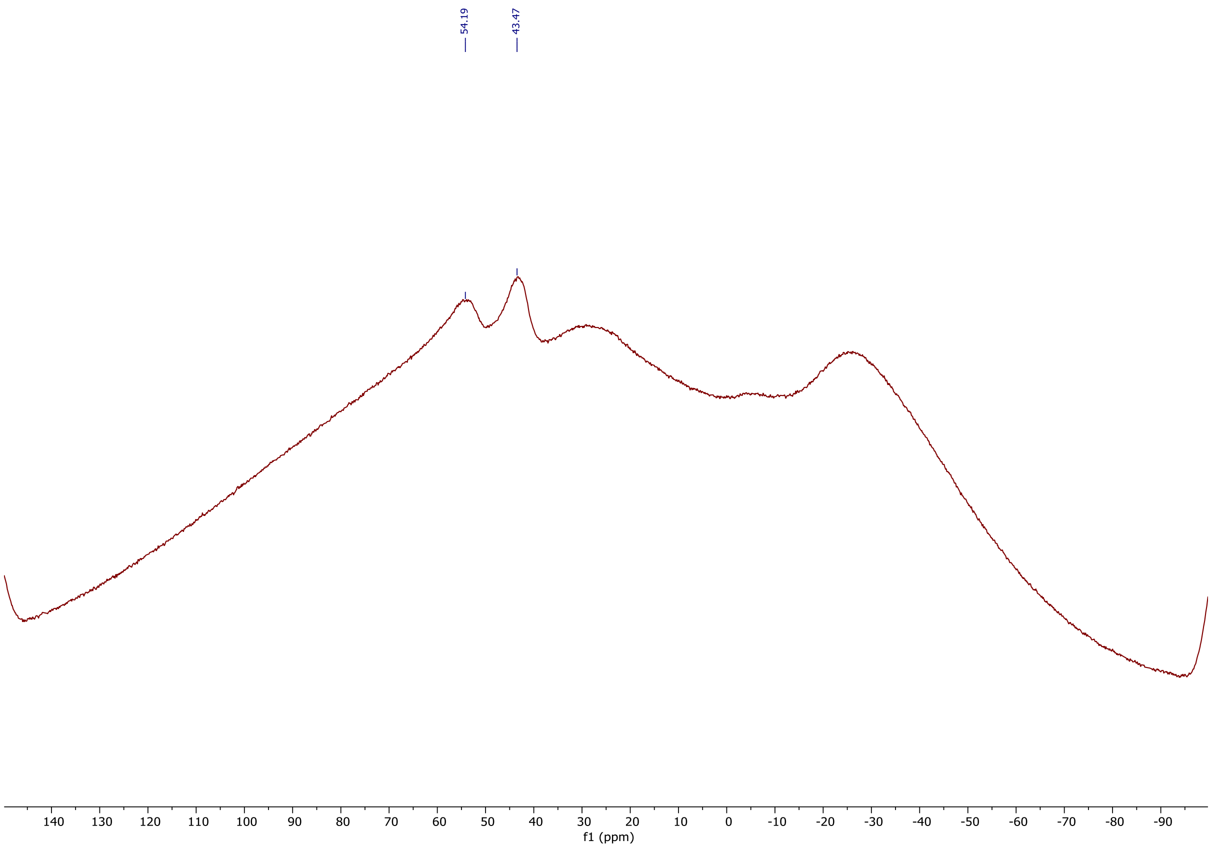

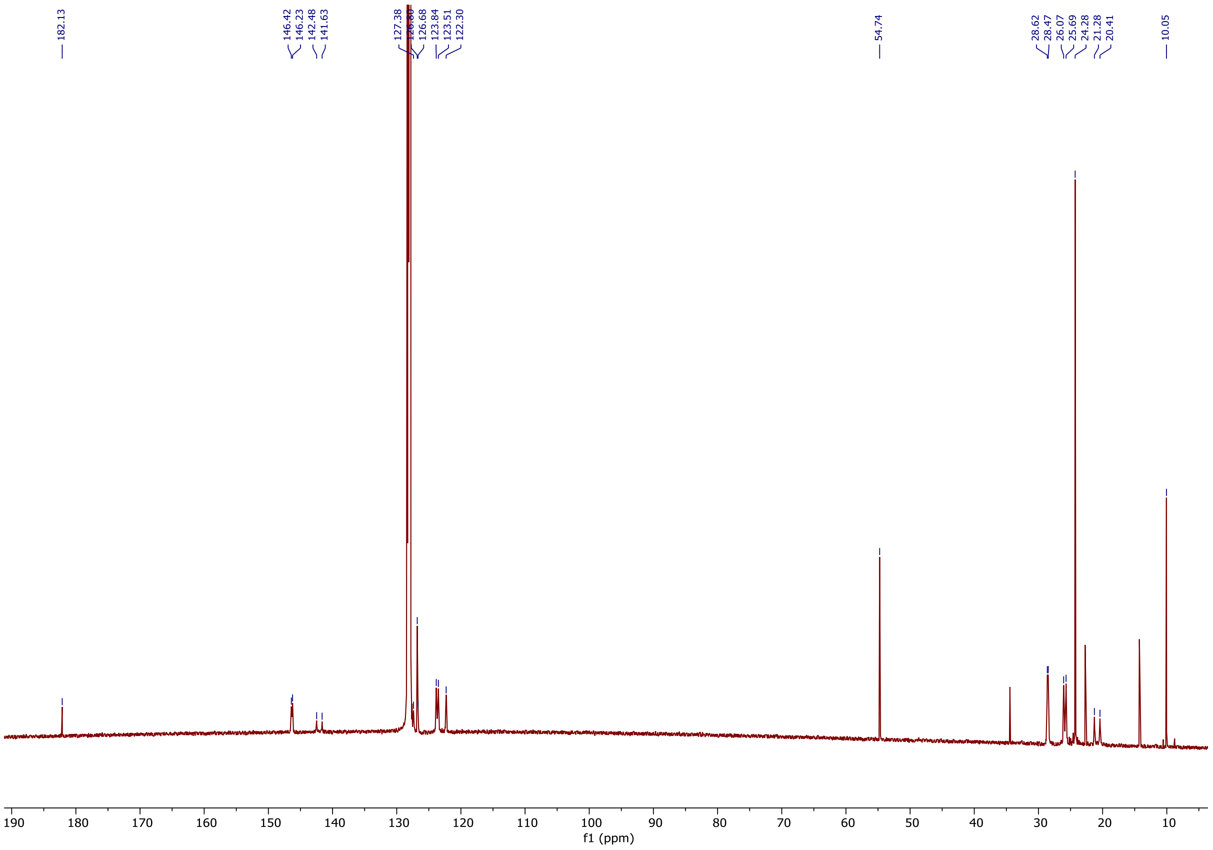

Figure s2: ^{1}H, $^{11}B\{^{1}H\}$ and $^{13}C\{^{1}H\}$ NMR spectra of **2** in C_6D_6. [* denotes minor pentane impurity]

$K_2[Sn_2\{B(NDippCH)_2\}_2]$, 3, and $Li_2[Sn_2\{B(NDippCH)_2\}_2]$. A mixture of **1** (0.20 g, 0.26 mmol) and KC_8 (0.21 g, 1.6 mmol) was dissolved/suspended in toluene (5 mL) at room temperature. The reaction mixture was sonicated for 2 h with occasional manual stirring, resulting in a dark red solution. The reaction mixture can be filtered at this point and the filtrate concentrated and stored at -30 °C to give deep red crystals of **3** suitable for crystallography. **3** is not stable at room temperature and metathesis to give the more stable dilithium derivative allows for more convenient spectroscopic characterization: the dark red reaction mixture can alternatively be filtered into a Schlenk flask containing LiI (0.035 g, 0.29 mmol), and the resulting mixture stirred for 20 min. After filtration, the filtrate was concentrated (to ca. 1 mL) and layered with hexane (3 mL). Storing at 4˚C overnight gave dark purple crystals, which were isolated, washed with small amount of cold hexane and dried under vacuum. Yield of $Li_2[Sn_2\{B(NDippCH)_2\}_2]$: 0.035 g (0.05 mmol, 19.3 %).

^{1}H NMR (400 MHz, C_6D_6, 298K): δ_H 1.21 (m, 6H, CH(C*H*$_3$)$_2$ of carbene overlapping with pentane), 1.28 (d, J_{HH} = 6.9 Hz, 12H, CH(C*H*$_3$)$_2$ of Dipp), 1.35 (d, J_{HH} = 6.9 Hz, 12H, CH(C*H*$_3$)$_2$ of Dipp), 1.69 (s, 6H, CC*H*$_3$ of carbene), 3.94 (sept, J_{HH} = 6.9 Hz , 6H, C*H*(CH$_3$)$_2$ of Dipp overlapping with C*H*(CH$_3$)$_2$ of carbene), 6.58 (s, 2H, C*H* of boryl), 7.19 (m, 2H, *p*-Ar*H* of Dipp), 7.21 (m, 4H, *m*-Ar*H* of Dipp).

^{11}B{^{1}H} NMR (128 MHz, C_6D_6, 25 °C): δ_B 55.2 (br).

^{13}C{^{1}H} (151 MHz, C_6D_6, 298K): δ_C 9.0 (C<u>C</u>H$_3$ of carbene), 24.6 (CH(<u>C</u>H$_3$)$_2$ of carbene), 24.8 (CH(<u>C</u>H$_3$)$_2$ of Dipp), 25.3 (CH(<u>C</u>H$_3$)$_2$ of Dipp), 28.6 (<u>C</u>H(CH$_3$)$_2$ of Dipp), 50.0 (<u>C</u>H(CH$_3$)$_2$ of carbene), 121.3 (<u>C</u>H of boryl), 122.9 (*m*-Ar of Dipp), 125.4 (*p*-Ar of Dipp), 145.9 (*o*-Ar of Dipp), 148.4 (*i*-Ar of Dipp).

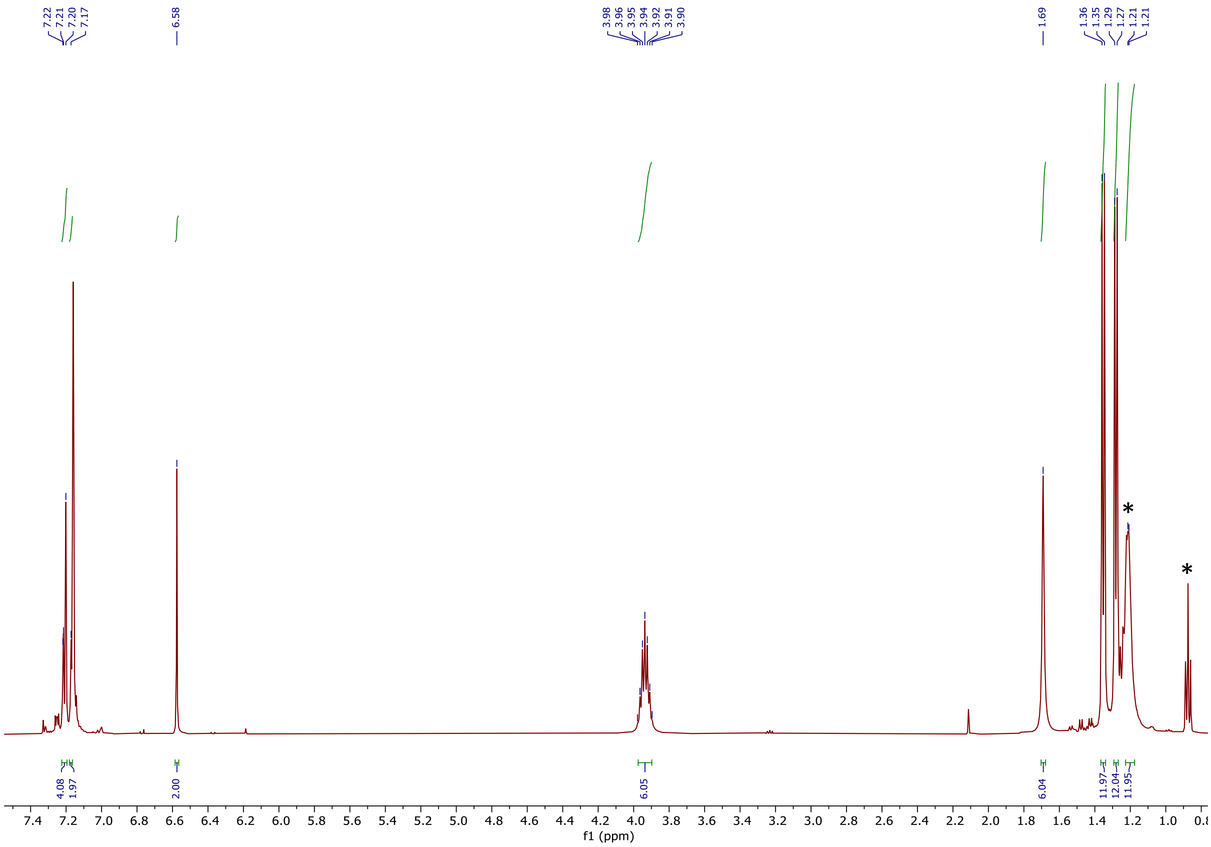

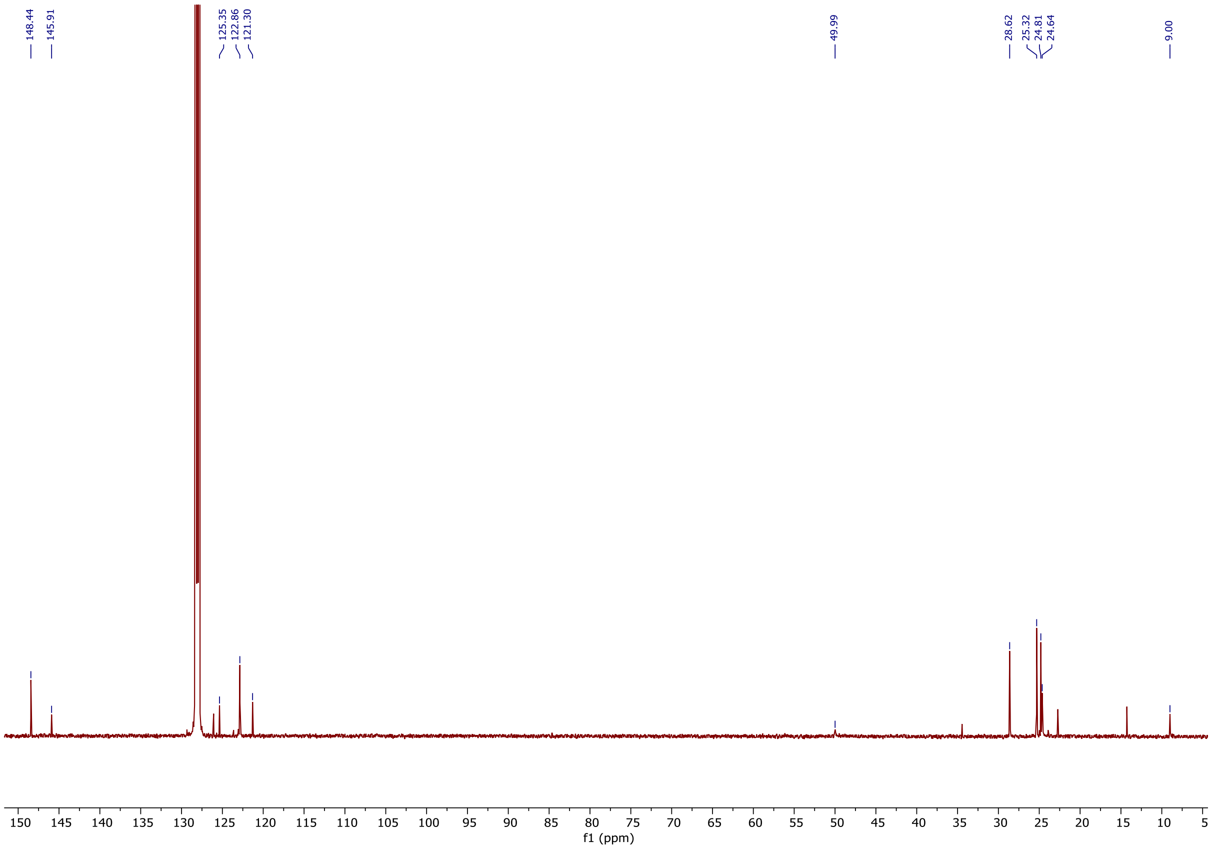

Figure s3: ^{1}H and $^{13}C\{^{1}H\}$ NMR spectra of $Li_2[Sn_2\{B(NDippCH)_2\}_2]$ in C_6D_6. [* denotes minor pentane impurity]

$Sn_6\{B(NDippCH)_2\}_4$, 4. A mixture of **2** (0.10 g, 0.08 mmol) and BPh_3 (20.3 mg, 0.08 mmol) was dissolved in toluene (5 mL) at room temperature. After stirring for 2 h, the reaction mixture was filtered into a layering Schlenk and concentrated (to ca. 1 mL). Pentane (15 mL) was then added on top of the filtrate and the mixture left for crystallization for several days. The supernatent was then decanted and the resulting deep red crystals washed with pentane and dried under vacuum. Yield: 20.0 mg, 21.1 %.

Anal. Calc. for $C_{52}H_{72}B_2N_4Sn_3 + 0.4\ C_5H_{12}$: C 55.93 %, H 6.68 %, N 4.83 %; Meas.: C 56.56 %, H 6.31 %, N 4.78 %.

^1H NMR (400 MHz, C_6D_6, 298 K): δ_H 0.77 (d, J_{HH} = 6.8 Hz, 6H, CH(C*H*$_3$)$_2$ of Dipp), 0.98 (d, J_{HH} = 6.8 Hz, 6H, CH(C*H*$_3$)$_2$ of Dipp), 1.16 (m, 12H, CH(C*H*$_3$)$_2$ of Dipp overlapping), 1.23 (d, J_{HH} = 6.8 Hz, 6H, CH(C*H*$_3$)$_2$ of Dipp), 1.26 (d, J_{HH} = 6.8 Hz, 6H, CH(C*H*$_3$)$_2$ of Dipp), 1.49 (d, J_{HH} = 6.8 Hz, 6H, CH(C*H*$_3$)$_2$ of Dipp), 1.57 (d, J_{HH} = 6.8 Hz, 6H, CH(C*H*$_3$)$_2$ of Dipp), 1.61 (d, J_{HH} = 6.8 Hz, 6H, CH(C*H*$_3$)$_2$ of Dipp), 1.57 (d, J_{HH} = 6.8 Hz, 6H, CH(C*H*$_3$)$_2$ of Dipp), 2.48 (sept, J_{HH} = 6.8 Hz, 2H, C*H*(CH$_3$)$_2$ of Dipp), 2.58 and 2.63 (m, 4H, C*H*(CH$_3$)$_2$ of Dipp overlapping), 3.59 (sept, J_{HH} = 6.8 Hz, 2H, C*H*(CH$_3$)$_2$ of Dipp), 6.05 and 6.21 (d, J_{HH} = 2.2 Hz, 4H, C*H* of boryl), 7.19 (m, 4H, *p*-Ar*H* of Dipp), 7.30 (m, 8H, *m*-Ar*H* of Dipp).

$^{11}B\{^1H\}$ NMR (128 MHz, C_6D_6, 25 °C): δ_B 46.6.

$^{13}C\{^1H\}$ (151 MHz, C_6D_6, 298 K): δ_C 21.5, 23.8, 27.0, 27.1, 27.2, 27.6 and 27.7 (CH(<u>C</u>H$_3$)$_2$ of Dipp), 27.9, 28.0, 28.2, 28.6 and 29.0 (<u>C</u>H(CH$_3$)$_2$ of Dipp), 123.8 (<u>C</u>H of boryl), 124.2 and 124.6 (*p*-Ar of Dipp), 124.9 (*m*-Ar of Dipp), 139.9, 141.3, 144.9 and 145.8 (*o*-Ar of Dipp), 147.1 and 148.5 (*i*-Ar of Dipp).

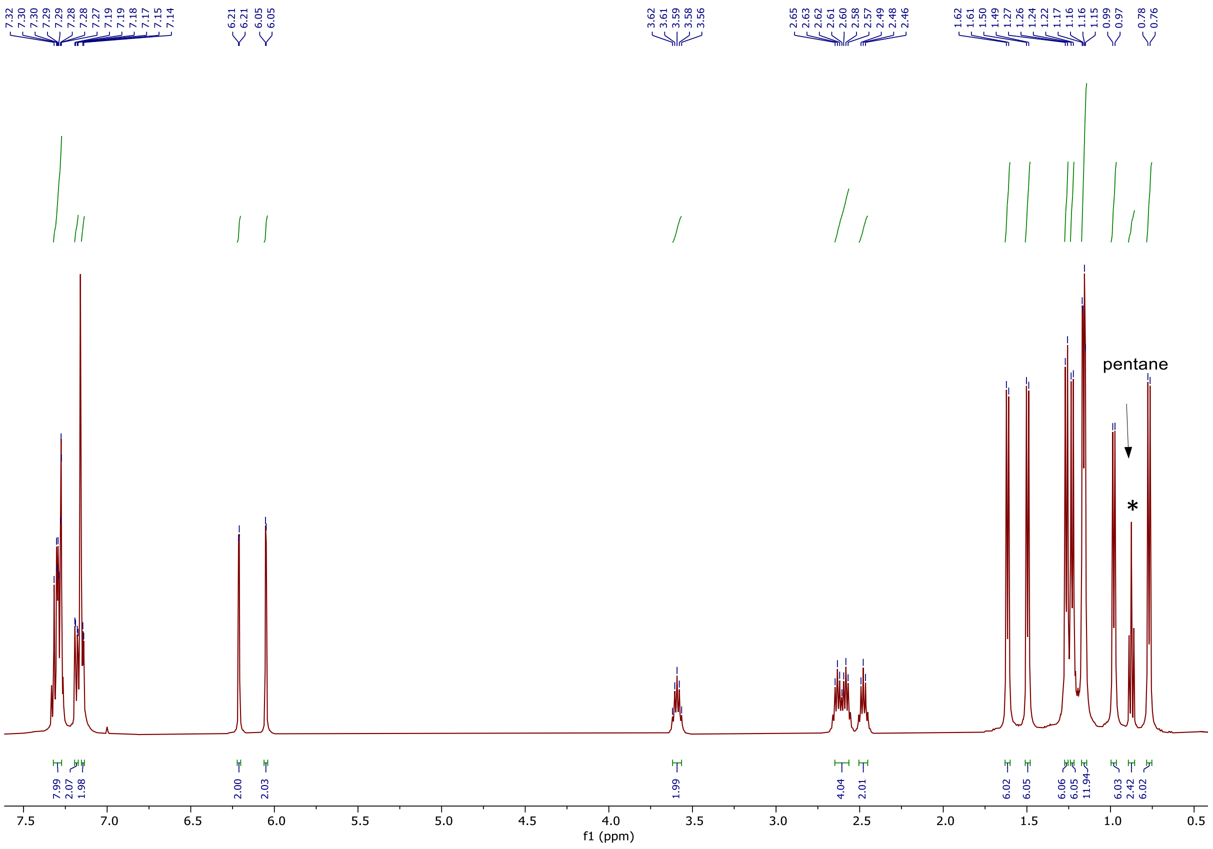

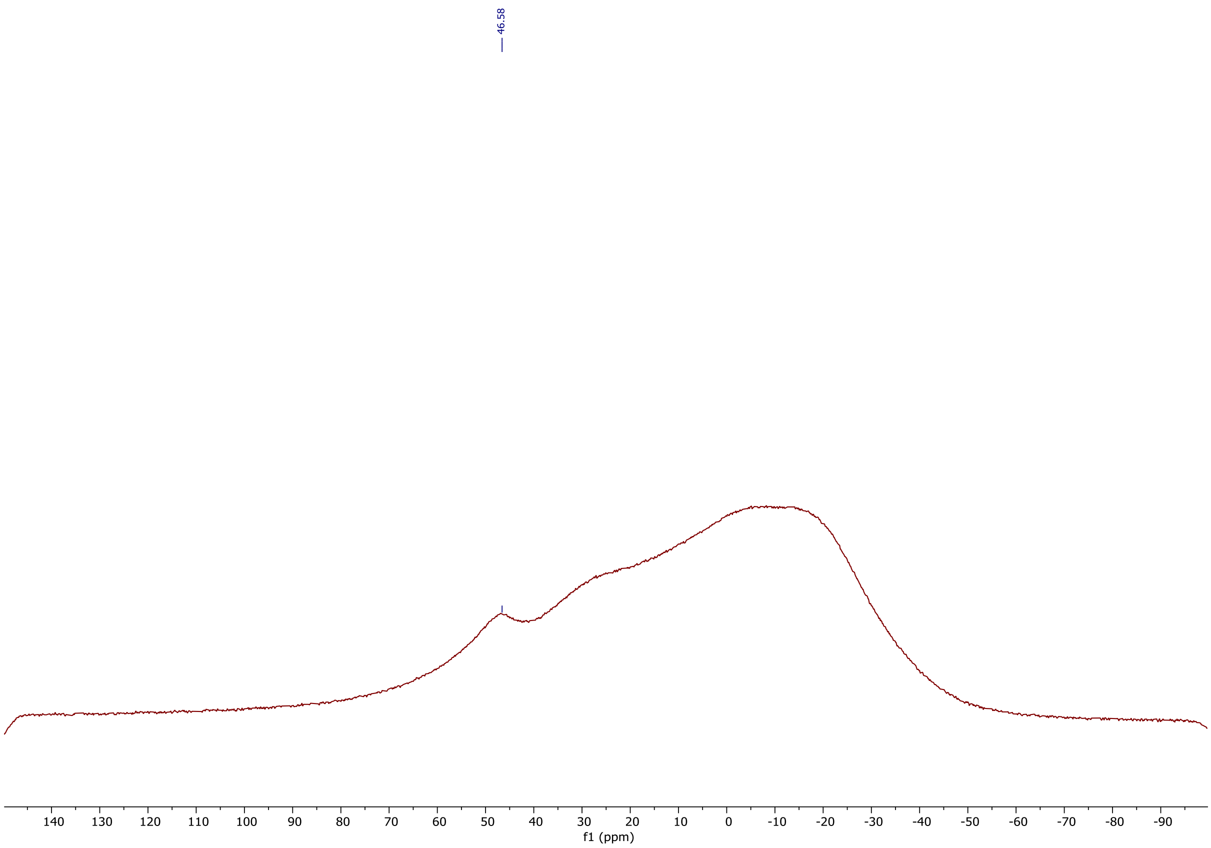

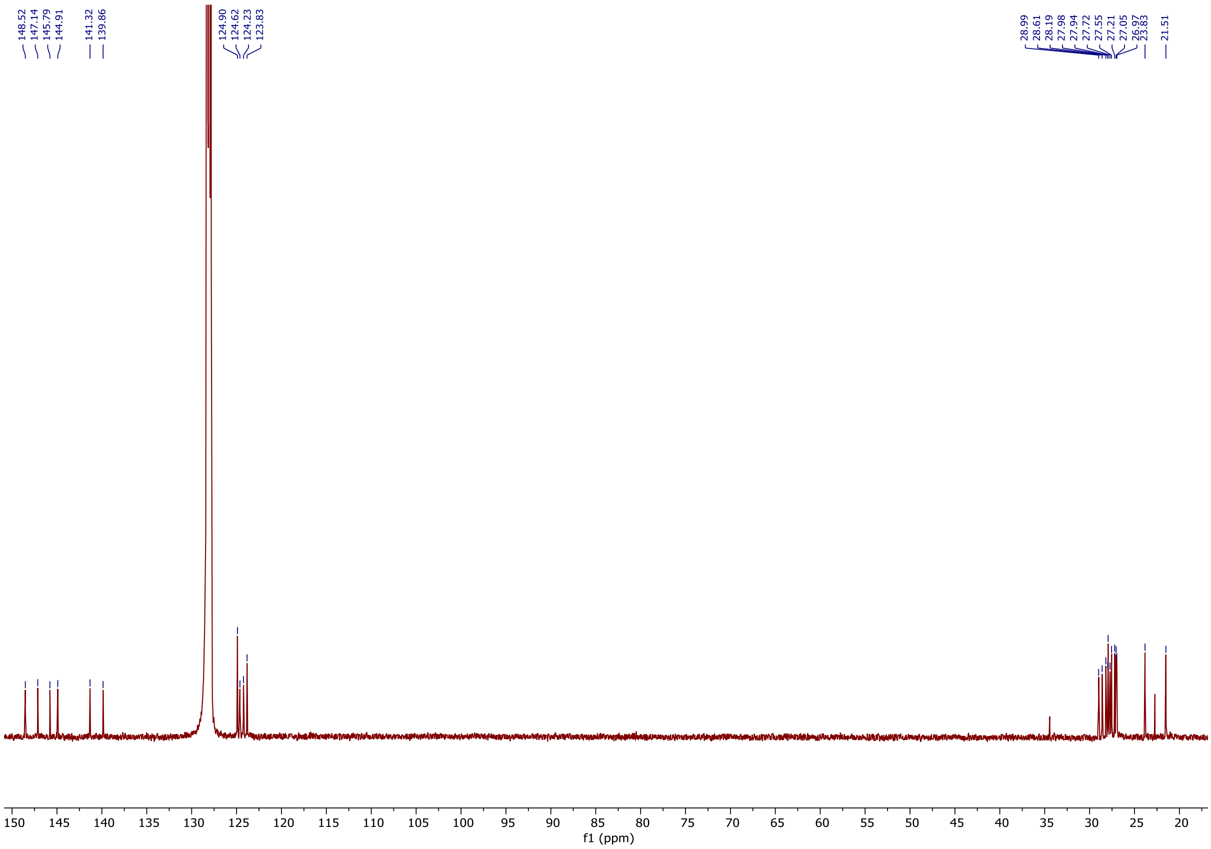

Figure s4: ^{1}H, $^{11}B\{^{1}H\}$ and $^{13}C\{^{1}H\}$ NMR spectra of **4** in C_6D_6. [* denotes minor pentane impurity]

$Ge_3\{B(NDippCH)_2\}_2(IPrMe)$, 5. To a solution of $K_2[Ge_2\{B(NDippCH)_2\}_2]$ (0.02 g, 0.02 mmol) in toluene at -35 °C was slowly added a solution of (IPrMe)$GeCl_2$ (0.006 g, 0.02 mmol) also in toluene. After stirring for 10 min, the reaction mixture was slowly warmed to room temperature and stirred for another 20 min. Volatiles were removed under vacuum and the residue was extracted with pentane. The filtrate was then dried under vacuum and used for spectroscopic characterization. Yield: 0.015 g, 63.8 %.

^1H NMR (400 MHz, C_6D_6, 298 K) δ 0.74 (s, 6H, $CH(CH_3)_2$ of carbene), 1.16, 1.22 and 1.24 (overlapping, 24H, $CH(CH_3)_2$ of Dipp), 1.44 (CCH_3 of carbene), 3.58 (br, 4H, $CH(CH_3)_2$ of Dipp), 6.06 (br, 1H, $CH(CH_3)_2$ of carbene), 6.33 (s, 2H, C*H* of boryl), 7.01 (m, 4H, *m*-Ar*H* of Dipp), 7.11 (m, 2H, *p*-Ar*H* of Dipp).

$^{11}B\{^1H\}$ NMR (128 MHz, C_6D_6, 298 K): δ_B 36.5 ppm

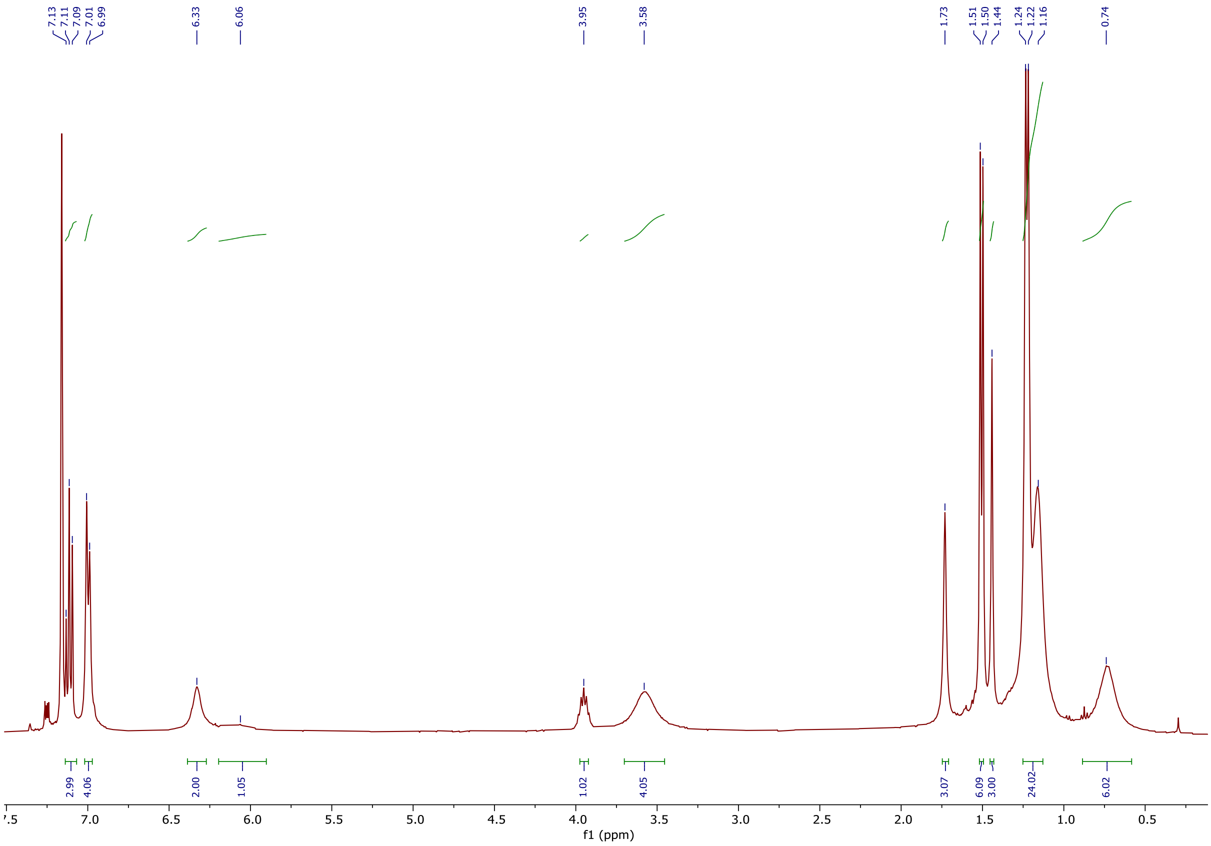

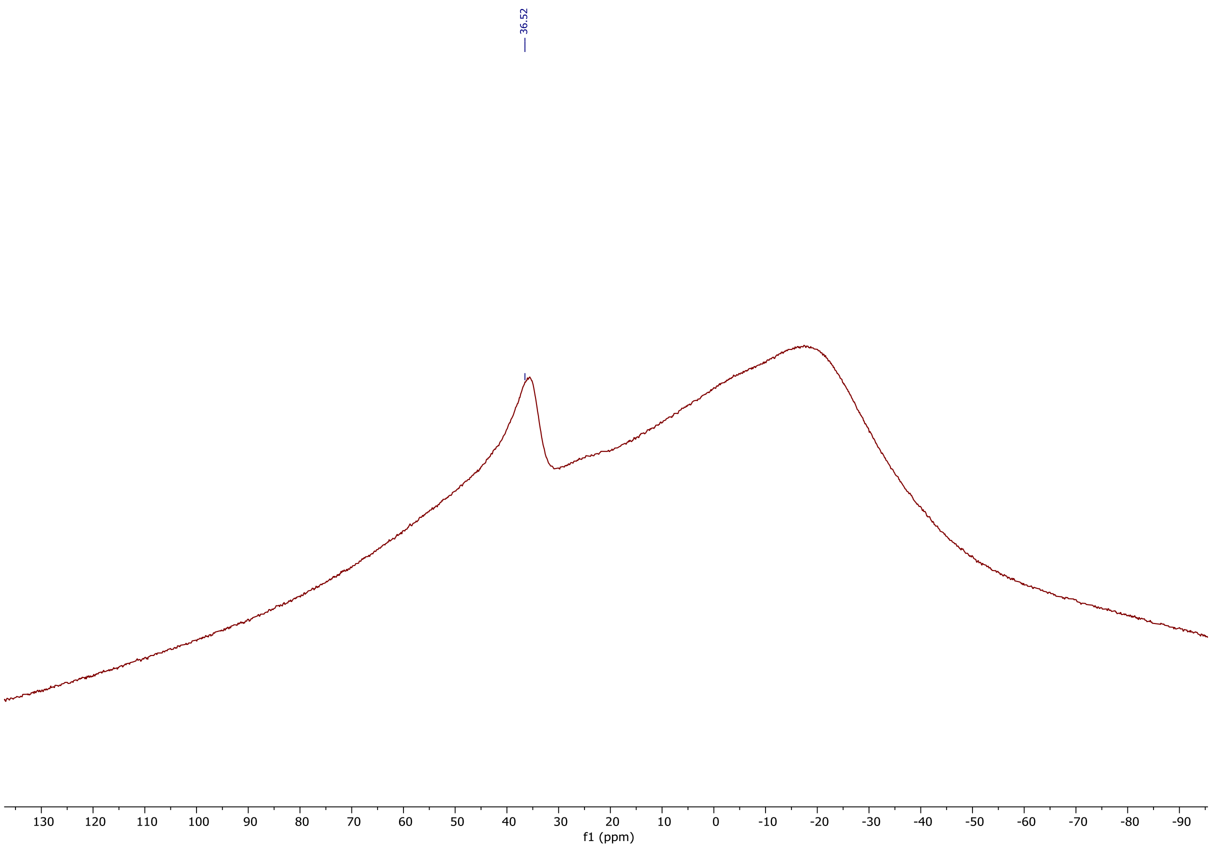

Figure s5: ^{1}H and $^{11}B\{^{1}H\}$ NMR spectra of **5** in C_6D_6.

$Ge_6\{B(NDippCH)_2\}_4$, 6. A mixture of **5** (15 mg, 0.013 mmol) and BPh_3 (3.2 mg, 0.013 mmol) was dissolved in C_6D_6 (0.3 mL) at room temperature. After standing for 2 min, the reaction mixture was filtered into a NMR tube and concentrated (to ca. 0.1 mL). 0.5 mL of pentane was then added on top of the filtrate and the mixture left for crystallization overnight. The solution was then decanted and the resulting deep green crystals were washed with pentane and dried under vacuum. Yield: 7.1 mg, 55.0 %.

Anal. Calc. for $C_{52}H_{72}B_2N_4Ge_3$: C 62.92 %, H 7.31 %, N 5.64 %; Meas.: C 62.34 %, H 7.14 %, N 5.42 %.

^1H NMR (400 MHz, C_6D_6, 298K): δ_H 0.81 (d, J_{HH} = 6.8 Hz, 6H, CH(CH_3)$_2$ of Dipp), 0.93 (d, J_{HH} = 6.8 Hz, 6H, CH(CH_3)$_2$ of Dipp), 1.06 (d, J_{HH} = 6.8 Hz, 6H, CH(CH_3)$_2$ of Dipp), 1.20 (m, 18H, CH(CH_3)$_2$ of Dipp overlapping), 1.33 (d, J_{HH} = 6.8 Hz, 6H, CH(CH_3)$_2$ of Dipp), 1.57 (d, J_{HH} = 6.8 Hz, 6H, CH(CH_3)$_2$ of Dipp), 2.41 (sept, J_{HH} = 6.8 Hz, 2H, CH(CH$_3$)$_2$ of Dipp), 2.53, 2.57 (m, 4H, CH(CH$_3$)$_2$ of Dipp overlapping), 3.57 (sept, J_{HH} = 6.8 Hz, 2H, CH(CH$_3$)$_2$ of Dipp), 5.83, 5.91 (d, J_{HH} = 2.3 Hz, 4H, CH of boryl), 7.11 (m, 2H, ArH of Dipp), 7.17 (m, 4H, ArH of Dipp overlapping with C_6D_6), 7.30 (m, 6H, ArH of Dipp).

$^{11}B\{^1H\}$ NMR (128 MHz, C_6D_6, 25 °C): δ_B 33.3.

$^{13}C\{^1H\}$ (151 MHz, C_6D_6, 298 K): δ_C 22.6, 23.8, 27.1, 27.3 and 27.5 (CH($\underline{C}$H$_3$)$_2$ of Dipp), 27.9, 28.1, 28.8 and 29.2 ($\underline{C}$H(CH$_3$)$_2$ of Dipp), 123.5, 123.8 ($\underline{C}$H of boryl), 124.0, 124.9 (*p*-Ar of Dipp), 125.0, 125.1 (*m*-Ar of Dipp), 139.9, 141.7, 145.0, 145.5 (*o*-Ar of Dipp), 147.2, 148.7 (*i*-Ar of Dipp).

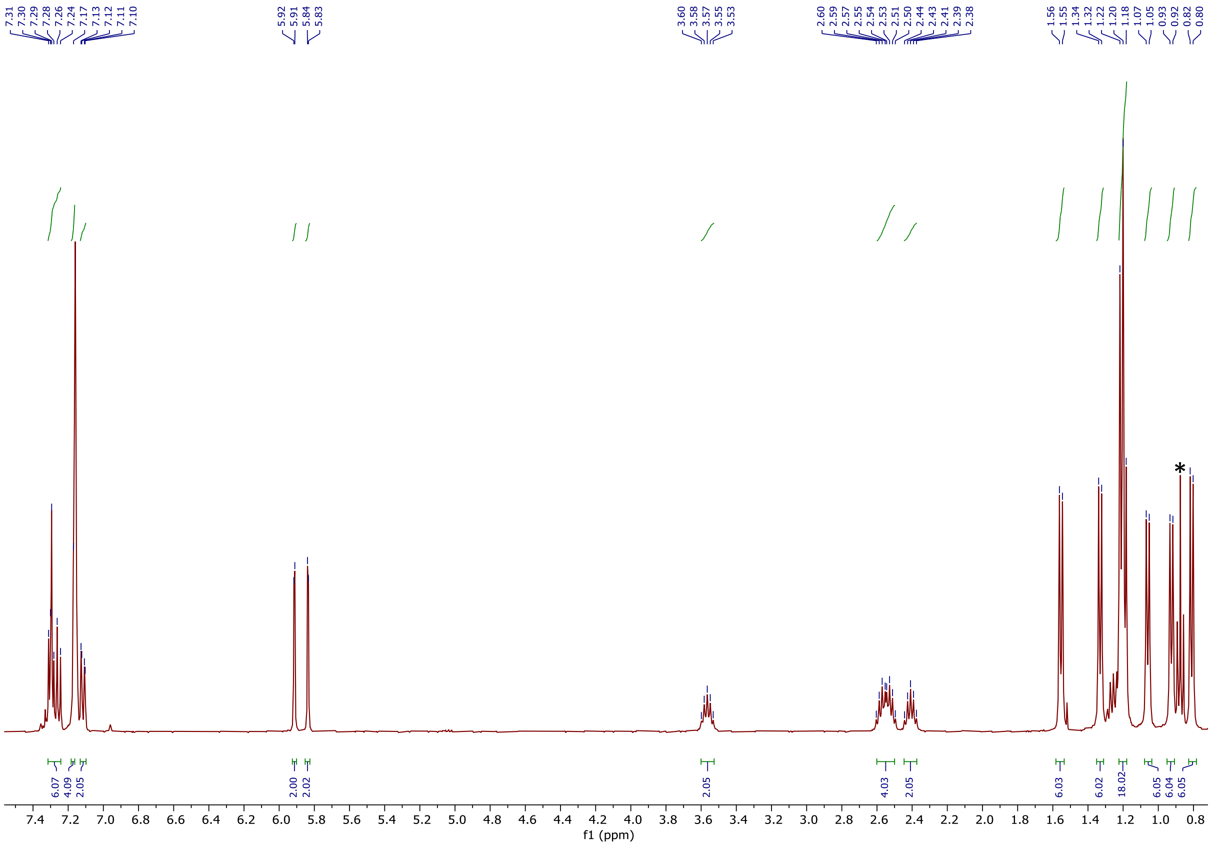

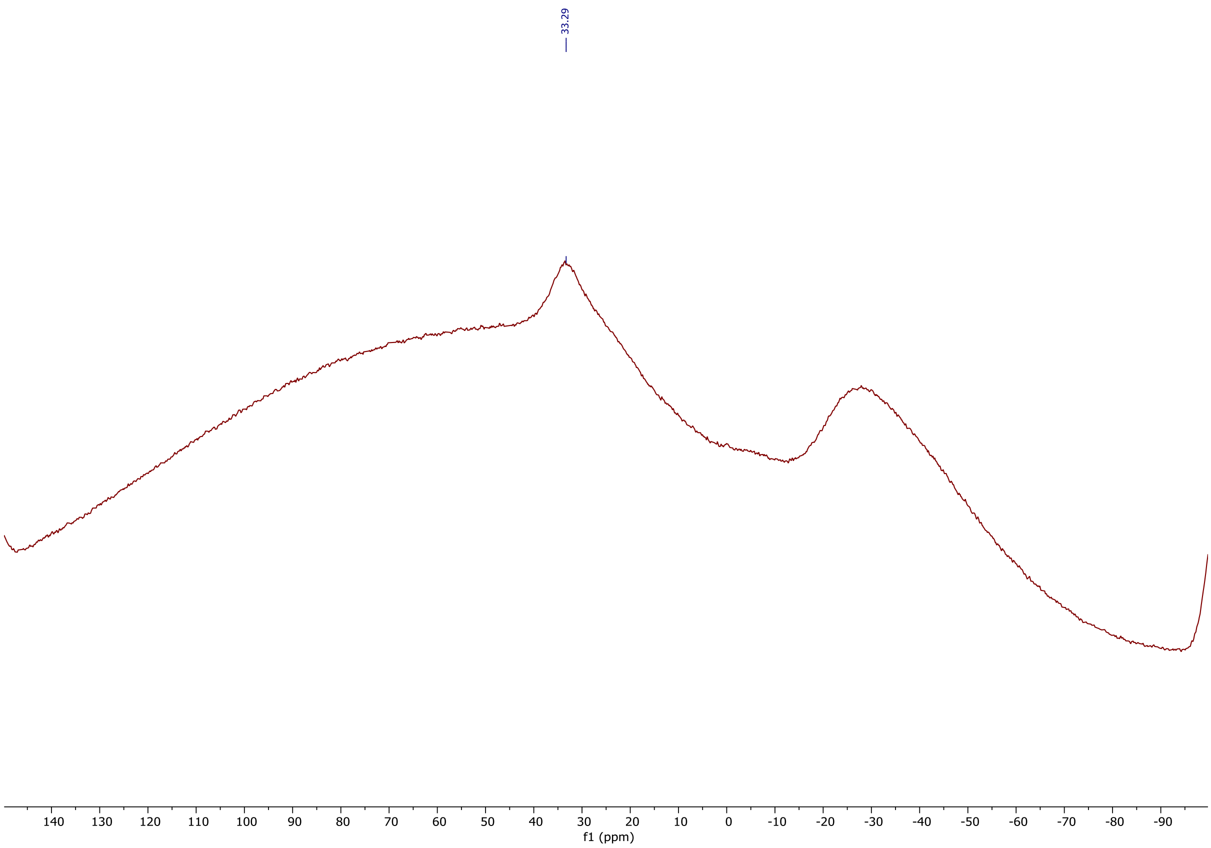

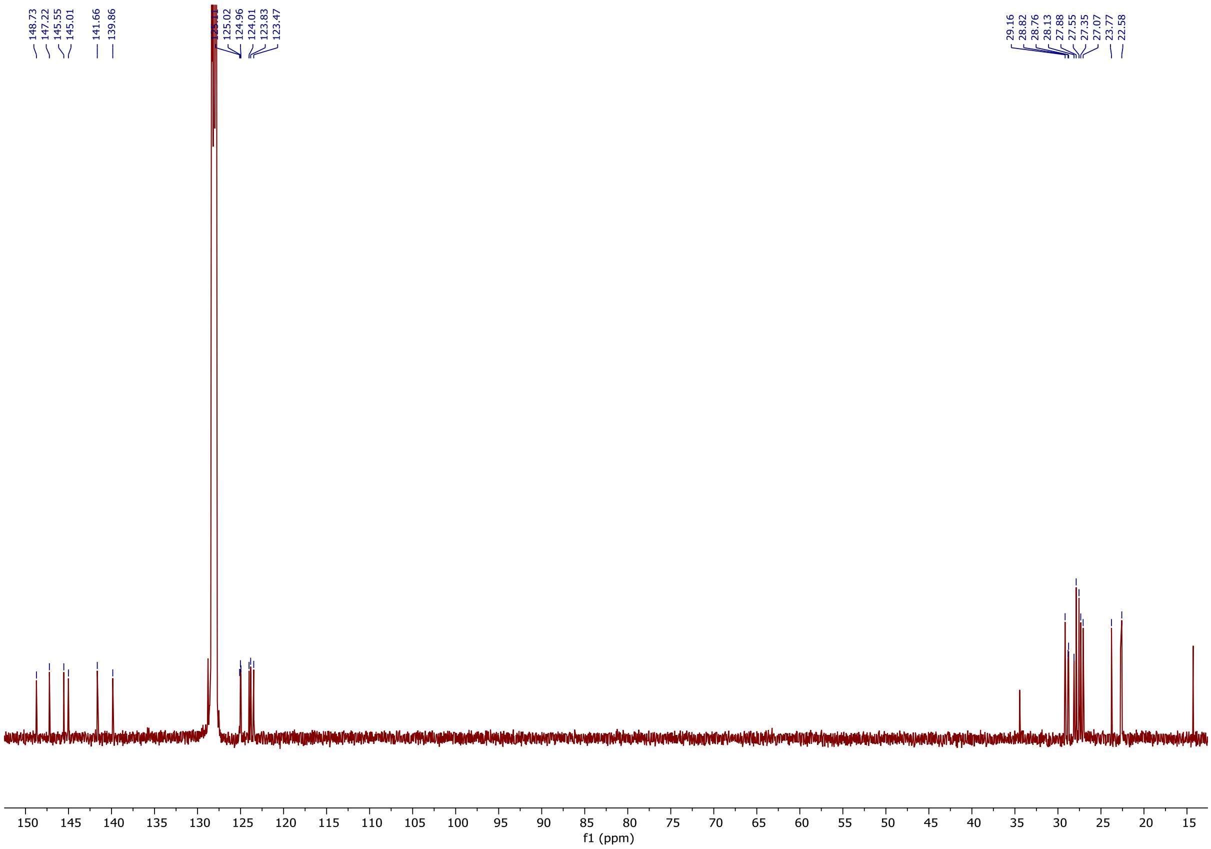

Figure s6: ^{1}H, $^{11}B\{^{1}H\}$ and $^{13}C\{^{1}H\}$ NMR spectra of **6** in C_6D_6. [* denotes minor pentane impurity]

2. Details of DFT calculations and *xyz* files

All computational work reported here was carried out at the density functional theory (DFT) level, using ORCA (Revision 5.0.1).[s1-s3] The exchange correlation functional B3LYP[s4-s7] was employed in conjunction with the LanL2DZ basis set with the D4 dispersion correction. [s8-s11] All calculations were performed on model systems to reduce the computational cost, with ^{i}Pr groups being replaced with Me groups. The nature of the stationary points, minima and transition states, was confirmed by full frequency calculations, and are characterized by zero or one imaginary frequency respectively along with full IRC (intrinsic reaction coordinate) calculations for transition states.

Xyz coordinates:

```
I1
168
  Sn  -1.03803953580506     -2.40621341634987      0.50023490190938
  Sn  -3.58322081789366     -1.49815079536887      0.15691764722316
  B   -4.90974931278622     -1.97105183856607      1.86232833135492
  B   -4.06528656059538     -0.31193098983444     -1.65125411882302
  N   -4.35080378185945     -2.26013487340133      3.17065409303393
  N   -6.31666736793239     -2.27117274472862      2.01295885853777
  N   -4.94911432526692      0.78926843488650     -1.96012946369690
  N   -3.22398893760121     -0.42041569527493     -2.82826967945983
  C   -2.97261762955888     -2.24875383978210      3.56383830463701
  C   -5.37827986057747     -2.70837826961090      4.03284107163490
  C   -6.55819116995612     -2.70818962182053      3.33975916425386
  C   -7.39058993949807     -2.21633509005157      1.06083594160649
  C   -5.94360798346615      1.44795843363465     -1.16044260586715
  C   -4.63653834540133      1.28487098924131     -3.25206425730189
  C   -3.59299172857903      0.56451074970657     -3.76923271934912
  C   -2.04950870132079     -1.23436407042644     -2.92626275412329
  C   -2.37726422294661     -1.04309287859447      4.00604381172654
  C   -2.23777652071327     -3.46030181756245      3.49798045176368
  H   -5.18710501362010     -2.98293407402794      5.05954479566321
  H   -7.54649999026716     -2.98883226434707      3.67280550439064
  C   -8.32807516312876     -1.16141199406792      1.15018207469363
  C   -7.52215158776014     -3.24794640539295      0.10158011616393
  C   -5.53114917910816      2.35635371481050     -0.15641006317581
  C   -7.30988763961772      1.23012043218810     -1.45066527124551
  H   -5.17911196451203      2.11151537441365     -3.68657016090487
  H   -3.09517647229272      0.66870794056532     -4.72190779307733
  C   -0.84916466451571     -0.72750421904290     -2.36462525599636
  C   -2.12955469123162     -2.53208115005737     -3.47221011401920
  C   -3.15939651916082      0.25051845306002      3.99232851445651
  C   -1.03072058255276     -1.07105513643832      4.41604110337215
  C   -0.88303099764984     -3.44487360400941      3.89785999971363
  C   -2.90351607311489     -4.74540352699209      3.04775731113679
  C   -8.13919482040087     -0.05181991136730      2.16105294665043
  C   -9.42773468813661     -1.16428371085095      0.27067289899735
  C   -8.62623873652015     -3.21002911105779     -0.77456239630733
  C   -6.51777014900085     -4.37904169557673      0.03166792380339
  C   -6.52366720754237      3.06534435167137      0.55058900597176
  C   -4.06296856838012      2.58268363919926      0.14031889571344
  C   -8.27333676306383      1.96694209465939     -0.73522995343355
  C   -7.71808422344124      0.21721049910493     -2.49784221549444
  C   -0.77715124107707      0.69980982788456     -1.86515866332175
  C    0.27343497024839     -1.58309960390877     -2.30772594677298
  C   -0.97353751740925     -3.34062543619221     -3.45123867292544
  C   -3.44482854458689     -3.06169689375668     -3.99624011671072
  H   -2.65770302322769      1.02172427179500      4.58756358318009
  H   -4.17697579578988      0.11280141751424      4.37436849748840
  H   -3.25539113914362      0.62174957586881      2.96137538954841
  H   -0.55744115769302     -0.15480674028973      4.75929478754795
  C   -0.28673480813724     -2.26160317233641      4.36378328503456
  H   -0.30409151271283     -4.36368445598349      3.84684423800556
  H   -3.61102726447252     -5.10767013568361      3.80620668441276
  H   -3.47551433526964     -4.59719805081054      2.12531671062119
  H   -2.15932513898377     -5.52968267176756      2.87218063239146
  H   -7.20689376689345      0.49295091939697      1.96782141872908
  H   -8.96297960654608      0.66683151308161      2.11200350832971
  H   -8.07646021418086     -0.43984698507135      3.18508938751031
  H   -10.16140146245019    -0.36456173626260      0.33766756929696
  C   -9.57965632112040     -2.18180893604729     -0.68519489563653
  H   -8.74407141703051     -3.99881446230936     -1.51447340663504
  H   -6.31289931244669     -4.79077578223987      1.02765572005671
  H   -5.55391833141809     -4.03388698363699     -0.36743666903706
  H   -6.88333959012362     -5.18711678730293     -0.61207813778553
  H   -6.22399588525853      3.77366594039773      1.31999160670091
  C   -7.88535012329165      2.88287210415442      0.25608033110766
  H   -3.92279704292077      3.47147226968615      0.76579050519080
  H   -3.62501347670244      1.72283132532176      0.66738814043919
  H   -3.48192717975732      2.71058073372474     -0.78074687664874
  H   -9.32700783861581      1.81701166203627     -0.95794347683412
  H   -8.80746829045427      0.16607111837014     -2.58906220000413
  H   -7.29777246199676      0.45728035765098     -3.48256360155449
  H   -7.35529296173200     -0.78243673889897     -2.22942373602029
  H   -1.49823495919165      0.87925236318589     -1.05994338534309
  H    0.22162058905799      0.93758124191207     -1.48844201217307
  H   -1.01827707451617      1.40550230611893     -2.67065877711375
  H    1.19528306622075     -1.21697675798366     -1.86559525349508
  C    0.21154806783668     -2.88082265586622     -2.85609231837624
  H   -1.01916218215963     -4.34435734010091     -3.86709701911902
  H   -4.12801475034480     -3.26903707720192     -3.15976166908982
  H   -3.94965073568854     -2.33340687508632     -4.64113556427630
  H   -3.30139533392952     -3.99130527244569     -4.55820209167519
  H    0.75486644530472     -2.26124424961954      4.67316978743568
  H   -10.43455829053842    -2.17394486587487     -1.35765797852098
  H   -8.63907958079740      3.44829136268563      0.79955263512421
  H    1.08464657763586     -3.52668239542632     -2.80511741540236
  Sn   2.75929991007556      3.50969421975308     -0.04939127257358
  Sn   3.09289176827125      0.81053352647564     -0.13719971125270
  B    3.60935132506015      4.56477985992335     -1.79666909223876
  B    1.64058763582508      4.39714931734041      1.64358971305849
  N    3.82787548298025      3.86061657375454     -3.04638348633576
  N    4.23812702172451      5.84998648339905     -2.00838448275661
  N    0.76219304538962      5.53168618902557      1.82327407775654
  N    1.49393992473838      3.64725083044621      2.87625965721385
  C    3.50418740130433      2.49985372628271     -3.35690646086356
  C    4.54716012277672      4.69165680386587     -3.93597398575639
  C    4.78736129032761      5.88712242205360     -3.31502847755457
  C    4.39724654312570      6.96906071817108     -1.12219270444905
  C    0.40901773058833      6.59738843216889      0.92779290871373
  C    0.14381579979978      5.43954785980935      3.09655203370500
  C    0.57587978526009      4.30209489173890      3.72489938412828
  C    2.02646941452007      2.33972886203768      3.11650389628487
  C    2.21899181622031      2.18367862670717     -3.85880106414085
  C    4.48763159380056      1.49998133630016     -3.14767610439855
  H    4.82372435844845      4.36797019373033     -4.92832496184082
  H    5.31060592171082      6.75616473413668     -3.68565244053172
  C    3.62436410264600      8.13257674828756     -1.34424224142885
  C    5.36404467906886      6.90603542631888     -0.09073189695514
  C   -0.50216180893753      6.34408970076958     -0.12506623942210
  C    0.92694465205362      7.89100919141397      1.16731632098757
  H   -0.55505511072235      6.18752160094517      3.44097797497723
  H    0.31084098639692      3.91587278375865      4.69789286619026
  C    1.29379491786731      1.22539088104474      2.63230373595950
  C    3.28761532202803      2.19452222980237      3.73171335028601
  C    1.16887362513237      3.26074102720426     -4.01294262464031
  C    1.93441315660445      0.84230764649000     -4.17567664434606
  C    4.15752821352652      0.16080012193193     -3.45577414981982
  C    5.87358054184687      1.86536126447854     -2.65378193722098
  C    2.57122993226315      8.16061497928804     -2.43017782231241
  C    3.84937672086901      9.25511255254680     -0.52436222753754
  C    5.55126499891084      8.04329015422529      0.72137126043558
  C    6.19743322139186      5.66016811194595      0.12417234485075
  C   -0.90291519348312      7.42538956118741     -0.93623060710712
  C   -1.05504281101808      4.95436989594823     -0.36589114162630
  C    0.49422470765248      8.94981964149567      0.34608038996236
  C    1.93337876838376      8.12042466578203      2.27381283487068
  C   -0.08944914728060      1.40966017097651      2.04595947462845
  C    1.88125587017767     -0.05552457793158      2.73648252752093
  C    3.82058685238659      0.89599412531885      3.87053965038116
  C    4.07335256365699      3.41283659426721      4.16074745543221
  H    0.31392711485460      2.89591673215936     -4.59299964485345
  H    1.57383291667637      4.15398026442687     -4.50213333862271
  H    0.80200110512194      3.57847080030633     -3.02646423531430
  H    0.95295730934276      0.58091075273595     -4.56257936446593
  C    2.89441659994609     -0.16407027047624     -3.97637025589592
  H    4.89827505226758     -0.61843677676915     -3.29291319030123
  H    6.46565205565521      2.32461749417589     -3.45790193031279
  H    5.83006244395890      2.59121259977109     -1.83533238938027
  H    6.40988913198699      0.97703223583385     -2.30288799436368
  H    1.80085378150985      7.40324305429359     -2.24099387027111
  H    2.07811822839195      9.13674755748893     -2.47088279882199
  H    2.99683463455182      7.94498747368024     -3.41778653366394
  H    3.26805544988350     10.15829427415178     -0.69331797764389
  C    4.80712200625247      9.21456156234942      0.50170770268670
  H    6.29528727750003      8.01165609073176      1.51446070018296
  H    6.59493378456713      5.27925256596286     -0.82454479895647
  H    5.59914702965082      4.84761526459473      0.55950759149787
  H    7.03713082655542      5.86307359720097      0.79864312680477
  H   -1.60881574750387      7.24896160726213     -1.74498931647256
  C   -0.41808514719963      8.72224691358636     -0.69694052583525
  H   -1.90525140344087      4.98613340923508     -1.05640805177770
  H   -0.29147628768271      4.29059819098274     -0.79580261734783
  H   -1.38325116573199      4.48569024855026      0.56945439620523
  H    0.87780095115733      9.95099997004697      0.52721935561042
  H    2.24686793780567      9.16848526908821      2.30622455869725
  H    1.52776612953562      7.84858690392944      3.25647801855638
  H    2.82772643220362      7.50570497674364      2.11520911280023
  H   -0.09177912358103      2.17000093835346      1.25809211186689
  H   -0.46911159073190      0.47358386401657      1.62635451036388
  H   -0.79617908783494      1.74562191281933      2.81647168111507
  H    1.34203784750318     -0.91871479181317      2.35737330394551
  C    3.13480681402021     -0.21790811271297      3.36090052849373
  H    4.79186121851725      0.76792152188909      4.34242834663582
  H    4.46515912820607      3.93786292786438      3.27726983047472
  H    3.44763654300375      4.13166984466915      4.70168982183676
  H    4.92175562603649      3.13393570358713      4.79559188574397
  H    2.64969723376173     -1.19537314252466     -4.21478403096552
  H    4.97233102622133     10.08889050965293      1.12727927744745
  H   -0.74718566757362      9.54909065661334     -1.32233628679601
  H    3.57176632034736     -1.21037431029753      3.43806383105030
```

```
I2
168
  Sn  -1.25586050179112     -1.07331955690446      1.59541171026651
  Sn  -4.09616451427005     -0.93304027219197      0.74991650043259
  B   -5.42266053935483     -2.43763982243161      1.70449147958183
  B   -4.37639835605023      0.01314589823730     -1.24397498032522
  N   -5.27147918644921     -2.68713042200896      3.12676397468581
  N   -6.38491345969708     -3.43109727238437      1.28620771992776
  N   -5.50526516987647      0.65093905847783     -1.88470728546826
```

```
N   -3.26552438304027     0.30959412090012    -2.12715358341909
C   -4.26869312884958    -2.02739363859450     3.90225446205529
C   -6.09186910098174    -3.76435552583581     3.51950039268153
C   -6.76900026045508    -4.20692430956064     2.41511354816676
C   -6.91142502695832    -3.74853377439737    -0.01123270781083
C   -6.89848908326708     0.62003367786255    -1.53613302950971
C   -5.06794320960208     1.28277032533628    -3.07500408449809
C   -3.72168757292381     1.08287625871044    -3.21895591383470
C   -1.91822592387100    -0.15011932710961    -1.97006289513510
C   -4.53386727335371    -0.72856765220911     4.39832008334205
C   -2.97611492910881    -2.60491614476601     3.96369159630204
H   -6.13298491805967    -4.12391519825872     4.53683569366799
H   -7.48444514387225    -5.01126085905704     2.32889693138804
C   -8.25398386443445    -3.41603046413656    -0.30703369226226
C   -6.10074626386082    -4.45329572912360    -0.93250074983080
C   -7.40076147493755     1.50797703432613    -0.55791957345116
C   -7.73996540895795    -0.29023560106347    -2.21905218331047
H   -5.74998494146136     1.82656831660780    -3.71182222377911
H   -3.06117078563230     1.43609986429892    -3.99382158543489
C   -1.00255579437232     0.62611639920060    -1.22274714087841
C   -1.56323602635803    -1.41072498671812    -2.50049266161679
C   -5.94402966704130    -0.18207159591669     4.42913854121621
C   -3.45092552019411     0.02998312855201     4.91699829440404
C   -1.91173736277424    -1.82932676928343     4.48381920654125
C   -2.74148612504833    -4.02258723283923     3.48837986524062
C   -9.07993451756803    -2.61319465336080     0.67485869920388
C   -8.79418107527186    -3.83752492968094    -1.53760081556234
C   -6.66847875178591    -4.84241870309380    -2.16296716473594
C   -4.66827344016144    -4.80954974111648    -0.59375403392822
C   -8.78031942525804     1.46640057155483    -0.26543062905753
C   -6.49020291560571     2.48811904658375     0.14916972210470
C   -9.11410596311591    -0.29248222798282    -1.91410429658933
C   -7.16378307261961    -1.24814114691125    -3.23908603330414
C   -1.35378116929968     2.02997184330711    -0.78278794990982
C    0.27827131750130     0.08926589221412    -0.96419557199521
C   -0.26351495323657    -1.90015950875317    -2.26169075228973
C   -2.58154265280024    -2.23789633673787    -3.25406478344264
H   -5.94331525374547     0.89546715915711     4.62524503902307
H   -6.52760417149006    -0.67549404120158     5.22003159470283
H   -6.46063192703427    -0.36220401759810     3.48259159163239
H   -3.64376790664241     0.99371613619342     5.37979119093939
C   -2.14274564172264    -0.50995729477271     4.93084174376680
H   -0.92212816942511    -2.26653033716636     4.57660173605026
H   -3.21253205955640    -4.74495625129943     4.16952005911671
H   -3.17699730700195    -4.18520370650167     2.49710932656119
H   -1.67089627033735    -4.24616712528082     3.43675749498633
H   -8.60945896430303    -1.64169237056944     0.87014877571225
H   -10.08384157738577   -2.42613692256962     0.28014551455207
H   -9.17609462033367    -3.12299919145066     1.64131943671268
H   -9.83010265813986    -3.60154665100607    -1.77033025090294
C   -8.01091629203373    -4.55172946267002    -2.45893726195374
H   -6.06001196588220    -5.39114484488323    -2.87863256799801
H   -4.60771412131090    -5.32264926981153     0.37476768178893
H   -4.03665569119303    -3.91493937026465    -0.51124923850450
H   -4.23609780511945    -5.46317514473555    -1.35996063557366
H   -9.18376719337119     2.14508279593648     0.48305740754355
C   -9.63316568686671     0.57772374605571    -0.94096256776642
H   -7.07117238770393     3.26630031261318     0.65756411606726
H   -5.87057827667385     1.98173884732878     0.90242584752616
H   -5.79926008768798     2.96972721448064    -0.55347291010494
H   -9.77107321136715    -0.98499974136109    -2.43465535639118
H   -7.95436707398550    -1.83139956485407    -3.71961111072563
H   -6.59517579564484    -0.72249388158487    -4.01558018972284
H   -6.47375147980483    -1.95368091252779    -2.75988027213516
H   -2.42426160688545     2.13729716795039    -0.57469314596716
H   -0.78291871780343     2.32400928989580     0.10339341622530
H   -1.11601600877751     2.73759170527967    -1.58856188011235
H    0.99820128967719     0.67718277894137    -0.40130946787414
C    0.64529641066533    -1.16496469365345    -1.48439109147133
H    0.01949589894282    -2.87278251854303    -2.65786491979357
H   -3.34722062401813    -2.62545447507221    -2.56740561620895
H   -3.11143974470060    -1.64417047591678    -4.00813806759246
H   -2.10626628153865    -3.09177364767140    -3.75028521230451
H   -1.32165198615136     0.08083935074793     5.32611471690607
H   -8.44049888658081    -4.87283088965856    -3.40527987226316
H   -10.69650323230542    0.56538110772074    -0.71170917983239
H    1.63632390677564    -1.56068282672278    -1.27753405497708
Sn   2.56650112209074     3.26336598703789     0.02309778795915
Sn  -3.07567594444590     1.31396326913625     2.38396893492537
B    3.29644670571130     4.40091049440090    -1.79951335972279
B    2.51587051825920     4.78146431783097     1.70737961940643
N    2.87008074656284     4.07023747925990    -3.14753699172378
N    4.45171533925106     5.26245298193126    -2.01499645059322
N    1.98238974185837     6.12943378743094     1.83376341759852
N    2.74575006351289     4.38235601288186     3.08344556788926
C    1.80139680711898     3.20712916021624    -3.57166206471887
C    3.71754993504214     4.70099669254815    -4.08568867463669
C    4.67095767199663     5.40891905336002    -3.40739035855496
C    5.29663388002548     5.92329763481379    -1.06437920972220
C    1.57157853707895     7.04965999523218     0.81524605698451
C    1.90917430803333     6.47426492856698     3.20627939019945
C    2.37572604006948     5.42773731748551     3.95533078122201
C    3.23837556422518     3.11473307811692     3.54737878463921
C    0.54051334420298     3.76952169001475    -3.88092541839286
C    2.04424479830742     1.81907882390514    -3.70442084405667
H    3.58204865295825     4.58508885205013    -5.15080048680033
H    5.49608464749159     5.98368857762664    -3.79950458852107
C    5.39448347090216     7.33916238044359    -1.09841349367308
C    6.04473051384130     5.16495461998714    -0.12899496654960
C    0.50817528073151     6.71014553829022    -0.05766840078434
C    2.22680568048698     8.30382542487313     0.71397858540645
H    1.50984948086952     7.42077486272292     3.53751914888156
H    2.45659451554837     5.33952672384891     5.02856122451708
C    2.33611953694814     2.03549624230053     3.69769720960616
C    4.62088416677959     2.96980713508813     3.81337442045120
C    0.31359660613054     5.26159830112355    -3.76249216910899
C   -0.48681525317423     2.91422203666353    -4.32743499953161
C    1.00136094133865     0.99677028902579    -4.17139393691238
C    3.38560622453905     1.22529300727164    -3.33541836913595
C    4.56087565153193     8.16914842180486    -2.05439114832066
C    6.27344744456949     7.97861831847741    -0.20418789258695
C    6.90619590618590     5.84428959169340     0.75696940550783
C    5.96430000631685     3.65357349334598    -0.06170847587231
C    0.09618831547877     7.65463717131394    -1.01986040344071
C   -0.20385836963655     5.37633895396145     0.02197864268375
C    1.78020157950273     9.22342868230614    -0.25389626644429
C    3.40316157143698     8.65564214954032     1.60258677563213
C    0.86170973399465     2.19396840282541     3.40314994411179
C    2.84910210289003     0.78573205560792     4.10187137330032
C    5.09967645266725     1.70681916661758     4.21320795681539
C    5.55667533590047     4.15338221794558     3.68688192107663
H   -0.75702847778892     5.49775600516318    -3.77333362866168
H    0.78379707747331     5.80012408214473    -4.59752010245519
H    0.75448018949373     5.66680789568810    -2.84727910644784
H   -1.46152975818190     3.33667915721419    -4.56315718860446
C   -0.25605047403494     1.53680357236532    -4.47945163036968
H    1.17213930777260    -0.07024347846462    -4.27985044420998
H    4.21659469025049     1.82135893646195    -3.73045132902722
H    3.50088679468659     1.19482911598805    -2.24211448350168
H    3.47731777842799     0.20080485406881    -3.71269029027000
H    3.55616161874637     7.75165158216814    -2.17085568392747
H    4.46405232633554     9.19484355392524    -1.68299356338269
H    5.01148890523164     8.22088393128776    -3.05460360781243
H    6.35306764969402     9.06348543516234    -0.22782344523202
C    7.03052606191261     7.24107910660434     0.71943733795779
H    7.49624557057933     5.26729906910961     1.46551570905157
H    5.73623788957519     3.21091521096136    -1.03573989301870
H    5.19444984527011     3.31964443115651     0.65755781323201
H    6.90790163099743     3.23004298430418     0.30204595660002
H   -0.73154522552679     7.40781270524610    -1.68086528466399
C    0.71906837143436     8.90826771008780    -1.11726156483699
H   -1.22679451062708     5.45457423534496    -0.36451931541257
H    0.29563329691787     4.61588656718029    -0.60328646346039
H   -0.24648323619078     4.99227279154554     1.04552472599413
H    2.27727695028712    10.18797796454431    -0.33403115180157
H    3.99890003595040     9.45373236692594     1.14733392001167
H    3.08151678193554     9.00587506890766     2.59254532492543
H    4.05418519084987     7.79017609145022     1.75909491915608
H    0.65884459573656     2.01944819530505     2.33768173609860
H    0.26598365771192     1.45826138365009     3.95292405951680
H    0.49693739542842     3.19714005651343     3.64570011658904
H    2.16667923192413    -0.05528233290303     4.20676799833362
C    4.22057519320380     0.61803401218537     4.35303490785756
H    6.16109625018153     1.57901609409831     4.41567609000504
H    5.35993818390951     4.73026887495919     2.77825360693891
H    5.42983581792965     4.84800755838999     4.52945303594556
H    6.60332861357138     3.82695008593643     3.67862055944009
H   -1.04391329006261     0.88061795638283    -4.83925461771879
H    7.70675654349780     7.74837826993530     1.40327317274470
H    0.38327797766108     9.62958095490314    -1.85834364833975
H    4.60335888729627    -0.35362339352127     4.65744355428033
```

I3
170

```
Sn  -2.34759996521962     0.08130000115471     0.00000000493688
B   -3.48120011717633     1.86379998574328    -0.65990006563878
N   -2.98079988344048     2.78149999741589    -1.66179995350607
N   -4.68629996023266     2.49720000520464    -0.17779997562000
C   -3.85450005482047     3.88880001140191    -1.76690002757997
C   -4.88119999066092     3.71479999963583    -0.87599999343966
H   -3.67417318638689     4.70524923734380    -2.45034749951494
H   -5.72217106313907     4.36047089027936    -0.67043323334228
B   -3.22230001558105    -1.92310000748404     0.34170001638494
N   -2.82220000083776    -2.75929999491333     1.45419999181682
N   -4.09189999196674    -2.77389999516728    -0.43810001011355
C   -3.43009999884539    -4.03029999975898     1.33450000489094
C   -4.19590000401807    -4.03640000665309     0.19820000112415
H   -3.26450411149652    -4.82034134537286     2.05182056137515
H   -4.78973783850047    -4.83613008010579    -0.21922646303803
C   -1.97658175318358    -2.33839951498125     2.52914640642255
C   -2.56310630100528    -1.79621031711694     3.69374826252298
C   -0.57334350435964    -2.33958950453625     2.33340481471111
C   -1.71722274423503    -1.26284880884987     4.68896699884116
C    0.24433466613053    -1.74448466972833     3.32176427667907
C   -0.32809993987487    -1.21893818799986     4.50104807042896
H   -2.15586974552178    -0.84067757977147     5.58972635759312
H    1.32375899566695    -1.73961515854393     3.19057734117891
H    0.31200607433381    -0.76955911239350     5.25553103257305
C   -4.81090672274371    -2.46108958758591    -1.64107924842230
C   -4.12433307381579    -2.42179128872194    -2.87779559948248
C   -6.19770681765904    -2.19109093311978    -1.55378811704933
C   -4.85433441007021    -2.08527974854015    -4.03709743505508
C   -6.89740800042474    -1.87343935807610    -2.73397226032535
C   -6.23073755459857    -1.81318751838062    -3.96963871966914
H   -7.96330418640470    -1.66277137613992    -2.68001810229083
H   -6.78115654895229    -1.56079521138340    -4.87320743206743
C   -1.79498839686371     2.58135730935039    -2.43828419605092
C   -1.89648374600335     1.97009126575021    -3.70728656212950
C   -0.53817857008984     2.87105384111797    -1.85053547355620
C   -0.70930959124314     1.66698119899311    -4.40669754900526
C    0.63420269619392     2.49880695695105    -2.54852057216895
C    0.54561479612873     1.90978857991994    -3.82906138527469
H   -0.77364898354220     1.19584012920318    -5.38440143841439
H    1.60741430435250     2.71292032264129    -2.11289556203751
H    1.45296741293903     1.63257208410722    -4.35943327978836
C   -5.60859522491411     2.01817000201614     0.81271306272082
C   -6.82001993299335     1.42461821718702     0.38562745597123
C   -5.28180412857336     2.13527620682258     2.18426358794660
C   -7.70978842851682     0.93490377702301     1.36159917951383
C   -6.19000245350551     1.62186685491049     3.13340711721438
C   -7.39610704541691     1.02576350374097     2.72853970633096
H   -8.64430511235986     0.47528820568622     1.04721718369820
H   -5.95213369207199     1.70377386012501     4.19197175498615
H   -8.08891363201429     0.63914990725793     3.47257362364527
C   -4.06703802872804    -1.69995773606882     3.82068284653401
H   -4.45301080106046    -0.91001132658239     3.16048612973192
H   -4.56529176611129    -2.62936887481658     3.52405777254728
H   -4.36194194125967    -1.45439807150975     4.84708317780350
C    0.03997349008628    -3.00006137069277     1.11685237749738
H    0.00925398993054    -4.09389154587233     1.21786780706190
H   -0.51985342607859    -2.77213221672415     0.20086895416640
H    1.08688851885511    -2.70426835960149     0.98845267534594
C   -6.90455192606048    -2.22238440658660    -0.21517663234908
H   -6.89903565706508    -3.22870242129430     0.22265070456892
H   -6.41117937771958    -1.56086650100743     0.50626883599584
H   -7.94685879770115    -1.90102336533536    -0.31539347831211
C   -2.64715305143400    -2.73547572236224    -2.96705118740222
H   -2.37534937694366    -3.57148313377937    -2.31202965956385
H   -2.36363175716509    -2.98921492292376    -3.99511295386194
H   -2.03834830301250    -1.87596492541880    -2.65223623631360
C   -7.13728101712600     1.30508369308127    -1.08990105104096
H   -6.34602201921964     0.76311629572820    -1.62033820800912
H   -8.07964926180754     0.76938002100528    -1.24599132280207
H   -7.22062566866135     2.28993404980248    -1.56659019692192
C   -3.99612987951795     2.79750574560706     2.62823429318126
H   -3.13376116257130     2.13304729851943     2.47669954215065
H   -3.79790634155002     3.71028720853161     2.05364546674765
H   -4.03750794012220     3.05628427422571     3.69256458814837
```

C -3.24609847963269 1.56017429256754 -4.25251679007012
H -3.64363364537729 0.71177202815407 -3.67785379718896
H -3.98416718465610 2.36629020776696 -4.17386995509768
H -3.17225296027472 1.25282325528782 -5.30170552833420
C -0.45091836779457 3.60081228415871 -0.52687629201184
H -0.72365537811200 4.65778307473907 -0.65248094939420
H -1.15853179597784 3.20071391397624 0.21092976246310
H 0.56437095015874 3.56183322362911 -0.11759356342423
H -4.33909392956948 -2.05161835598836 -4.99488858667341
Sn 0.35009999685035 -0.27140000066398 -1.12230000097208
Sn -0.16630000202239 0.93419999662712 1.78019999917879
Sn 9.39839993035979 1.46220002579597 -0.94669996017157
Sn 6.69110000573097 2.59560000242442 -0.72830000366911
B 10.11280011133552 0.96100008825441 -2.98049996096788
B 10.64760008706852 2.34049988504726 0.65559985162728
Sn 7.32860000731035 -0.47120000038102 -0.15140000184667
N 9.34249993221188 1.24839996717375 -4.17240002457327
N 11.23579998109374 0.19469995957038 -3.46760000693096
N 11.55449995200187 3.46440005219310 0.70520005368494
N 10.53679998362414 1.92060002161207 2.03710005785198
C 8.12782748409852 2.00516199205444 -4.20140609198410
C 9.98640567775684 0.68636808026500 -5.29894649846717
C 11.12646565722829 0.05584269246822 -4.87431276857334
C 12.33614793784163 -0.35370323475217 -2.72630427192200
C 12.02893795085722 4.27075859061811 -0.38391274058991
C 11.96050441422986 3.67398157538275 2.04665681744823
C 11.34668026306035 2.74813902300338 2.84942488469187
C 9.75532624929280 0.81397751063671 2.49788633317856
C 8.18427517597260 3.39091915802479 -4.46645860194446
C 6.91867609777875 1.36867924011946 -3.82423783730814
H 9.58282368425472 0.77063166460175 -6.29704678483715
H 11.85707710283844 -0.49242249569293 -5.45047708708457
C 13.58448182862142 0.31131827925709 -2.76647868505796
C 12.14832011055297 -1.53535300526285 -1.97135342005990
C 11.18962047458612 5.27348173542496 -0.92444222971963
C 13.32278158670337 4.02681767620769 -0.90153834227051
H 12.63667737045374 4.47093585101459 2.31881690554822
H 11.41387427444340 2.61946412930692 3.91953312633085
C 8.36048904316345 0.99077329640965 2.67427709005290
C 10.36396485994522 -0.45412283129404 2.62357014745312
C 6.99607034980753 4.14560682419723 -4.36865752856985
C 9.50790866599448 4.06323374796842 -4.75399037550907
C 5.75772311362872 2.16245221926662 -3.67551352940641
C 6.85702479994285 -0.13209036841064 -3.63291989689530
C 13.75522296691970 1.57373441328233 -3.58426948873520
C 14.65454316737678 -0.22055152211688 -2.02089210442476
C 10.81915281680811 -2.25777062763181 -1.96468805686199
C 13.23770885067578 -2.03197721710301 -1.22561156235454
C 11.66393206439000 6.02255535998588 -2.02126472248809
C 9.81861563339247 5.54220197130056 -0.34433256736311
C 13.76941196172583 4.80336171548501 -1.98873923859949
C 14.19498668361756 2.94155100889058 -0.30696988385404
C 7.73732733087701 2.36990320971005 2.62313427415734
C 7.56657708820116 -0.14822565395665 2.94322401775008
C 11.83247846004342 -0.63377913149767 2.30920257637246
C 9.55425369749177 -1.56139851103819 2.95365542103474
H 7.02670093057146 5.21359522259172 -4.56967267755180
C 5.79639362477595 3.54512652921286 -3.96024626304785
H 9.36025040686496 5.09071643791877 -5.10494821355690
H 10.09341800236007 3.51745648042615 -5.50232060819912
H 10.12085688939616 4.10239763256244 -3.84220949256930
H 4.81988801875645 1.69148410127467 -3.38995378923577
H 6.89867151941026 -0.64350016641027 -4.60470805714374
H 5.92784793934414 -0.43125289542959 -3.13573009529729
H 7.71557492485085 -0.51190021178799 -3.06410408926001
H 13.01618297151447 2.33195914190713 -3.30119438114311
H 13.61567134338497 1.38213362368555 -4.65586341919971
H 14.75419503203906 2.00020517622282 -3.44314085468203
H 15.61924909258897 0.28178794298029 -2.04157423055362
C 14.48267721741589 -1.38123699969825 -1.24718613684866
H 10.37245750769237 -2.28158799934294 -2.96570006930424
H 10.09657006188579 -1.75489646641482 -1.30647337754935
H 10.93660689752235 -3.28806490652835 -1.60921975414511
H 13.10801624894640 -2.93952720704805 -0.63960724677235
H 11.02885447077535 6.79596352195235 -2.44829840446009
C 12.94403845862730 5.79139191702324 -2.55221521236045
H 9.44129563057728 6.51923700589732 -0.66769453145636
H 9.09575174966578 4.77890263552632 -0.66570386167233
H 9.83753762695382 5.51967334912768 0.75172018279775
H 14.76147566652023 4.62545548599087 -2.39804968460523
H 15.12743351367426 2.83456770729041 -0.87130509639730
H 14.45171880700015 3.15706255811802 0.73817733990318
H 13.68028807827785 1.97382972347964 -0.31266729863289
H 8.15601475586248 2.98107870043058 1.81374248444800
H 6.64909147472652 2.30895921448011 2.51132904287742
H 7.94807516001279 2.92057513218901 3.55057962925970
H 6.49462124425701 -0.03129633759618 3.08364095277327
C 8.16628666274631 -1.41779739786692 3.09447638428994
H 11.99746885677432 -0.56328987913793 1.22446079643993
H 12.45173231060083 0.14225510534700 2.77212262116019
H 12.19063369337458 -1.61472024113661 2.64114141992746
H 10.01046944393721 -2.54330995153814 3.05241535411514
H 4.89602735384050 4.14357378211265 -3.84903113568865
H 15.31484233236486 -1.77925079473030 -0.67075535739209
H 13.29844960859302 6.37987508008549 -3.39552246531625
H 7.54549600730221 -2.28373158349132 3.30827174149401

Sn
83
Sn 0.46524235512179 0.22069362026856 0.26049792932085
B -0.97527035303878 1.74164566562489 -0.63008043656583
N -0.55792034095724 2.67723758813393 -1.65981829178246
N -2.24512838690904 2.29058720275623 -0.17659246033387
C -1.52013679389061 3.70066363822586 -1.79875253979917
C -2.52877579208111 3.47444817556107 -0.90201229898546
H -1.40518425332023 4.50724481700969 -2.50767806479011
H -3.40882351051684 4.06802722942371 -0.70582950529650
B -0.80433293881893 -1.59746886472816 0.77143172490701
N -0.49654738441156 -2.45336288017844 1.90377004770091
N -1.75655940649194 -2.38866090008557 0.00507374588527
C -1.22188739855278 -3.66041572506144 1.80563616091981
C -1.97200167851012 -3.62575611251628 0.66168494414817
H -1.13810381971319 -4.44453847519713 2.54352992049108
H -2.62085825870787 -4.38488738601494 0.25179131167215
C 0.40138882901956 -2.17909193260244 2.99134826163202
C -0.11463001253583 -1.61769304066826 4.18331902209042
C 1.77868478957024 -2.46487900039392 2.84099127073968
C 0.78106801241266 -1.31978419778922 5.22919136971242
C 2.64661674021065 -2.15805246933460 3.90849850179598
C 2.15507052520581 -1.58394981088919 5.09291157309195
H 0.39916248898681 -0.88320564522300 6.14967066949605
H 3.70907844507258 -2.36783649756306 3.80610279525644
H 2.83738132475370 -1.34834473104779 5.90658710433786
C -2.45720613851778 -2.06799233266195 -1.20336847976205
C -1.73944466583665 -1.84859479147937 -2.40496768652874
C -3.87490809642469 -2.00524854453986 -1.17753449144864
C -2.46538449392430 -1.59871988196100 -3.58791270639907
C -4.56296171376619 -1.75570010343101 -2.37978542061286
C -3.86782554727866 -1.55882898650420 -3.58350704363697
H -5.64981021160718 -1.70715465956798 -2.36612024597354
H -4.41211788537883 -1.37191342816579 -4.50605976483422
C 0.62629567571652 2.61302266810303 -2.47106191733292
C 0.55086022608694 1.99653388431260 -3.74308730387898
C 1.83813429760119 3.15018461480310 -1.97759030019366
C 1.72496059791349 1.90082497322769 -4.51534474116961
C 2.99344504996369 3.04150701137984 -2.77851744509501
C 2.94178221717607 2.41645064522262 -4.03555029584002
H 1.68305936834502 1.42460059975166 -5.49271723137114
H 3.93355247133889 3.44682021334339 -2.41075233648434
H 3.84247464801886 2.33572969779926 -4.64003622587816
C -3.16577418258521 1.78820223815241 0.79984048254579
C -4.47300764736913 1.42305494400086 0.38516885917684
C -2.77574222578290 1.68502368455940 2.15767371998490
C -5.39324556147744 0.98738000597642 1.35699076347182
C -3.72509065844389 1.23861425536367 3.10014900448469
C -5.02872702753905 0.89762540194438 2.70982773807880
H -6.39750455983902 0.70706406399907 1.04614342149235
H -3.43995125567473 1.17589040875304 4.14788714644147
H -5.75079833962520 0.56240808052286 3.45039604285138
C -1.59990550306401 -1.36625796092521 4.33256915071150
H -2.01254488478779 -0.85470205316556 3.45783493389217
H -2.15317389713838 -2.31086455598186 4.42822969203776
H -1.80814883158033 -0.76266822761441 5.22347455213837
C 2.31071218077416 -3.06642121608681 1.55819154707468
H 1.66644426476341 -3.87435682229434 1.19271668628791
H 2.35360713938231 -2.30363434440107 0.76753915305239
H 3.32287509490987 -3.46145473486985 1.70086493685966
C -4.64931159479100 -2.17754798514325 0.11358426734236
H -4.76881965677871 -3.23523354646278 0.38369117887000
H -4.14195075770670 -1.68525432767451 0.94895643182133
H -5.65103331352401 -1.74496945645670 0.01936905636128
C -0.22567479211495 -1.88726590686668 -2.45941244160706
H 0.19382427553552 -2.57194356368349 -1.71645237321599
H 0.12005771847012 -2.19449448304635 -3.45335359337422
H 0.21037038657074 -0.88715363451590 -2.28617416649745
C -4.87931675779523 1.46853357588566 -1.07433355944002
H -4.07182197292944 1.11051006336707 -1.72094341145068
H -5.75878372130741 0.83883626026468 -1.24547174720652
H -5.13009231490657 2.48644879218580 -1.40075160882749
C -1.38124142452527 2.04836307138203 2.62500674435756
H -0.70602234854752 1.17435424668342 2.60028705646092
H -0.93558832593647 2.83600178371006 2.01037825466815
H -1.39602945765623 2.38401220719098 3.66859492129281
C -0.76981127282149 1.47025776482291 -4.26243193448798
H -1.27401352706753 0.84258547509456 -3.52108546039068
H -1.46139555475567 2.29233794094101 -4.49285430192134
H -0.62605590778898 0.88430296741436 -5.17752982473811
C 1.90068373198291 3.80792411001644 -0.61582959264353
H 1.02653080581092 4.44318353761456 -0.43360039062374
H 1.91845926238142 3.04550279771564 0.17615243805215
H 2.80607135329568 4.41741528038990 -0.51675412799661
H -1.92249791934085 -1.45085198610497 -4.51869876459241

Sn2
84
Sn -0.06885148951687 0.06189472044818 0.04288630612246
Sn 2.64532321301248 0.19652067996167 0.22657736595723
B -1.08456651247802 1.95628479888348 -0.47661913155763
N -0.35384494984235 3.01458958263068 -1.14888527112015
N -2.34544505775923 2.57279799297327 -0.13104740062769
C -1.14583037153064 4.18413093915480 -1.18995579521952
C -2.34436043722930 3.91620393328196 -0.58498086299186
H -0.79846806616012 5.09966611816288 -1.64513098172195
H -3.19277588893427 4.56658528668120 -0.43101190514872
B -0.98188872512086 -1.90900613428727 0.46335052277070
N -0.24405907572253 -2.89752513200077 1.22723364798615
N -2.14287549517909 -2.63212316131915 -0.00660957677523
C -0.93528158989466 -4.12953733534907 1.20100186983833
C -2.07839550816835 -3.96789517396585 0.46543835532272
H -0.56389038170008 -5.00969192267604 1.70438150459152
H -2.84610866409364 -4.68963370035871 0.22858007112147
C 1.00554037301370 2.96547560251812 -1.59697284779701
C 2.03191338319658 3.35966898622082 -0.70039078811890
C 1.29536620053948 2.48652119076176 -2.89450452617967
C 3.37351774811461 3.24759119744972 -1.12937557545828
C 2.64319197044781 2.41656763143644 -3.29934283297670
C 3.67706247890969 2.78577687752195 -2.42327980756013
H 4.17351921929946 3.53481436901007 -0.45126400040411
H 2.88040562337549 2.05076793058050 -4.29555265941510
H 4.71410757865305 2.70634600345895 -2.73913454293526
C -3.47047035366358 2.05658913635324 0.59707387875663
C -4.64098468291287 1.70454077918109 -0.11301204405566
C -3.40369847237880 1.97598404753308 2.00815798733871
C -5.77249370922036 1.29648147443030 0.61953315242374
C -4.54882988900722 1.54453632694196 2.70814342034149
C -5.73054574359793 1.21887486893060 2.02093963694372
H -6.68316815585032 1.03678937926691 0.08540037409439
H -4.51571046678797 1.48249251410259 3.79375714511988
H -6.61189465226764 0.90370666892144 2.57513251298368
C 1.03455969643212 -2.72067382218245 1.84703278624746
C 1.10272920004629 -2.22822494394385 3.16983482257148
C 2.20323409908576 -3.00103462264506 1.09345092413690
C 2.37200420601228 -2.02575840095218 3.74725172259361
C 3.45914067563876 -2.75668178087120 1.69352992009866
C 3.54309789346676 -2.27832667248193 3.01443095405318
H 2.43869907006677 -1.64735200289665 4.76453725351141
H 4.36588376170769 -2.95662688825038 1.12763312364907
H 4.51666799082739 -2.09624839657048 3.46230188681691
C -3.22911757598629 -2.21735365237299 -0.84985620213135
C -3.02542069315152 -2.12032301576519 -2.24673420749645
C -4.49550272442690 -1.98280772287232 -0.26597545357441
C -4.12785647968413 -1.79355573222055 -3.06251458909809
C -5.57814674483856 -1.67553766433996 -1.11250453114009
C -5.39939554867024 -1.58394417341746 -2.50226758308025
H -3.98858993280819 -1.72071125701247 -4.13901201827307
H -6.55937625558029 -1.50591046837442 -0.67592044976065
H -6.24413053641957 -1.34752677981311 -3.14539540258575
C -0.16271908042724 -1.88282126932810 3.92218931399945

H -0.62070594571354 -0.97723512853889 3.49900779917325
H -0.91123953499976 -2.67977634838478 3.84415683843624
H 0.04626228228776 -1.69727164467129 4.98161273791848
C 2.10271229298780 -3.58610003236901 -0.30142148145034
H 3.07791565408200 -3.57691435336361 -0.79997220363887
H 1.74638263153228 -4.62479553305182 -0.26185622217256
H 1.39279678867367 -3.02737547348490 -0.92017012615647
C 0.18100857778277 2.01721227753924 -3.80225395036158
H -0.22981827044030 1.06503601317545 -3.43713541755119
H -0.65098345410374 2.73046410732660 -3.82475179584548
H 0.54281919973085 1.86390025041301 -4.82502252572180
C 1.69185590539863 3.92497893511172 0.66412889808475
H 2.58847461526515 4.00801770869749 1.28784736805252
H 1.24603599012769 4.92496566070801 0.57032371427435
H 0.96297279130945 3.29785822110791 1.18803152418330
C -4.66171119977841 1.74814674653926 -1.62533342924797
H -3.94343347032730 1.02991711642611 -2.03960472824480
H -5.65262345253435 1.48919938437111 -2.01141656003455
H -4.38416445603616 2.73715780734288 -2.00956825800722
C -4.67056448433845 -2.04467007615893 1.23573156128631
H -4.03601032333148 -1.29706723781161 1.72718880943046
H -5.70822849133507 -1.84166190264277 1.51811140851031
H -4.38457350494677 -3.02351793156328 1.63983471892421
C -2.14324927369321 2.36655670733524 2.75161354126413
H -1.33681671162699 1.63943171009750 2.58257003147586
H -1.76318444062960 3.33699928642869 2.40868739942010
H -2.32809276065872 2.42634638527080 3.83019189756583
C -1.66369644903667 -2.38174811391660 -2.85535036764045
H -0.96061863307399 -1.56975787458161 -2.62259877902767
H -1.21902235884083 -3.30197515804203 -2.45688636742011
H -1.73281498456897 -2.47098871583991 -3.94542550766633

Sn2A
84
Sn -2.11233793104857 0.34813045995088 2.39318606479716
Sn -0.80126418628528 2.83849237369769 2.12494284014484
B -3.81094132721751 0.74451160537849 0.92397528571252
N -4.68245018410634 -0.25907510855552 0.33720217335852
N -4.36826967179394 1.99676185997518 0.43483623741302
C -5.68000958118727 0.37112810943484 -0.43805165206544
C -5.49816431320549 1.72861679316576 -0.37203826872969
H -6.44183973639727 -0.18964036790088 -0.95952689317140
H -6.07864430490762 2.51700687327942 -0.82827847185723
B 0.95178415149734 2.32322679611912 0.75481586058742
N 1.51926978989957 1.04251886700600 0.35827024905035
N 1.88590990799190 3.28721294158918 0.19338670663798
C 2.70788621002602 1.25879525370996 -0.37506392059617
C 2.92494751841260 2.60812942985016 -0.47884170325771
H 3.30612148038081 0.44186415199959 -0.75058759706456
H 3.73614352153103 3.13345072735333 -0.96088707650230
C 1.78310075924206 4.72051075478165 0.19992946877213
C 1.33467555528834 5.37919456196183 -0.97082265496276
C 2.11113085612380 5.44147789639899 1.37205810389698
C 1.18255487141296 6.77919389024133 -0.94252472919008
C 1.94803352111255 6.84187929460896 1.36491854686176
C 1.48003498343391 7.50902039911876 0.22102669234046
H 0.83080408527407 7.29497002620280 -1.83391059454819
H 2.19258769217103 7.40722435861490 2.26157018408926
H 1.35722581599381 8.58967572633066 0.23246510663836
C 0.96596731490973 -0.27038136492978 0.52957507049482
C 1.10218913509581 -0.93647124553004 1.76931556512518
C 0.26026043349309 -0.85658550657100 -0.55084271428053
C 0.48803453043050 -2.19758021554397 1.92541542283408
C -0.34520638131986 -2.11286733743587 -0.35670385703715
C -0.24182868735121 -2.77866111383824 0.87628987286933
H 0.56608050785447 -2.70843844850211 2.88191323462679
H -0.90486184872804 -2.56714120111634 -1.16852760500532
H -0.74028730534417 -3.73378514915674 1.01816515312097
C -3.82402724701722 3.31544006411564 0.57019618859419
C -4.28615319635871 4.17379017290969 1.59152287567751
C -2.80004174153849 3.71151422498593 -0.32767688555221
C -3.70929799331135 5.45604887877963 1.70257362437905
C -2.21771919894325 4.98697838946484 -0.16398524265576
C -2.67609992053430 5.85779168211557 0.84102333799965
H -4.05341348134378 6.12608126756559 2.48673615228282
H -1.41740702508461 5.30156134903373 -0.82575598301501
H -2.21189294845906 6.83333199382184 0.95857728138850
C -4.51890082185762 -1.68600268545084 0.37956596327391
C -3.91193523472785 -2.33095965073802 -0.72519849871989
C -4.93242829046715 -2.40757375412860 1.52243703549842
C -3.71089872232976 -3.72295737694503 -0.66672162191434
C -4.71262738392517 -3.80031480814991 1.54957822581822
C -4.10395264834854 -4.45593142470494 0.46660547715050
H -3.24527447767241 -4.23084792028209 -1.50915139686755
H -5.02411141572581 -4.36842260465089 2.42344232457911
H -3.94333886072857 -5.53118818442165 0.50195389787499
C 1.03213457267418 4.60019662436783 -2.23481101365915
H 0.57094433862747 3.63420372327735 -2.00642386019158
H 0.36614013709517 5.16654054045403 -2.89667437216711
H 1.95121823283241 4.38404665050338 -2.79690564404319
C 2.61779395630300 4.73764349974382 2.61233558279132
H 1.77749524167827 4.33303990281367 3.19477544619759
H 3.26963242874238 3.89454108520664 2.35882012961523
H 3.17050274670596 5.43160505994560 3.25613569737697
C 1.84683060251738 -0.29887383388678 2.92108371332907
H 1.17479283982098 0.35196496039880 3.50041968944365
H 2.23246780335784 -1.06164850847977 3.60713564316310
H 2.67969052767607 0.32009220440458 2.57278987587648
C 0.15871616213476 -0.14226753714930 -1.88202930916010
H -0.58176836649406 -0.62127859352998 -2.53080880663400
H -0.11749883433278 0.90907666249354 -1.74708897366604
H 1.12192393965883 -0.14674662872153 -2.40937756949643
C -2.38011730656627 2.78773169863854 -1.45012576735258
H -2.01944139304061 1.83155665168181 -1.05588890007948
H -3.23073286520469 2.55867202652025 -2.10527421240285
H -1.58965310305097 3.23925937221928 -2.05602212725109
C -5.34852342910476 3.70429881665368 2.56118952516051
H -4.97567783334176 2.86984418128965 3.17045698768766
H -5.64916835154283 4.51217842053588 3.23703199579330
H -6.24003275001691 3.33589723781826 2.03921310636496
C -3.47722140860817 -1.53135717234193 -1.93542851983774
H -4.33620395185706 -1.09790710382253 -2.46329153648300
H -2.83646563894280 -0.69240471836906 -1.63926831281432
H -2.92768699519637 -2.16027608069134 -2.64515163009691
C -5.58266886345694 -1.70585030144592 2.69480085887176
H -4.82435379352172 -1.19314915509146 3.30339705840751
H -6.29447337482974 -0.94214012023843 2.36140283725620
H -6.10718994502494 -2.42179427020872 3.33788482105057
Sn3
85
Sn 0.03474970027201 0.16261067725407 0.20692907869415
B -1.09887433695642 1.94508984601761 -0.45304347838980
N -0.59845157338741 2.86283502172144 -1.45490453448725
N -2.30398784805499 2.57845227863660 0.02912046487541
C -1.47217322649768 3.97013300063516 -1.55995488143350
C -2.49891715702711 3.79614957949617 -0.66911987108233
H -1.29240591902122 4.78592063240972 -2.24432194717872
H -3.33956417430416 4.44203318469062 -0.46294466466967
B -0.84000391485658 -1.84181423324081 0.54859617589281
N -0.43985206613494 -2.67801713340283 1.66113074411708
N -1.70956389388823 -2.69260362478958 -0.23123940165897
C -1.04783391577991 -3.94903254884611 1.54140098426258
C -1.81362722076508 -3.95511691419342 0.40510992581648
H -0.88236491847779 -4.73904229427802 2.25877595027718
H -2.40791237460254 -4.75458265945477 -0.01216541225520
C 0.40311838974200 -2.25564547701627 2.73750515968374
C -0.18620309948712 -1.71162823364387 3.89983061689424
C 1.80671089874516 -2.25700465080839 2.54494913556263
C 0.65734368897668 -1.17614859081360 4.89593858061304
C 2.62204498159228 -1.65987159458432 3.53402078529598
C 2.04683049165128 -1.13219064824206 4.71106883242726
H 0.21662445087621 -0.75282017544998 5.79512247598989
H 3.70192348681452 -1.65604415167208 3.40643460919619
H 2.68527982600434 -0.68181592180728 5.46631484440142
C -2.43628032033370 -2.37743810769249 -1.42914811802273
C -1.75803174159742 -2.33520496185734 -2.67025347687536
C -3.82263628591840 -2.10803555504584 -1.33192979456264
C -2.49618887627107 -1.99780369212375 -3.82413460319204
C -4.53056992656871 -1.78967692719298 -2.50698850508992
C -3.87243665262780 -1.72752755281601 -3.74709433306274
H -5.59623774894219 -1.57991197660950 -2.44556061571796
H -4.42924848897267 -1.47465179392750 -4.64657894070198
C 0.57983384306233 2.65650206352644 -2.23995498407421
C 0.46666982866106 2.03707116447897 -3.50392828895630
C 1.84090357390295 2.94878436895863 -1.66405851745543
C 1.64758984551925 1.72908421323777 -4.21166826699391
C 3.00662205333991 2.57394108859283 -2.37099763836766
C 2.90732379951261 1.97727127589344 -3.64708859097403
H 1.57479332824540 1.25185626516083 -5.18579764479573
H 3.98342821400714 2.79376642893146 -1.94684018507639
H 3.81107876802134 1.69990553694241 -4.18299967567857
C -3.22637286811762 2.10047267710365 1.02035405736958
C -4.43960034474107 1.50992227454870 0.59405366633476
C -2.89841418312960 2.21635987740677 2.39177282924768
C -5.32954399200637 1.02156778266539 1.57054796299298
C -3.80682995423172 1.70414423049866 3.34138342981027
C -5.01446337933939 1.11070293954651 2.93725140354638
H -6.26542019123502 0.56433865152968 1.25675132759804
H -3.56802359799059 1.78517169546739 4.39980057155814
H -5.70752753864246 0.72529687390245 3.68165910181238
C -1.69045527148722 -1.61646833639729 4.02381731121928
H -2.07592113993241 -0.82687499118498 3.36293439371070
H -2.18733856423521 -2.54634669881214 3.72639894063183
H -1.98752730567805 -1.37105701389068 5.04960458059660
C 2.42220306427008 -2.92020806457535 1.33096877123562
H 2.39182791729569 -4.01378879052166 1.43466646651778
H 1.86407002451656 -2.69453429073965 0.41343781338388
H 3.46925730105370 -2.62432628683225 1.20365192247440
C -4.52058669025755 -2.14029975018573 0.01135606363051
H -4.51322854471021 -3.14704185890224 0.44816286482851
H -4.02209499271417 -1.47975282119732 0.73020286235086
H -5.56317955989311 -1.81783316950426 -0.08177989407121
C -0.28080589250426 -2.64575057983150 -2.76962423530572
H -0.00370089685483 -3.48440649613986 -2.12021169526491
H -0.00300582995951 -2.89432894991483 -3.80048285036292
H 0.32851754989904 -1.78675582583174 -2.45447884255313
C -4.75944628249713 1.39286133706790 -0.88110454518096
H -3.96889875922076 0.85246080264110 -1.41413702456249
H -5.70172399607425 0.85676943790946 -1.03627802010066
H -4.84466411459411 2.37856124505046 -1.35572285722520
C -1.61158725548615 2.87645142246097 2.83564348179368
H -0.75028919837509 2.21043431749601 2.68516657679983
H -1.41157152198767 3.78850193877455 2.26052909717860
H -1.65299197788405 3.13610676307363 3.89975010434852
C -0.88802726012450 1.62556835852445 -4.03522834145989
H -1.28245766967593 0.78155524994681 -3.45213340596621
H -1.62412672150853 2.43347315236945 -3.95608489603654
H -0.82322838771589 1.31121237502952 -5.08290786185350
C 1.93799786641985 3.68350624988730 -0.34377952458938
H 1.66376938437230 4.73990896961441 -0.47068863248899
H 1.23661240397347 3.28522268338087 0.40088134917635
H 2.95615437866920 3.64584321922692 0.05848545807675
H -1.98756676733090 -1.96229950031586 -4.78536149848414
Sn 2.73240357497369 -0.19013997623566 -0.91542813032513
Sn 2.21600369621853 1.01552166881460 1.98706785416000
Sn6
170
Sn 10.05750321170962 7.71563745112766 13.30620491567773
Sn 11.12284853971505 9.90424009434942 14.89408800574405
N 12.28239638430261 9.46352194346483 9.11296994959314
N 9.28484281584436 6.77131019815237 19.36801538340532
N 11.90444510552378 11.47352060067548 10.12125219074216
N 11.05000161375769 6.54222505140544 17.94034617148357
C 12.15302566422040 12.80095636009074 12.17068820704773
C 9.95017932167984 14.39490193760742 11.39968239023015
H 9.10470720310347 15.01086915791522 11.10081487559651
C 10.37804418356548 13.35791447731649 10.54668910660357
C 11.70335738299555 13.84898785949287 12.99601563157873
H 12.20244376700458 14.02851803050444 13.94536380557711
C 10.60469047393267 14.63904290175452 12.61839862711003
H 10.26480189201232 15.44133744225593 13.26959046205005
C 11.47149181383245 12.56165494578898 10.95348404886632
C 13.89606342928843 6.70494319212509 14.80154973288002
H 14.61927037408596 6.75177630043315 13.99065046082721
C 13.32178048059057 11.93453188471972 12.58759591475375
H 13.07625846086934 10.86979621282952 12.49085243382457
C 11.84282399397689 5.77290644122975 15.74868510804560
C 12.80050191312778 5.83055089384291 14.71644622399788
H 12.67123498258884 5.20087443977233 13.83915049701887
C 12.01521039459876 6.60888085720507 16.87791420552364
C 8.13736814847929 7.16923852612358 20.13330598351213
C 12.31885010759376 8.10454826172651 8.65054243797517
C 13.11480978767665 7.49106503805320 16.98462953700412
C 11.29528431802947 8.90662255845763 6.46633881571965
H 10.65347343153022 9.58623836099506 7.03499480272955
C 13.11397326384640 11.55033586767176 9.39201692670517
H 13.70257435230613 12.45501985924199 9.36021669390155
C 9.92734429692206 5.53643336836758 19.62889433915673

H	9.57456325612812	4.86274915180724	20.39541570220544
C	9.67610307978700	13.09781120363036	9.23523250867405
H	10.38532369294472	12.82418592258368	8.44634171778428
C	10.98235178682818	5.39920724002988	18.77230175310219
H	11.69434715943085	4.59206417514733	18.68654613201328
C	13.40574923141786	7.45117546827606	10.86126170357753
H	12.58221903923940	7.67477950884063	11.55233323579792
C	11.85437880514347	7.80988975030375	7.34773470927937
C	14.04632505600552	7.53129534392905	15.92651418211959
H	14.89186055770259	8.21217480827463	15.99281555273352
C	13.34024720271815	10.34743848013715	8.78426307010112
H	14.15854057604755	10.04056783003981	8.15000047256835
C	12.97613518819631	5.79394713041067	8.98077947363721
H	13.41613500183490	5.01809771046818	9.60348755852922
C	10.63735723476824	4.86311422364684	15.62942162881899
H	9.70954515064841	5.41828154397559	15.81488234936749
C	12.88794305540517	7.10888103528662	9.48071023376427
B	11.32802631956203	10.15089157498252	9.95747879498724
C	8.33122169843084	7.63062220439399	21.45717261469916
C	9.72210191808835	7.68677924272444	22.05247103064406
H	10.41315296859584	8.23512915647345	21.40318096302397
C	12.51563894095452	5.48174911629543	7.69043771419522
H	12.59395624816603	4.46294617652256	7.31750257286779
B	9.98056617874469	7.45829697544917	18.29730798761960
C	6.84453763348307	7.04284875821187	19.57381620138392
C	13.29183033490103	8.36884153449468	18.20353024587639
H	13.12788703029726	7.80843786116012	19.13146283764474
C	7.20341817555027	8.01845669111228	22.20577842158504
H	7.33872077028300	8.38418648543702	23.22134831044741
C	11.95398371174437	6.48386485342451	6.88163826173175
H	11.59617038898611	6.24195678751779	5.88328283535394
C	5.91329184938204	7.93807400554475	21.65344829354800
H	5.05030392372012	8.24478497987292	22.23998896993771
C	5.73813663212729	7.44466236068538	20.35055908836361
H	4.73758244043772	7.35710555561217	19.93242041581140
C	6.64246743033355	6.46253073953028	18.19039271858204
H	6.84634884716560	7.20219803430731	17.40461688169175
Sn	9.49440599969997	9.39785384121408	11.00781972058101
Sn	9.45983817600200	9.35187884500809	17.21223692788629
Sn	8.91279371908950	11.02614874600603	13.33746756098901
Sn	7.83233840965390	8.80464774284086	14.86820245115207
N	6.72099903143346	9.37772342822438	9.09406106463959
N	9.62727195841522	11.96070386121394	19.32202134540955
N	7.09262646399466	7.34256925172955	10.05326526864223
N	7.84376388833230	12.16365554280545	17.91336794045817
C	6.83043275550861	5.96117150191777	12.06536096764210
C	9.04227495650714	4.39170665029854	11.26996720720807
H	9.89113527100798	3.78522168212858	10.96151785303814
C	8.61926508949124	5.45023207292298	10.44136394271173
C	7.27540494599468	4.89222499555484	12.86604706479246
H	6.76890848240515	4.68640827524851	13.80608449090616
C	8.37850118939100	4.11425396084752	12.47644842591486
H	8.71402945068164	3.29474398100595	13.10820528061093
C	7.52097141241044	6.23361200003293	10.86015637160120
C	4.98032710446527	11.92279992643025	14.79406866320842
H	4.25579127569116	11.85208095545034	13.98611565967007
C	5.65642239483299	6.81355810841832	12.49610544587839
H	5.90091446745479	7.88095737031407	12.43222965015541
C	7.01251245607961	12.91344828641676	15.72743286479887
C	6.05175749582647	12.82504603126329	14.70006743019561
H	6.16186478099989	13.45225133274743	13.81829435892875
C	6.86945667229822	12.07724322880971	16.86075722583630
C	10.78990210951680	11.57321914686390	20.06967172219641
C	6.68953862456200	10.74761169757189	8.66487896331154
C	5.79116976989801	11.16999384943806	16.97809794242868
C	7.73204226322249	9.99636698167961	6.47218800339214
H	8.31099826575271	10.43299919003205	5.65052810998118
C	5.88794850771722	7.28442352293607	9.31417075533336
H	5.29970308889175	6.38086203919890	9.25523041995710
C	8.98187117911553	13.19507905352394	19.57676214499159
H	9.33954010229827	13.87899683295693	20.33191255401998
C	9.33052368327714	5.74553520464020	9.14232126914573
H	8.62556310989654	6.03149212615313	8.35386749261903
C	7.91590689024130	13.31677802996245	18.73108571197908
H	7.19877511326074	14.11925663293128	18.64490392627103
C	5.58442991136786	11.34894787770490	10.88155443206720
H	6.40214630633206	11.11860318250142	11.57740938448990
C	7.16589986556078	11.07298065381296	7.37365470564009
C	4.85596851051584	11.10015193032789	15.92514935681554
H	4.02784329547297	10.39903456373557	15.99996223143307
C	5.66536574690635	8.50256180594717	8.73599794560059
H	4.85134322220999	8.82550828566976	8.10421836326897
C	6.03209727166836	13.05025544303612	9.04517330290914
H	5.58764338992037	13.81140531784587	9.68268410553954
C	8.18625403925277	13.86274844651783	15.59844747669777
H	9.12615282862313	13.36513555071839	15.86484483083563
C	6.11444091602581	11.72347809879570	9.51405234844395
B	7.66967536480538	8.66892335923158	9.92681391639066
C	10.62073720438703	11.09617606870048	21.39099156228921
C	9.23993614842017	11.02775699709690	22.00766265098627
H	8.54662393335607	10.46170527320052	21.37642392747379
C	6.50394117566657	13.39284674490227	7.76674109492611
H	6.42982743088473	14.42041862056396	7.41776358147148
B	8.92228696063433	11.25718275151993	18.26920442754724
C	12.07241435489116	11.71992639980743	19.49181927458022
C	5.64005433797280	10.29654245420026	18.20348110617140
H	5.79621174158350	10.86639309065182	19.12689357151859
C	11.76369809830993	10.70888145872259	22.11678437634743
H	11.64836334816746	10.33164233160795	23.13057882132290
C	7.07157898362473	12.40986271146422	6.93869760637618
H	7.43830642665748	12.67542168615563	5.94964510770173
C	13.04395434586399	10.80611623692042	21.54436302193296
H	13.91862644795121	10.49902355936827	22.11320191606727
C	13.19462114001632	11.31836625355634	20.24554115085108
H	14.18760515213600	11.42008163744406	19.81285561365720
C	12.24480715107427	12.32141931489479	18.11351527379564
H	12.00707869427172	11.59790640387149	17.32234835865703
H	6.92787494163814	9.38878511227250	6.03359062138654
H	8.37442651227200	9.30815778392751	7.02985696259112
H	10.71776477499914	8.48832031393114	5.63416203870384
H	12.10342090873160	9.51929881956452	6.04225594157247
H	13.97609542337890	6.61521522772846	11.28258379339043
H	14.05086456074636	8.33801598690354	10.83961785258247
H	5.00211308045296	12.17133445388629	11.31306552841539
H	4.94776281288928	10.45709418373393	10.83512927763607
H	5.37144460447449	6.58955925802090	13.52955699875696
H	4.77950776408895	6.65050420332814	11.85545355166327
H	9.90599417823361	4.87908216535041	8.80424238046676
H	10.03678725555693	6.57672110302469	9.25517056996786
H	10.57603286542565	4.43143597372239	14.62528650413851
H	10.67168707323845	4.04277108461772	16.35773591808573
H	14.29688829324840	8.80465827530219	18.23063917590369
H	12.57244684941535	9.19586599658095	18.20928681404096
H	9.27332029267792	10.55514988727684	22.99472698927042
H	8.80840297137287	12.02969004776155	22.12759944996657
H	13.27816355533060	12.65408238869511	17.96184947546248
H	11.57467280943376	13.17640470973778	17.96656329838394
H	9.70936100309359	8.17207609331876	23.03392413508544
H	10.14181380992105	6.68067524774640	22.17955406714006
H	5.61018905591541	6.11679348997730	18.06231923063179
H	7.32081875819677	5.62082521236979	18.00985177524328
H	4.64543974127167	9.83795999878820	18.23869401090364
H	6.37830554045275	9.48689907552435	18.21079641415201
H	8.27675455467781	14.22837698924862	14.57057117348031
H	8.08104027273198	14.73011745876482	16.26377217629614
H	13.59782824072310	12.12853824013537	13.62949637797774
H	14.20454630332535	12.11419384356024	11.95953287642530
H	9.10991402238978	13.97611098917860	8.91217632702764
H	8.96232324592343	12.27000181355870	9.32321422199360

TS1

84

Sn	-0.47165342485299	0.50746117755026	1.60785590391956
Sn	2.15805441640355	-0.01623402908802	0.80428494677131
B	-0.37788319210731	2.11410650847832	-0.14341766674237
N	-1.57836202098310	2.87927146036254	-0.47586675436284
N	0.65974437861538	2.82281192731537	-0.87898925718620
C	-1.25056634962217	3.94083127460975	-1.34462671346630
C	0.09517525100788	3.90846411164098	-1.58685190175551
H	-1.99433447027602	4.63397020881492	-1.70891437043446
H	0.69742619442450	4.56504237709921	-2.19721735547469
B	1.10168741560908	-1.91513398335601	0.01185020588500
N	0.42436564680348	-2.53241020152910	-1.10985891937209
N	1.43305825594505	-3.04684869185660	0.86638567846981
C	0.36830901709236	-3.93818689478392	-0.90956485956149
C	0.97103503780336	-4.24514675991847	0.27875349129378
H	-0.09689229392595	-4.59195606401239	-1.63257085411076
H	1.10901485547699	-5.20753686861804	0.74940877205790
C	2.15414189743410	-3.01231297699033	2.10865193422948
C	3.57246590256493	-3.03594968811027	2.07929224759944
C	1.43714825482518	-2.97182444684783	3.33030521145566
C	4.26881788447448	-2.95240805516644	3.30148278128760
C	2.16842628260298	-2.89127455313191	4.53237233501811
C	3.57346935855635	-2.86755343848300	4.51957624379631
H	5.35637658349750	-2.95930715804115	3.29423608246005
H	1.63206875199834	-2.85180045348493	5.47783041651974
H	4.12386591717843	-2.80013873551765	5.45533379398950
C	-0.00935657564653	-1.99262435968867	-2.36662289294175
C	-1.32571132072290	-1.50046638620336	-2.50879919187096
C	0.88807237285893	-2.04396403470126	-3.46094572158449
C	-1.73965398784115	-1.06385646275582	-3.78567274759873
C	0.43945947428271	-1.60846824174422	-4.72172761031372
C	-0.86890676332916	-1.12105229800349	-4.88550379766116
H	-2.75381069409284	-0.69654362024560	-3.91909270336813
H	1.11692586964195	-1.64888343234891	-5.57197892705472
H	-1.20933686295329	-0.79201478381122	-5.86465340669439
C	2.06879230171576	2.57634813670084	-0.98158163624934
C	2.95045111931525	3.18133567180428	-0.05098345076321
C	2.54798533531481	1.80470052151235	-2.06948639237854
C	4.33306278603317	2.96956027660640	-0.20677129513617
C	3.93872712152567	1.60470892284219	-2.18609056857963
C	4.82496917365002	2.18138371577120	-1.26190694745723
H	5.02236004923173	3.41364019568947	0.50725619265263
H	4.32109455699248	1.00249057500238	-3.00701529572076
H	5.89513068098307	2.01656332340234	-1.36151967262357
C	-2.93576867295387	2.68109306795991	-0.04505586118235
C	-3.86013395708055	2.09893483814562	-0.94546067316135
C	-3.32898473688755	3.10150629605711	1.24706189409998
C	-5.18563639116647	1.89725071185081	-0.51455054089929
C	-4.66435331826670	2.88722297083465	1.64457432607722
C	-5.58677808221087	2.28141121567975	0.77624692582075
H	-5.90237171256860	1.44091190004595	-1.19418953631688
H	-4.97809839481333	3.20086711496195	2.63797972699348
H	-6.61248342746389	2.11824429971262	1.09910986045225
C	4.32181914153495	-3.16175974161965	0.76865437381708
H	4.24938837087562	-2.23340709762265	0.18604706068431
H	5.38275475864204	-3.36954850961523	0.94739117706900
H	3.90676989005119	-3.96405088375639	0.14697482723476
C	-0.07474151024819	-3.03204280035428	3.34821424350863
H	-0.51571233815475	-2.08900630845723	2.99788297942594
H	-0.45431697976133	-3.82175307299649	2.68970477308582
H	-0.44415003014535	-3.21661316071883	4.36327108080793
C	-2.26843356197314	-1.44276546898568	-1.32580264542341
H	-2.09898752990060	-0.53279050753828	-0.73205529030288
H	-3.31392086393185	-1.42202824540233	-1.65295102411002
H	-2.12535323430267	-2.29523500412012	-0.65459008545899
C	2.31014760464694	-2.52529712407869	-3.26257588271892
H	2.86010710939221	-2.52532496745142	-4.21037548783692
H	2.84215456839865	-1.87842122894090	-2.55112229578802
H	2.34115151068985	-3.53856118831774	-2.84380147164164
C	1.58568147745391	1.25349368282462	-3.09638362132256
H	0.77277886147460	0.68842839049544	-2.63081651295477
H	1.11366270053552	2.06481531397081	-3.66648227701937
H	2.09826947125286	0.59545075970270	-3.80248508065445
C	2.40700090987181	3.99453757964565	1.10130738363749
H	1.90753272379069	3.33840367212779	1.82850552489442
H	3.21124152242786	4.52546751856861	1.62185302949446
H	1.66100308105152	4.72434340931952	0.76598144399440
C	-3.44241184231280	1.72691789768583	-2.35228155196065
H	-3.42364490284897	2.60973751049843	-3.00680965118182
H	-2.43693217535902	1.29802499678609	-2.37593012288300
H	-4.14379284994805	1.00637586962761	-2.78831271964911
C	-2.35160136908014	3.76613309535290	2.19311404500483
H	-1.78534183235049	3.01061713937599	2.75858699271096
H	-1.62358939943600	4.38297754907245	1.65567069560238
H	-2.87901413357682	4.39452163240003	2.92026237525196

TS2

168

Sn	-0.45156657654171	-4.07596203611458	0.68585879376878
Sn	-2.86146749312082	-2.88888998637429	0.50165471862021
B	-4.42298147228837	-3.47203290678644	1.95699534560698
B	-3.15325759956229	-1.57912095311126	-1.26693290732087
N	-4.22714615102524	-3.45136178875792	3.38569608821910
N	-5.73462361864105	-4.04311098499528	1.77794063584536
N	-3.83025518375559	-0.30823436779304	-1.39172939205443
N	-2.47592856290875	-1.74835378962682	-2.53327297989587
C	-3.05649082932257	-3.01613349864350	4.09978005103048

C -5.37616977603769 -3.97891154873857 4.01510571075469
C -6.28365376686988 -4.33374133116043 3.05023599844380
C -6.43721566969918 -4.29279429480478 0.55147289706695
C -4.62414095073503 0.40810921812164 -0.43354909602453
C -3.56753119388131 0.22826811390904 -2.67571147355775
C -2.75138908567213 -0.63574444805555 -3.35692898789778
C -1.56668723673669 -2.79170265014175 -2.92302558957074
C -2.88803697960706 -1.63786921979241 4.36560442389449
C -2.08127521882591 -3.97173321367472 4.46299184280705
H -5.45420859032558 -4.06023011209893 5.08902565025466
H -7.26096638347748 -4.77906014238708 3.16172087937004
C -7.49252216480548 -3.42539637573655 0.18053904144052
C -6.05141214902897 -5.38490903545531 -0.26113532509981
C -3.98343035839872 1.12521944193675 0.60447273288727
C -6.03215436642174 0.40090443278781 -0.57464539233956
H -3.97596724788561 1.17742998648606 -2.98927811717643
H -2.35472446053977 -0.55574579150309 -4.35794728159476
C -0.17693576304435 -2.53136265192643 -2.88984135847333
C -2.08279224733839 -4.05810517888151 -3.28168177454907
C -3.95062867271097 -0.63815751808531 3.96117787135018
C -1.70869562919112 -1.22244818600351 5.01404206343810
C -0.91456447865290 -3.52183223699571 5.11176922582695
C -2.26685450879551 -5.43368932393264 4.11843839716895
C -7.87830172114750 -2.25984433574958 1.06666307247060
C -8.16216809670701 -3.66523376381933 -1.03475642720790
C -6.73828812987558 -5.58832688792204 -1.47596608546997
C -4.93915046572310 -6.32584936064602 0.15630607479376
C -4.78780350363640 1.84007489076551 1.51580908280597
C -2.47532387272404 1.13244847953104 0.74103578724240
C -6.80311687176564 1.12529822704000 0.35452191505466
C -6.68621107254676 -0.37305515574662 -1.69941795129080
C 0.35041543070639 -1.18012985476241 -2.46175062863820
C 0.70519137660807 -3.57634778691951 -3.22543196087986
C -1.17071986081973 -5.07900502972685 -3.61350109509831
C -3.57610536357223 -4.31387868592095 -3.30937209906071
H -3.67536102256445 0.37452845866058 4.27565367544534
H -4.92340670846677 -0.88468194407972 4.40623289789137
H -4.10298835093551 -0.62247320576293 2.87633580374719
H -1.55912468969667 -0.16573270634573 5.22425266181209
C -0.72687290482413 -2.15681883682682 5.38502401602300
H -0.14721367755917 -4.24223314199551 5.38336638762130
H -3.24122303690559 -5.81252096944756 4.44979456258355
H -2.21572177590477 -5.58220435938095 3.03057403633462
H -1.48046607298719 -6.04559140599131 4.57283697463433
H -7.00533830751928 -1.64805645147625 1.31997772651607
H -8.61422927380849 -1.61863653265128 0.57033165788784
H -8.31213370318485 -2.59999669969655 2.01572741462552
H -8.97344991663163 -3.00526879521631 -1.33403843774376
C -7.78571383255066 -4.73662120612206 -1.86302809246178
H -6.45436479166283 -6.42391430319054 -2.11227104177675
H -4.94586773553335 -6.50018809134696 1.23772404353301
H -3.94809450832148 -5.91860848225753 -0.09076168136739
H -5.03519323005347 -7.28996464941751 -0.35593121071329
H -4.31047512023938 2.40224497275613 2.31547310074798
C -6.18669931266891 1.84073781543693 1.39584249741966
H -2.12891572179554 2.02681943046275 1.27208943327763
H -2.12149470209151 0.25556710004403 1.30193061034861
H -1.98674132808958 1.09884182859350 -0.23684364589866
H -7.88687710371620 1.12632324771277 0.26127677579189
H -7.77766249559830 -0.34382116248367 -1.61287581622737
H -6.41286968424758 0.03577643360356 -2.68063649862628
H -6.36924605712109 -1.42227151514354 -1.69610330916163
H -0.11071822900970 -0.86884241941013 -1.51645170358573
H 1.43437263830657 -1.21167395510460 -2.32173073328609
H 0.12888520942854 -0.40632776294597 -3.20718355791544
H 1.77527201512521 -3.39871081451459 -3.16978162360759
C 0.21425986990224 -4.84238432659010 -3.58348603323535
H -1.54931449967083 -6.06025533168263 -3.89134089098623
H -3.99061676879061 -4.41109878141640 -2.29748472103765
H -4.11673168863649 -3.48852114382084 -3.78755314629078
H -3.80257722843679 -5.23992986412702 -3.85016876635528
H 0.17784764220453 -1.82021974232305 5.88510767851605
H -8.30802476074804 -4.90829596604586 -2.80133425594856
H -6.79344709935817 2.39791982722840 2.10584772743130
H 0.90709430297379 -5.64464773901768 -3.82665882665245
Sn 3.58954229937725 1.53664774935665 -0.14665475846748
Sn 1.91288771805409 -2.82831274270865 1.04144467056189
B 4.46170777267509 2.72515612889950 -1.87528185433713
B 2.20434553485800 2.97669911873503 0.95463286597013
N 4.66880854536273 2.19436010447633 -3.21045881865887
N 5.25351630434455 3.94678602448159 -1.87136292605029
N 1.21769861295792 3.96887629241282 0.55018124759791
N 1.90833326804929 2.77509057295343 2.36386364583758
C 4.11119955788358 1.00381574303123 -3.79199732815768
C 5.52109220829388 3.05603014030157 -3.93851600273532
C 5.87955571701951 4.10234440244235 -3.13350729239774
C 5.40510602481223 4.91939743556481 -0.83055263171828
C 0.95933705888614 4.56466138293307 -0.72815517165737
C 0.41085650995271 4.30842312967191 1.66353861605056
C 0.82928629297295 3.59355604630342 2.75343000264108
C 2.48773655873287 1.79688614254904 3.24201341004262
C 2.98393570591906 1.12095998180893 -4.64179327756346
C 4.70098832828978 -0.25297521096121 -3.51580945963741
H 5.81432169826244 2.84708855627023 -4.95669271759051
H 6.54317909650277 4.92670619207816 -3.34639022468010
C 4.94557633220508 6.24319295709676 -1.05125150846900
C 6.00577258748266 4.55235434495813 0.39928535178013
C 0.51194031090066 3.76147556272216 -1.80567338355980
C 1.09980314571907 5.96898203689103 -0.86924140489147
H -0.39976562515176 5.01711248902102 1.58433209339667
H 0.44746511965220 3.60189591441940 3.76369483808101
C 1.86142285147358 0.53381564617334 3.35249611611118
C 3.67509043362012 2.10320425184734 3.94300375049391
C 2.39986909362501 2.48044039019361 -4.95996462138125
C 2.43499348835642 -0.04923925215035 -5.19980721833942
C 4.12200363269498 -1.40410469405418 -4.09049222859073
C 5.92274991775564 -0.36854045640806 -2.62960292474776
C 4.25994988739945 6.63311603341343 -2.34487085603125
C 5.11498838916570 7.19661377709663 -0.02927802592016
C 6.14864478679815 5.53286166300513 1.40254955891129
C 6.51340320523247 3.14853249186853 0.65755466452975
C 0.17733295371276 4.39397310587849 -3.02075510739474
C 0.37444867069263 2.25787764680357 -1.69045849319936
C 0.75741203396145 6.56225185019937 -2.09885000878990
C 1.62337819042629 6.82553019152872 0.26594493842887
C 0.60427142209203 0.23086916090513 2.56719650872645
C 2.45123847908821 -0.43916455302154 4.18237057774915
C 4.24351479720000 1.10597125822604 4.76147000254803
C 4.30381761653281 3.47662842697106 3.83108369237365
H 1.40926618123064 2.38400099860472 -5.41906370822613
H 3.04142435137012 3.03277776962491 -5.66060570468645
H 2.31118742968402 3.10183191621605 -4.06397408581030
H 1.56499668582854 0.02673975201841 -5.84848950845613
C 2.99619242510102 -1.30709699246393 -4.92408281592739
H 4.56533251822872 -2.37633403053886 -3.88581124299058
H 6.61790571627839 0.46195543997294 -2.79556546056813
H 5.63612775677906 -0.34512646323092 -1.56850008392774
H 6.45001037757330 -1.31171393854448 -2.81324468019768
H 3.57797699047858 5.84752080475197 -2.68471094141354
H 3.68212075507629 7.55357180250058 -2.21023108391175
H 4.98104086743996 6.80959182318126 -3.15410435219261
H 4.76331460555185 8.21324283253342 -0.19184494069719
C 5.71236283221200 6.84986422743721 1.19335754117502
H 6.61833451407131 5.26049844154212 2.34544763328887
H 6.84862826053883 2.65836496440635 -0.26092185259962
H 5.74159265775525 2.50153635456416 1.11640066782054
H 7.34377442702060 3.16284955650063 1.37318372077804
H -0.19028683254061 3.78836204031226 -3.84586805395961
C 0.29073953611562 5.78431744276236 -3.17056551073608
H -0.44021980427330 1.88734339955227 -2.32344820119731
H 1.28992702226722 1.74965550494269 -2.03442775360559
H 0.18243935406830 1.94295232327761 -0.66168345805430
H 0.86388483064730 7.63882971551326 -2.21503980976312
H 2.00522028712808 7.77786406248807 -0.11798561371425
H 0.84047986490184 7.05566690478154 1.00107965941819
H 2.43318996404436 6.32177266256493 0.80272978178249
H 0.80466126594475 0.25364149551011 1.48589214838485
H 0.19744585971980 -0.75057412356934 2.82755874473174
H -0.17778606364755 0.97718067013127 2.74940530429786
H 1.99314946444075 -1.42154127721443 4.25710278598853
C 3.63802624423945 -0.15716717134643 4.87987602428643
H 5.16109379704458 1.32263667382781 5.30443020469069
H 4.44127987632427 3.78026597029975 2.78862339976910
H 3.66249431642032 4.24242294560549 4.28940820675294
H 5.27673623909635 3.50370955022940 4.33543912174669
H 2.55805686554527 -2.20254357948157 -5.35732783653331
H 5.83532958713418 7.59699916839161 1.97367837815078
H 0.02119780769234 6.25602233241229 -4.11251921898589
H 4.09228452046449 -0.92122288903416 5.50649116933745

TS3

170

Sn -4.66872207933677 -0.57022597581145 0.43579297180841
B -5.82865786833532 1.27863299479722 0.07501707791577
N -5.38631911386954 2.27808499664041 -0.87845907017877
N -6.96348003091344 1.89522001145593 0.72354199138480
C -6.21690592492841 3.41881100458491 -0.78671895365474
C -7.16557803331174 3.18622897801439 0.17401497815794
H -6.06428796286399 4.29561530198954 -1.39829740320744
H -7.95580110522457 3.83471447600639 0.52242569675008
B -5.44011205700117 -2.58755303660104 0.89511004887357
N -4.85219797237790 -3.45894298695620 1.89261497693673
N -6.41363294390973 -3.42141197337146 0.23206696248369
C -5.46676700293271 -4.73196500358856 1.82390899965002
C -6.40785902486522 -4.70677101477104 0.82768102053267
H -5.18098562051202 -5.54576354105136 2.47372913811466
H -7.05863724916399 -5.49741909877488 0.48449441009897
C -3.84165744352694 -3.08342433488881 2.83413731388728
C -4.22652505641142 -2.64333673841029 4.12019067762604
C -2.49080390307512 -3.04200765208011 2.40806110011368
C -3.22808402153264 -2.17386365237863 4.99903114982088
C -1.52178139001467 -2.50555018552507 3.28792737855581
C -1.89018469992446 -2.08639784050813 4.58436176567403
H -3.51072304510306 -1.83174225891215 5.99151784171264
H -0.48266749431427 -2.46005350699696 2.96946656416236
H -1.13612981201272 -1.68305153020753 5.25529505202891
C -7.31289088712715 -3.05112002494624 -0.82389738307894
C -6.83469453112753 -2.97588315747492 -2.15292892725949
C -8.65345742830032 -2.73825214697004 -0.49647372917286
C -7.72920498700131 -2.56073096341069 -3.16150803713215
C -9.52316761085403 -2.34366062935219 -1.53124457429103
C -9.06429246270720 -2.24725760920162 -2.85622031196456
H -10.55669088597483 -2.10086173378242 -1.29451925680773
H -9.74381204398856 -1.93340740209209 -3.64543293610235
C -4.31801926811993 2.10678520680853 -1.81555207515461
C -4.60706855684151 1.57062072880342 -3.09021266788151
C -2.98729471258518 2.35722416628734 -1.39855134954837
C -3.53525899297347 1.29999289074103 -3.96589194753843
C -1.93049540911896 2.01940735702641 -2.27613511057014
C -2.20885454718063 1.50461926291552 -3.56137279563647
H -3.74389495492224 0.88622613021634 -4.94940677500404
H -0.90565494892713 2.20612644880839 -1.96405839547392
H -1.39493264314802 1.25489381306384 -4.23528916883208
C -8.51831556538368 1.33996890751440 1.71351651250436
C -9.11296955137333 0.86691926456679 1.30341227106709
C -7.42601836697374 1.27112276822471 3.06349145879029
C -9.97333908521139 0.32110015045708 2.27533695595380
C -8.30476083093724 0.69618756625900 4.00562668938815
C -9.57114898412691 0.22724038239103 3.61849577423106
H -10.95343562162640 -0.04276924023422 1.97462891071422
H -7.99688337925236 0.63446615382325 5.04746223607943
H -10.24154198881313 -0.20441239216724 4.35825598515861
C -5.68844709428666 -2.59106837895877 4.50490270888694
H -6.20087372074188 -1.80008008790616 3.93840047196022
H -6.20885677015750 -3.52777969542652 4.27537741069348
H -5.80818777520002 -2.37649994514895 5.57269979181119
C -2.07969326492643 -3.61028226032378 1.06642950480221
H -2.11355021250493 -4.70842547614135 1.09061877120972
H -2.76697541603112 -3.31480569903158 0.26259682561761
H -1.05720676012078 -3.31354773342685 0.80709795306949
C -9.12718420956170 -2.80968801099733 0.93934243924697
H -9.04827317774345 -3.82775656472264 1.34158522187155
H -8.51978723649355 -2.16559930133494 1.58611807016396
H -10.16977994866288 -2.48712493324514 1.02575013054621
C -5.40195847336849 -3.32809101273500 -2.49011836650819
H -5.06870784865522 -4.21426809033454 -1.93739665149630
H -5.28954520121362 -3.52140998845757 -3.56326944750725
H -4.71822417012882 -2.51059733819738 -2.22011952651630
C -9.52223574106356 0.92714171283781 -0.15311110164241
H -8.79445694893249 0.40583079639847 -0.78623510589059
H -10.50045670326387 0.45838546201331 -0.30428468445190
H -9.57797340004571 1.96060023487944 -0.51791503824676
C -6.07900823301923 1.80658880983614 3.49632896770613
H -5.26574654090753 1.12492816352771 3.21011948079815
H -5.86186034743788 2.77056260730091 3.02080210000890
H -6.04214876708045 1.93726742571574 4.58405802547199
C -6.02713240825670 1.21081166321869 -3.46583130474202
H -6.35124439051433 0.31883375450966 -2.91043664793645

```
H   -6.73261608545790      2.01082120810477     -3.21503102951601
H   -6.10952109939918      0.99124441718103     -4.53621286889020
C   -2.69840838223815      3.01789740738907     -0.06729673320108
H   -2.89324558582118      4.09798361234943     -0.12574281685614
H   -3.35602767647162      2.64307477196460      0.72848340430130
H   -1.65095608682869      2.88180899475945      0.22288294973266
H   -7.37613005728914     -2.49635311387513     -4.18871487925414
Sn  -2.02753996669816     -0.78495701026690     -0.90824203128154
Sn  -2.33450999121725      0.26470601510360      2.04777702948861
Sn  4.93826694428393      0.58127593424711     -0.28796701562975
Sn  2.15044101463381      1.57678399441449     -0.47617499807449
B   5.73905009206008     -0.22674983960977     -2.18089300883931
B   6.01693401800400      1.66930901645008      1.30325100681700
Sn  2.87505000752125     -1.29979898587089      0.66821099823640
N   5.00537497136649     -0.14158305897429     -3.42753898757025
N   6.85664797001021     -1.08660905740956     -2.49193999707088
N   6.94460699627808      2.77543300202061      1.26631100232684
N   5.73830299739026      1.49136599132567      2.71492399737302
C   3.78321610607545      0.56812129006011     -3.64730302479309
C   5.67167884793759     -0.90406307793755     -4.41712682661488
C   6.78664675019699     -1.46752971710003     -3.85400592784009
C   7.91401849233174     -1.51094380333345     -1.61759463994351
C   7.55245012555371      3.39036684197735      0.12017870205326
C   7.20505099971189      3.20686392341428      2.59101146865556
C   6.47923014667464      2.43740688550014      3.46213842991894
C   4.88472060322090      0.48772232889294      3.27485853283801
C   3.82819224481516      1.88830564983682     -4.14526830778200
C   2.56112102515250     -0.04591750503097     -3.27376260412381
H   5.29914642788712     -0.98800409747356     -5.42727450567578
H   7.52600076244540     -2.11328252872214     -4.30439446227371
C   9.14638777065344     -0.81778142653834     -1.64987053206258
C   7.69201868365966     -2.59184610011828     -0.73283872305458
C   6.79921579738727      4.29440625159519     -0.66580061384414
C   8.89070805563257      3.06307534957970     -0.20138513114978
H   7.86842909917374      4.03283075573466      2.80098619470771
H   6.42266580475327      2.49336471692184      4.53912914331679
C   3.48177221195177      0.68839934846944      3.24464971231569
C   5.44869972264117     -0.72616871076654      3.72676525897033
C   2.61540972003822      2.59455340801424     -4.28802529400617
C   5.15600428123374      2.54981685542294     -4.43831989326081
C   1.36916306967312      0.70859489472759     -3.37510019168260
C   2.51777770120092     -1.49673107321250     -2.84128856069171
C   9.34773035188123      0.34062335057406     -2.60271026071902
C   10.16744855031552     -1.22074194105847     -0.76728669209918
C   6.37394446664585     -3.33409803429421     -0.72450882551763
C   8.73123986339061     -2.96063092182000      0.14637997450014
C   7.41157122124138      4.86133115780009     -1.80294802761559
C   5.37474012558531      4.65216812269233     -0.30076312598382
C   9.47553390435089      3.66092914404423     -1.33466177872922
C   9.66377557511885      2.07418670785683      0.64477953909692
C   2.89395130345635      2.02917420999334      2.86003566494395
C   2.63840197456245     -0.37690821947139      3.63873998018220
C   6.94039846127159     -0.95801246280282      3.63577761332211
C   4.58369253471470     -1.74591261480756      4.17652637650610
H   2.63508974516193      3.61201644469531     -4.67075940466222
C   1.39795157102552      2.02077567731019     -3.89453070268232
H   5.01511214534585      3.50679687949354     -4.95297201160075
H   5.80439109011940      1.91338661252438     -5.05135080284796
H   5.69947306740501      2.74235358351058     -3.50227669879735
H   0.42959092162152      0.24334205554768     -3.08791476642996
H   2.65881260440873     -2.15714963989620     -3.70823160110660
H   1.55562628918228     -1.74270664854036     -2.37979286530631
H   3.33343166568787     -1.74609788615881     -2.14905355285418
H   8.62248205591021      1.13977997897190     -2.41021380324592
H   9.20974778281899      0.03298072142200     -3.64691585996982
H   10.35255040061199      0.76420467820915     -2.49952636078138
H   11.12084945653047     -0.69717280927138     -0.78170392313734
C   9.96106014611394     -2.28099942303037      0.13205411020694
H   6.01063101670823     -3.51698544427701     -1.74275183506063
H   5.59837498229820     -2.75039412544055     -0.20819269896907
H   6.46935641807497     -4.29659671300938     -0.20876591597920
H   8.57452579731754     -3.78964512800942      0.83332237053048
H   6.84510538464405      5.55782530009643     -2.41768092766391
C   8.74042758742156      4.55020555414971     -2.13674604735734
H   5.05771208777401      5.56524401777100     -0.81782198630095
H   4.67875544521375      3.84780835930988     -0.57878642264963
H   5.26569407927621      4.80575698481959      0.77942276817460
H   10.50407627766891      3.41846486570588     -1.59282473269552
H   10.65400648496080      1.88295146509953      0.21737187386918
H   9.79933843477196      2.43797138089120      1.67139359364242
H   9.13298985856875      1.11753750457559      0.71555620220439
H   3.38449809532794      2.45861069321441      1.97646198555402
H   1.81528526492316      1.95096840524828      2.68341702990348
H   3.04931631120951      2.75934767007693      3.66642290045327
H   1.55991746739997     -0.23785912929167      3.61242382639750
C   3.19108419681458     -1.58472431780964      4.11681099708983
H   7.24101751853181     -1.06646110328311      2.58338755983847
H   7.51426915242377     -0.11751872300864      4.04174466939639
H   7.23107689013505     -1.87087193802218      4.16743504186617
H   5.00593902155523     -2.68454949890328      4.52675973148389
H   0.48032861271233      2.59586980979606     -3.97721235581685
H   10.75571235812914     -2.57941910376984      0.81231615615634
H   9.20138870396959      5.00018260889048     -3.01313215051120
H   2.53379319985072     -2.39341339645167      4.42576106363765
```

3. References for Supporting Information

s1 F. Neese, The ORCA program system, *Wiley Interdiscip. Rev.: Comput. Mol. Sci*. **2012**, 2, 73–78.

s2 F. Neese, Software update: the ORCA program system, version 4.0, *Wiley Interdiscip. Rev.: Comput. Mol. Sci.* **2017**, 8, e1327.

s3 F. Neese, F. Wennmohs, U. Becker, C. Riplinger, *J. Chem. Phys.* **2020**, 152, 224108.

s4 A. D. Becke, *J. Chem. Phys.* **1993**, *98*, 5648–5652.

s5 C. Lee, W. Yang, R. G. Parr, *Phys. Rev. B* **1988**, *37*, 785–789.

s6 S. H. Vosko, L. Wilk, M. Nusair, *Can. J. Phys.* **1980**, *58*, 1200–1211.

s7 P. J. Stephens, F. J. Devlin, C. F. Chabalowski, M. J. Frisch, *J. Phys. Chem.* **1994**, *98*, 11623–11627.

s8 W. R. Wadt, P. J. Hay, *J. Chem. Phys.* **1985**, 82, 270.

s9 W. R. Wadt, P. J. Hay, *J. Chem. Phys.* **1985**, 82, 284.

s10 W. R. Wadt, P. J. Hay, *J. Chem. Phys.* **1985**, 82, 299.

s11 E. Caldeweyher, S. Ehlert, A. Hansen, H. Neugebauer, S. Spicher, C. Bannwarth, S. Grimme, *J. Chem. Phys*. **2019**, 150, 154122.
